# Conserved Noncoding Elements Follow Power-Law-Like Distributions in Several Genomes as a Result of Genome Dynamics

**DOI:** 10.1371/journal.pone.0095437

**Published:** 2014-05-02

**Authors:** Dimitris Polychronopoulos, Diamantis Sellis, Yannis Almirantis

**Affiliations:** 1 Institute of Biosciences and Applications, National Center for Scientific Research “Demokritos”, Athens, Greece; 2 Department of Biochemistry and Molecular Biology, Faculty of Biology, National and Kapodistrian University of Athens, Athens, Greece; 3 Department of Biology, Stanford University, Stanford, California, United States of America; The Centre for Research and Technology, Hellas, Greece

## Abstract

Conserved, ultraconserved and other classes of constrained elements (collectively referred as CNEs here), identified by comparative genomics in a wide variety of genomes, are non-randomly distributed across chromosomes. These elements are defined using various degrees of conservation between organisms and several thresholds of minimal length. We here investigate the chromosomal distribution of CNEs by studying the statistical properties of distances between consecutive CNEs. We find widespread power-law-like distributions, i.e. linearity in double logarithmic scale, in the inter-CNE distances, a feature which is connected with fractality and self-similarity. Given that CNEs are often found to be spatially associated with genes, especially with those that regulate developmental processes, we verify by appropriate gene masking that a power-law-like pattern emerges irrespectively of whether elements found close or inside genes are excluded or not. An evolutionary model is put forward for the understanding of these findings that includes *segmental or whole genome duplication* events and *eliminations (loss)* of most of the duplicated CNEs. Simulations reproduce the main features of the observed size distributions. Power-law-like patterns in the genomic distributions of CNEs are in accordance with current knowledge about their evolutionary history in several genomes.

## Introduction

The sequencing and comparative analysis of many mammalian genomes has indicated that at least 5.5% of the human genome is under selective constraint; of that, 1.5% is estimated to code for proteins, 3.5% displays known regulatory functions, while for the function of the rest there is little or no information available [1]. One of the most interesting findings that have arisen from comparative analysis among mammalian genomes is the discovery of hundreds of ultraconserved elements (UCEs) of more than 200 bp in length that show absolute conservation among human, mouse and rat genomes [2]. One out of four of UCEs overlaps known protein-coding genes. However, such a high degree of conservation (100%) is not expected even in exons, due to the degeneration of the genetic code. Since the discovery of UCEs, there have been efforts to identify conserved elements based on lower thresholds of sequence similarity over whole genome alignments of two or more species. Several thresholds of minimal length of conserved sequence have been used as well as the exclusion of elements inside protein-coding genes [3], [4]. Throughout this article, we use the term CNE(s) for Conserved Noncoding Elements to describe all such elements despite their specific characterization as UCEs, UCNEs, HCNEs, CNGs, CNEs etc in the related literature. We here use the specific name only when we refer to the corresponding class of elements.

CNEs are not a vertebrate innovation but are also found in invertebrate and plant genomes [5]–[7]. The vertebrate, insect and nematode CNEs are not related to each other at the sequence level [6], [8], [9]. However, a recent study has identified two elements conserved between vertebrates and invertebrates [10] and it is possible that more will be identified in the near future with the advent on new sequencing methodologies and the increasing availability of sequenced genomes. In the relatively recent evolution of vertebrates, the mean length and conservation of CNEs found therein are the highest observed [11] regarding all taxonomic groups, while the conjectured roles they have acquired are particularly important [12].

CNEs are often clustered in the vicinity of genes involved in transcriptional regulation and/or development [13]–[15]. Using microarray analysis it was reported that a large fraction of noncoding UCEs have tissue-specific expression levels and are deregulated in human cancer [16], [17]. When such elements are located in the vicinity of genes, these genes are invariably found in conserved synteny in all vertebrates, possibly due to the fact that the surrounding genomic environment of a regulation-dependent gene has to be maintained intact [18]. Gene deserts are usually enriched in CNEs [19], [20] while, in mammalian genomes, the vast majority of those elements are found at long distances from the closest genes, exceeding in some cases 2 Mb, which is the limit for any known cis regulatory element [18], [21], [22]. Little is known or could be speculated about what those distant CNEs actually do. Published studies tend to support the idea that they might be an essential part of Gene Regulatory Blocks (GRBs) and that they could function in a cooperative way alongside with their target genes [4], [23]–[25].

There is a corpus of literature suggesting that CNEs are selectively constrained and not mutational cold spots [26], [27]. Studies showing that CNEs might act as transcriptional regulators, e.g. enhancers or insulators, have been published [28], [29], although *in vivo* experiments of elimination of some of these elements yield viable mice [30]. A CNE from one species may drive expression in another species as shown by transgenics experiments [31], [32], although this is not a demonstration of whether a particular CNE drives conserved expression. Experiments that have been performed in order to test the same CNE in multiple species or the same CNE from multiple species in one species, have shown that although CNEs can be identified using sequence conservation criteria, the expression patterns they drive across species may show little conservation [33]–[36]. Another aspect not directly addressed herein is the existence of paralogous CNEs in vertebrate genomes. These are believed to often remain conserved having the possibility of controlling overlapping expression patterns of their adjacent paralogous protein-coding genes [37]. Paralogous CNEs are involved in the gene expression pattern of the vertebrate brain [38].

The alternative hypothesis that CNEs are horizontally transferred between lineages and accumulate during the course of long-term evolution has also been expressed [39]. Furthermore, a study has suggested that CNEs might act as Matrix-Attachment Regions (MARs) by serving as sequences that regulate the architecture of chromatin through specific binding of particular proteins [40]. An association between CNEs and phenotypic variation and disease has also been reported [41]–[43].

Long-range correlations were reported in the nucleotide sequence of the non-protein-coding part of eukaryotic genome soon after such large sequences became available [44]–[46]. In previous works, we explored the large-scale features of several classes of genomic elements, such as protein coding segments [47], [48] and transposable elements [49], [50], by studying the size distribution of inter-exon and inter-repeat distances. In most cases we found power-law-like size distributions, fractality and self-similarity, often spanning several orders of magnitude. We here apply the same methodology for the analysis of inter-CNE distances. We use published datasets, which are characterized by different degrees of evolutionary conservation, identified in a wide variety of organisms spanning vertebrates and invertebrates. We detect power-law-like inter-CNE size distributions in most cases studied. A previous study from Salerno *et al.* [51] reported the existence of a power-law distribution in the length of “perfectly conserved” sequence from mouse/human whole-genome intersection and alignment. The work we present here focuses on the *distances* of consecutive CNEs (inter-CNE spacers) for which we also propose an explanatory model. The model that we propose cannot apply to the length distribution of CNEs themselves, thus the finding of Salemo *et al.* appears to be the expression of an independent phenomenon.

Given the aforementioned detection of long-range correlations in the nucleotide juxtaposition in non-constrained sequences of the eukaryotic genome, simple molecular dynamics have been used in attempts to explain this emergent pattern. A simple expansion - modification system is shown to generate long-range correlations through the interplay of symbol duplication and symbol elimination events [52]. More recent findings on strand slippage during replication combined with point mutations shed light into homo-nucleotide tracts and microsatellites' evolution and may be a realistic implementation of the above model to genome dynamics (see Athanasopoulou *et al.* [48], where a short discussion about evolutionary scenaria generating long-range correlations is included). Here we implement a model (initially proposed in [47]) for the generation of the observed power-law-like distribution pattern of distances between evolutionary constrained genomic elements in general (protein-coding segments and CNEs). This evolutionary scenario is based on an earlier model accounting for the explanation of power-law size distributions appearing in aggregative growth of particles in physicochemical systems [53]. This mechanism, as applied in genome evolution herein, mainly involves segmental duplication (including whole genome duplication events) and loss of most of the duplicated CNEs, alongside a moderated loss of non-duplicated CNEs in some cases.

## Methods and Materials

### Datasets

We systematically investigate the chromosomal distribution of various CNEs. We include in our analysis a phylogenetically wide collection of datasets, ranging from human to elephant shark and from vertebrates to invertebrates:

- 13,736 CNEs mapped on the human genome (hg18), of various lengths, that are identical over at least 100 bp in at least 3 of 5 placental mammals (human, mouse, rat, dog and cow) [20]. The whole set is named EU100+. Specific subsets are also considered for our purposes as follows (data kindly provided by J.S. Mattick, see also Table 1): **(ia)** 8,332 EU100+ elements that are not present in fish (Fugu). These appeared during tetrapod evolution (present in frog, chicken and/or mammals) and are named EU-FR. **(ib)** 5,404 elements from EU100+ set with orthologs in fish (ancient). These are named FR. **(ic)** 1,665 elements that are present in frog but not in fish (tetrapod speciation). These are named XT-FR. **(id)** 980 elements that are present in chicken but not in frog or fugu (amniote speciation). These are named GG-XT-FR. **(ie)** 600 elements that are not present in chicken, or frog, or fugu (mammalian speciation). These are named EU-GG-XT-FR.
- 82,335 Mammalian CNEs (conserved within mammals but not found in chicken or fish) and 16,575 Amniotic CNEs (conserved in mammals and chicken but not found in fish) respectively, mapped on the human genome (hg17) [19].

**Table 1:** Summary characterization of genomes for several datasets: Power-law-like distributions of inter-CNE distances at chromosomal scale.

Dataset	Class of CNEs	Unmasked		Masked		Reference genome
		Average Extent (avg E)	Average Extent of five ‘best’ chr. (avg E-5)	Average Extent (avg E)	Average Extent of five ‘best’ chr. (avg E-5)	
**i**	EU100+	**2**	**2.38**	**2.48**	**2.98**	hg18
**ia**	EU-FR	**1.97**	**2.46**			hg18
**ib**	FR	**2.2**	**2.72**			hg18
**ic**	XT-FR	**2.32**	**2.32**			hg18
**id**	GG-XT-FR	**2.14**	**2.14**			hg18
**ie**	EU-GG-XT-FR	**1.96**	**1.96**			hg18
**iia**	Mammalian	**1.49**	**1.9**	**1.59**	**2.04**	hg17
**iib**	Amniotic	**2.2**	**2.86**	**2.25**	**2.91**	hg17
**iii**	Human/Chicken	**2.35**	**2.63**			hg19
**iv**	Human/Fugu	**2.78**	**3.26**			hg17
**v**	Human/Zebrafish	**2.46**	**2.98**	**2.31**	**2.31**	hg17
**vi**	Human/El. shark	**2.36**	**2.69**	**2.42**	**2.68**	hg17
**vii**	*D. rerio* PCNEs	**2.43**	**3.01**			#
**viii**	Insect CNEs	**1.23**	**1.23**	**1.42**	**1.42**	dm1
**ix**	Worm CNEs	**1.7**	**1.7**			WS140
**x**	Human/Rodents	**2.15**	**2.43**			hg17

Propensity for the formation of power-law-like size distributions of the inter-CNE distances as quantified by the extent (E) of linearity in log-log scale. Average values of E for all chromosomes (avg E) and average values of E for 5 chromosomes with the largest E (avg E-5) in each genome are presented. Gene-masked genomes are also included when available (for details see in the text).

#: genome-build Ensembl 42 (zebrafish).

- 4,386 UCNEs (Ultraconserved Noncoding Elements, longer than 200bp) mapped on the human genome (hg19) that display sequence identity which is consistently greater or equal to 95% between human and chicken whole genome alignments [24].
- 3,124 Human – Fugu conserved noncoding elements mapped on the human genome (hg17) with 70% identity and a score of match-mismatch up to 60 [54].
- 2,833 Human – Zebrafish CNEs mapped on the human genome (hg17) that display identity greater than 70% over at least 80 bp [32].
- 4,782 Human – Elephant Shark CNEs mapped on the human genome (hg17), with identity ranging from 71% to 98% [55].
- 4,519 PCNEs (Phylogenetically CNEs) mapped on the zebrafish genome (genome-build Ensembl 42) that are conserved across amphioxus, zebrafish, mouse and fugu [56]. These elements are unique due to the way of their identification, which is not biased by rearrangement and duplication. In addition to that, local similarity searches (versus whole genome alignments) in the genomic regions surrounding phylogenetically defined gene families have been employed in order to detect them.
- 23,651 *D. melanogaster* – *D. pseudoobscura* (insect) CNEs of 50 bp or more that are 100% conserved between these two species, mapped on the *D. melanogaster* genome (dm1) [6].
- 2,082 Nematode (worm) CNEs with mean identity of 96% between *C. elegans* and *C. briggsae* mapped on genome WS140 [5].
- 2,614 Noncoding elements marked by extreme human-mouse-rat constraint (mapped on hg17), a subset of which act as developmental enhancers [57].

For specific details about the used data sets and the subsequent treatment see File S1. In most cases, the suite of utilities BEDTools has been used for the computational analysis [58]

### Gene and CDS masking

We proceed to a complete masking of the regions characterized as genic in the human genome (hg17 and hg18). In addition, we mask flanks surrounding every gene: 5 kb at the 5' end and 2 kb at the 3' end, in order to exclude cis-regulatory elements the localization of which may be principally determined by the positioning of the regulated gene. The region located upstream of transcription start sites is usually particularly enriched in such regulatory sequences. Extended flanks of 10 kb and 100 kb have also been masked in a similar manner (see Results section). We use custom scripts and BEDTools in order to perform the masking. In the case of *D. melanogaster*, when we refer to masked CNEs of insect origin, we refer to elements that do not overlap exonic sequences and splice sites, as adopted from the supplementary material of Glazov *et al.* [6]. For masked genes' genomic coordinate data (file format and availability) see in the File S1.

We do not proceed to the masking of other genomic components, such as transposable elements (TEs), for which there are indications that they do follow power-law-like distributions, because there is no evidence about CNE – TE functional interaction or systematic co-localization. Only a tiny proportion of TEs is reported to have been exapted to the role of a CNE, but they are too few to influence and reshape the whole CNE distribution [59].

### Size distributions

Suppose there is a large collection of n objects (in our case spacers between CNEs), each characterized by its length S. In typically random such collections (like runs of heads in a coin tossing experiment) we can approximate the distribution of sizes with an exponential distribution. Let p(S) the probability of a spacer having length between S-s/2 and S+s/2 (where s is the size of the bin width) and N*(S) the number of spacers:
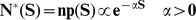
(1)


When scale-free clustering appears, long-range correlations extend to several length scales (ideally, in our case for the whole examined genomic length) and the spacers' size distributions follow a so-called power-law, which corresponds to a linear graph in a double logarithmic scale:
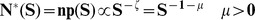
(2)


In this article we use the “cumulative size distribution”, more precisely: the complementary cumulative distribution function [60], defined as follows:
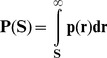
(3)where p(r) is the original spacers' size distribution. The cumulative distribution has in general better statistical properties, as it forms smoother “tails”, less affected by statistical fluctuations. Also, by definition it is independent of any binning choice: in a cumulative curve the value of P(S) for length S is not associated with the subset of spacers whose length falls in the same bin, as in the original distribution, but it corresponds to the number of all spacers longer than S. For reviews on power-law size distributions, their properties and alternative forms see e.g. [60]–[64].

The cumulative form of a power-law size distribution is again a power-law characterized by an exponent (slope) equal to that of the original distribution minus 1: if 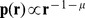, then 
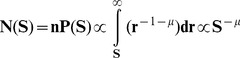
(4)where N(S) is the number of spacers longer or equal to S. All the distribution plots presented in this article depict complementary cumulative size distributions of distances (spacers) between consecutive CNEs. The logarithms of these spacers' length (S) are shown in the horizontal axis and the logarithms of the number N(S) of all the spacers longer or equal to S are shown on the vertical axis.

The slope for a typical power-law does not exceed the value of µ  =  2, as µ < 2 is a condition leading to a non-convergent standard deviation. In the power-law-like linearities reported in what follows the value of µ is always below 2. Power-law-like distributions in nature always have an upper and a lower cutoff, which determine the linear region in log-log scale, where self-similarity and fractality is observed. The extent of the linear region (E) measures the orders of magnitudes that the fractal geometry spans. Linearity has been determined by linear regression and the associated value of r^2^ is in all cases higher than 0.97 and in more than 90% of the cases higher than 0.98.

Additionally to genomic spacers' size distributions, all figures also include a bundle of ten surrogate simulated size distributions (continuous lines) where markers representing CNEs are randomly positioned in a sequence. The number of the randomly positioned markers and the length of the simulated sequence are equal to the number of CNEs and the size of the considered chromosome respectively. The inclusion of these random (surrogate) data sets in the figures visualizes the difference between observed distribution patterns and the ones expected on the grounds of pure randomness. Note that, whenever gene-masking methodology is applied, corresponding surrogates are being made that exclude the masked space from the random positioning of markers.

### Simulations using the genomic duplications – CNE loss model

Simulations using an ample choice of parameter values reproduce the observed genomic distributions. In the last figure, we show characteristic cases, while in the appendix of Plot S1, some more examples are also included. Initially, 1000 markers (representing CNEs) are randomly inserted in a sequence 2 Mbp long. Part (a) of the last figure shows snapshots of the emerging power-law-like pattern as it develops through time. Complementary cumulative size distributions of distances (spacers) between consecutive CNEs are computed every 50 segmental duplication events. Each segmental duplication (SD) event involves a region with length sampled from a uniform distribution with maximum the 5% of the actual length of the simulated sequence. In all these simulations, after each SD event, a number of CNEs equal to 90% of the number of the duplicated CNEs are eliminated (denoted as: fr  = 0.9). In the part (b) of last figure, three distribution curves are presented produced after numerical simulations where the fraction fr takes the values 0.8, 0.9 and 1. In part (c) of the same figure, three distribution curves are presented again. In these simulations the fraction fr remains constant and equal to 0.9, while, in two of them, additional eliminations of CNEs are allowed, one and two after each event of segmental duplication respectively.

## Results

### Occurrence of power-law-like size distribution between inter-CNEs' distances

The main finding of this study is the widespread occurrence of power-law-like size distribution of the distances between consecutive CNEs. In our analysis we include CNE datasets from various taxonomic groups and also compare CNE populations exapted at different evolutionary stages. The studied CNEs are mapped on different genomes (human, *D. melanogaster*, *C. elegans*, *D. rerio*).

**Figure 1:**
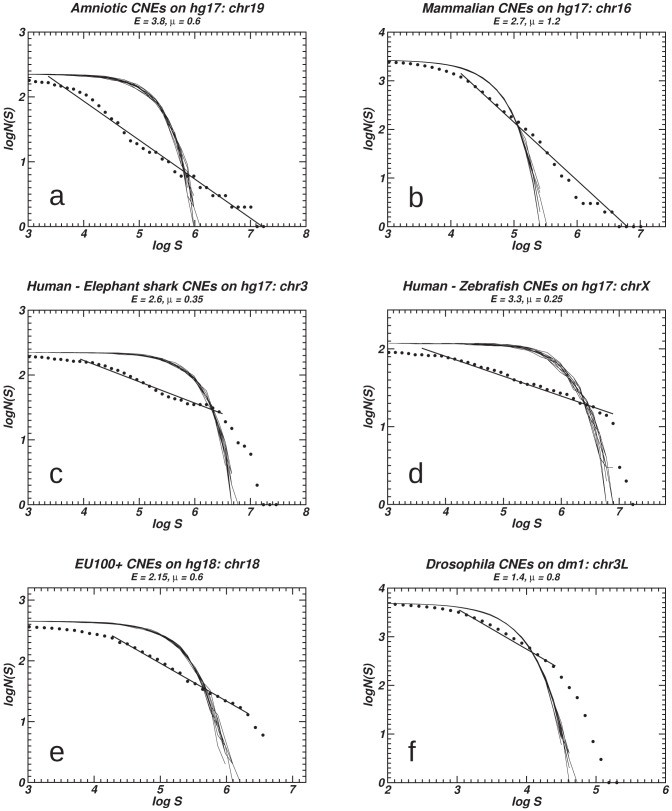
Examples of power-law-like size distributions. Twelve plots of inter-CNE spacers' cumulative size distributions in whole chromosomes. Genomic curves are accompanied in each plot by 10 curves of surrogate data (continuous lines), corresponding to randomly distributed markers. The linear segments are inferred by linear regression. Whenever we mention CNEs in the plots, we refer to the distances between consecutive CNEs.

In Figure 1 we present the size distributions of distances between consecutive CNEs in a double logarithmic plot in some typical cases. We also report the linear region E of the distribution, and the slope µ. The full set of plots is presented in Plot S1, while a complete quantitative description of our results is given in Table S1. In Table 1 we summarize the results per organism and report the average value of the linear extent E in log-log scale for all chromosomes (avg E) and for the five chromosomes with the largest E (avg E-5) (including only linear regressions with r^2^ > 0.97, see in the Methods). The extent E captures the orders of magnitude that the power-law-like distribution spans. Throughout this work we use the quantity E for measuring the existence of a self-similar chromosomal geometry and for assessing the accordance of the observed genomic distributions with the evolutionary model we propose (see Discussion).

Power-law-like patterns are not only found in alignments of closely related genomes but are widespread. Elements identified from mammalian and amniotic whole genome alignments [19] were among the first non-coding constrained elements found in quantities allowing statistical analysis of their chromosomal distribution. The complete set includes 16,575 Amniotic and 82,335 Mammalian CNEs (see Datasets iia,b, File S1 & Table S1). We observe power-law-like patterns also in collections of CNEs derived from alignments including mammals along with teleosts, their last common ancestor dated ∼450 MYA and cartilaginous fish (elephant shark) that diverged ∼530 MYA [65]. The size distribution of inter-CNE spacers in invertebrate genomes, such as *D. melanogaster* and *C. elegans* also follow a similar pattern. It is known that vertebrate and invertebrate CNEs share similar sequence characteristics but are not identical [5], hence indicating in combination with our results that their distributions are shaped by common mechanisms.

Power-law-like distributions are typically characterized by overrepresentation of large spacers. Thus, one could expect that extended power-law-like linearity would be favored in scarce data sets. However, this is not the case. Based on our data, we deduce that the power-law-like size distribution of inter-CNEs's spacers is inherent to the studied system and is not dependent on the population sizes (instances of CNEs). This is evidenced by the fact that when we reduce the numbers of mammalian CNEs (∼80,000), by random downsampling to similar numbers as the amniotic ones (∼16,000), which are characterized by more extended power-law-like linearity, the extent of linearity is not increased. Instead, linearity in double-log scale disappears as a consequence of the alteration of the studied genomic landscape. Similarly, linearity disappears when we study the merged populations of amniotic and mammalian CNEs. A description of this methodology and the related plots are included in the last section of Plot S1. Thus, we argue that our results are characteristic of each CNE class studied and are not dependent on CNE population sizes provided that the existing populations of constrained elements are sufficient for statistical analysis.

### The observed distribution of CNEs is not a mere consequence of the localization of genes in the same chromosome

**Figure 2:**
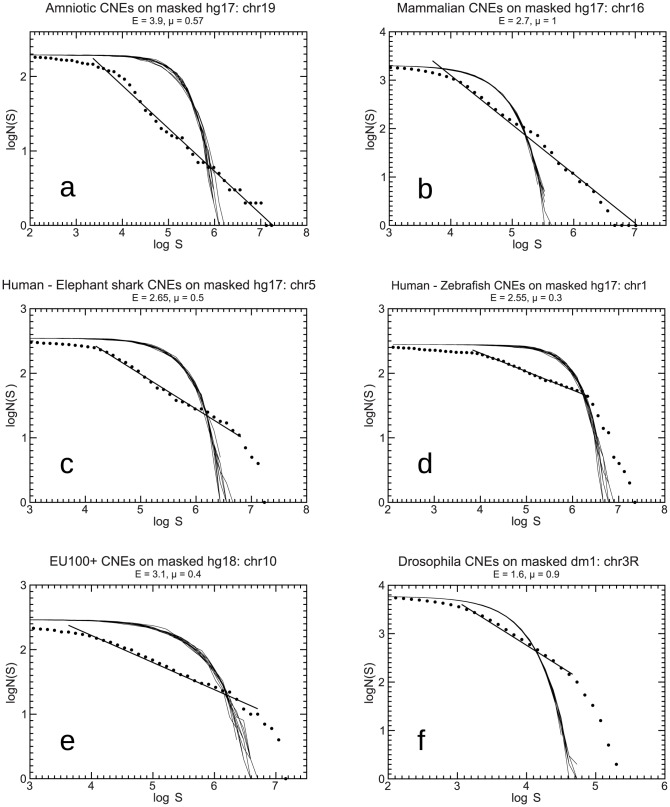
Examples of power-law-like size distributions after gene-masking. Six plots of inter-CNE spacers' cumulative size distributions in whole chromosomes after masking genes and flanks (for further details see in the text). Surrogate curves and regression as in figure 1. Whenever we mention CNEs in the plots, we refer to the distances between consecutive CNEs.

Power-law-like distributions are found in the chromosomal distribution of protein-coding segments [47]. As it is known from the literature, CNEs are somehow spatially associated with genes coding for transcription factors and developmental regulators (also known as trans-dev genes) [13], [15], [18]. To rule out the possibility that the observed CNE distributions are a consequence of power-law-like patterns followed by inter-genic distance distributions, we mask all protein coding genes and extended flanking regions, where usually most of the known regulatory elements are located. By masking, we mean excluding all the elements that fall within genes and flanking regions and not removing the genes themselves, as the latter would alter the inter-CNE distance size distribution. Linearity in log-log plots is not only preserved but in most cases improved, as shown by the increase of the linear region extent. This shows that even if we exclude from our study the CNEs that might be bound to be close to genes (thus following their distribution), the remaining CNEs still follow a power-law-like chromosomal distribution. Our principal aim here is to show that the dynamics creating the power-law-like pattern is not a mere consequence of the genic distribution, although the two distributions are expected to influence one another, a fact which is not taken into account in our simple model. Examples of such plots are given in Figure 2. The full set of these plots and the related quantitative description are also included in Plot S1, Plot S2 and Table S1. In Table 1 the results concerning “gene-masked” chromosomes per organism are also given for a direct comparison.

We choose to perform the masking methodology in the human genome for the most abundant sets of CNEs [19], [20] as well as for the most ancient elements conserved between human and zebrafish [32] or elephant shark [55]; datasets **(iia,b)**, **(i), (v)** and **(vi)** respectively. In all cases studied, we observe power-law-like size distributions of inter-CNEs' spacers that are extended over several orders of magnitude (see Table S1 & Table 1). A similar methodology is applied to the genome of *D. melanogaster*; dataset **(viii)**. The possible functions of many CNEs (individually or in blocks) through their interactions with specific genes within the nucleus, by means of chromatin looping and other conformations, has recently received a direct experimental verification through the work of Viturawong *et al.* [66]. These authors have demonstrated, in a collection of 193 UltraConserved Elements, the frequent cis action of (distant to a gene) CNEs through chromatin looping. The scope of our gene masking applied herein is not to exclude CNEs acting as distant regulatory elements through such a mechanism. Thus, we have chosen to present a moderated (5 kb upstream of the 5′ end and 2 kb downstream of the 3′ end) gene-flank masking, in order to only exclude elements, which are probably limited to act as close (e.g. promoter-like) regulators and consequently may be spatially linked to nearby genes. To further validate our claim we also performed gene-masking with extensive flanks (10 kb and 100 kb) in the EU dataset for six chromosomes (the five largest ones and chromosome 10, which is particularly abundant in CNEs). Linearity in log-log scale in the distributions of inter-CNE distances is still evident and extends at several length scales (see various statistics and plots in Table S1/sheets EU100+_masked10/100 kb and Plot S2 correspondingly). Even in the case of 100 kb flanks, such linearities are preserved, despite the few CNEs left after masking at such a large scale.

## Discussion

### An evolutionary model reproducing the observed power-law-like distributions based on genomic (segmental or whole-genome) duplications and CNE loss

Segmental duplication events occurred continuously in the evolutionary past of virtually all eukaryotes [67]–[70]. At least 10% of the non-repetitive human genome consists of identifiable (i.e. relatively recent) segmental duplication events [71]. It is estimated that 50% of all genes in a genome are expected to duplicate, giving an “offspring” at least once on time scales of 35 to 350 million years [72]. Additionally, most extant taxa have experienced paleopolyploidy during their evolution (i.e. duplication of the whole genome and subsequent reduction to diploidy), see e.g. [73], [74] and references therein. Segmental duplication and polyploidization generate copies of some or all the genes of an organism, but also of other functional genomic elements, such as CNEs. As all authors agree, see e.g. [72], [74], [75], the fate of most duplicated genes is that one copy is silenced, losing the ability to be transcribed, and then disintegrates progressively by random mutations, while it is also exposed to the possibility of excision due to recombination driven eliminations. The fate of duplicated CNEs is expected to be similar, therefore a duplicated CNE often can become superfluous and stop to be under purifying selection, being thus gradually decomposed and lost. The existence of another source of CNE loss can be supported by current findings of comparative genomics, as many of the CNEs found to be conserved between elephant shark and human are not recognized in the fugu genome (see next section for further discussion). In that case, not only duplicates of CNEs are lost but also the population of CNEs present in an ancestral organism is considerably reduced.

Occasional CNE loss of function and subsequent degradation, complete genome duplications and repeated segmental duplications alongside with other forms of genomic dynamics (e.g. insertions of transposable elements and of other parasite sequences) can be combined in an evolutionary model the propensity of which to generate power-law-like chromosomal distributions is testable through computer simulations. We implement such a model and name it “genomic duplications – CNE loss model” (see link at the bottom of File S1 where we provide the code in Fortran and a detailed description of the model). The genomic events included are:

1. Segmental duplications of extended regions of chromosomes. This step may include as limiting case whole genome duplications, although not considered in the examples shown.
2. Random eliminations of a number of CNEs which is lower or equal to the number of the duplicated ones.
3. Occasionally, additional eliminations of non-duplicated CNEs.
4. Insertions of sequences increasing the total chromosomal length (these could be transposable elements, retroviruses, microsatellite expansions etc).
5. Deletions of sequence stretches (which usually are under weak or no purifying selection).

**Figure 3:**
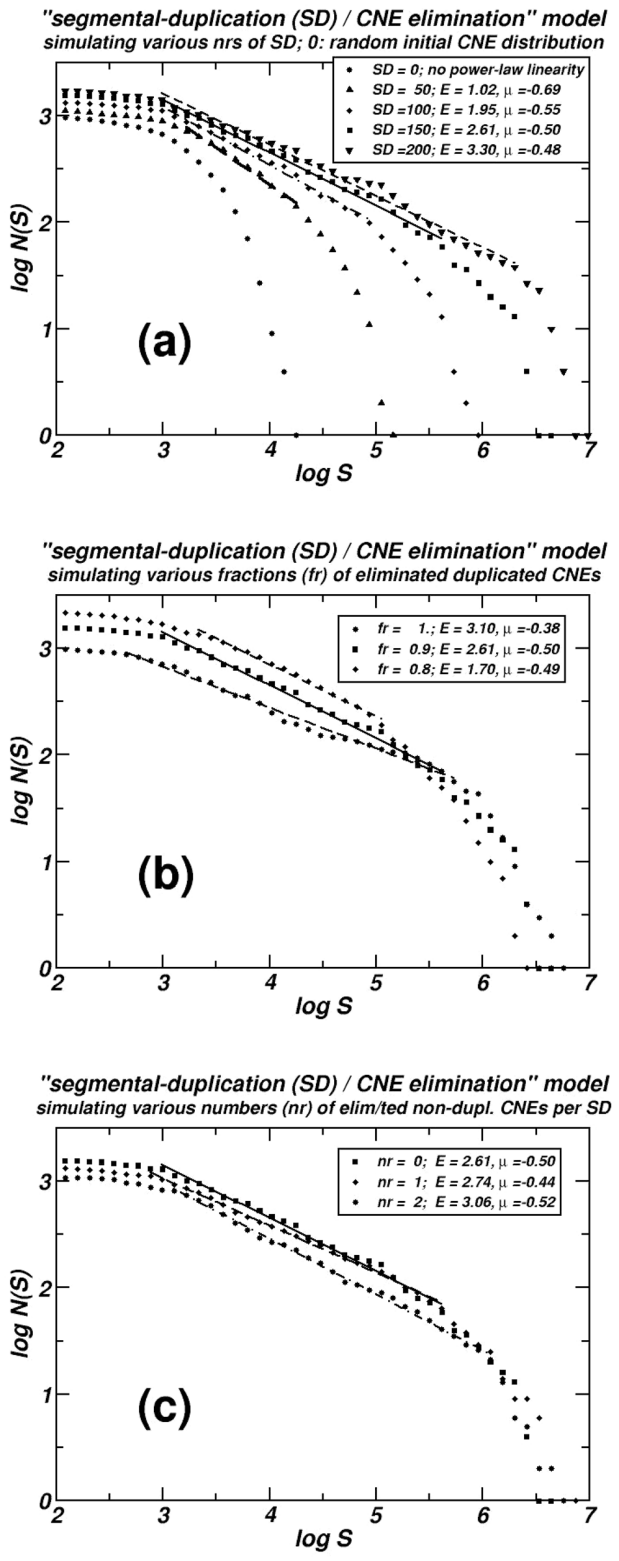
Simulations using the “genomic duplications – CNE loss model”. The dependence of the extent of the linearity in log-log scale for the distances between consecutive simulated CNEs on several parameters is shown: **(a)** The number of Segmental Duplications (SD). **(b)** The fraction of the duplicated CNEs eliminated after each SD (fr). **(c)** The number of additional, non-duplicated, CNE eliminations. In **(a)** we are able to follow the evolution of the emerging power-law-like pattern, as the four curves correspond to consecutive snapshots taken from the same numerical experiment. The curve depicted by squares (▪) is common in all three plots, representing a simulation including 150 segmental duplications, where 90% (fr = 0.9) of the number of duplicated CNEs are lost. No additional eliminations are supposed here. Linear segments are computed by linear regression and in all cases r^2^>0.98.

The proposed evolutionary model reproduces power-law-like distributions of the sizes of inter-CNE distances, see Figure 3 and additional examples of simulations in the appendix of Plot S1. This property is proven numerically to be robust to quantitative modifications of all the involved types of molecular events. Only events ***i*** and ***ii*** are indispensable for the appearance of the power-law-like pattern. This dynamics has close parallels with the one described earlier for the explanation of an analogous distributional pattern followed by protein-coding genes [47]. In a completely different genomic framework (i.e. when non-conserved elements, e.g. interspersed repeats or microsatellites are being studied), event types ***iii*** and ***iv*** (i.e. insertions of TE families more recent than the studied one) are required instead of ***i*** and ***ii*** [49], [50]. Events ***iv*** and ***v*** are numerically shown not to be required for the emergence of power-law patterns in computer simulations described herein. Inclusion of events of the type ***iv*** tests the robustness of the model, as for many organisms important parts of the genome represent repeat proliferation. Events of type ***v*** represent either deletion of sequence regions, usually due to unequal recombination or gradual shrinkage by a balance of indel events, favoring decrease of the sequence length. These types of events are of importance in genomes getting more compact (evolution occurred e.g. in the recent past of *Drosophila melanogaster* or in the case of *Takifugu rubripes*). Examples of simulations including all these types of genomic dynamics can be found in [47].

These evolutionary scenarios are based on an analytically solvable model introduced by Takayasu *et al.* [53] for the appearance of power-law size distributions in aggregative growth of particles in physicochemical systems. Notice that the model presented herein, conceived to describe the genomic dynamics of CNEs, is not analytically solvable and thus no universal exponents (slopes for the linear segment in log-log scale) may be reached. This is verified by all our computer simulations and is in accordance with the variety of slope values met in the study of genomic CNE distributions. Thus, our data deviate from the typical power-law not only because they always have the linearity in log-log scale truncated at a lower and an upper cut-off (in fact, this is a feature common to all cases of naturally occurring “power-laws”) but mainly because they lack any universal exponent (slope). This is the principal reason why we call the pattern we have found “power-law-like” throughout this article. For a recent in depth view of the requirements for having a power-law, see Stumpf and Porter [63]. These authors state that these requirements include a statistically sound power-law (extended linearity in log-log scale with indications of convergence to a universal exponent) and a concrete underlying theory to support it. In our case we clearly show that the log-log linearities we observe in our genomic data (often being quite extended) lack a universal exponent (slope). On the other hand, this deviation from universality is a characteristic feature shared between the genomic inter-CNE distributional patterns we describe herein and the simulations of the proposed evolutionary model. This feature, along with the common dependence on the evolutionary parameters shared between model and genomic distributions, corroborates the hypothesis that the evolutionary dynamics described by this model is at the origin of the observed genomic patterns.

Under a wide range of parameter combinations, our model reproduces the transient power-law-like distributions we observe in genomic data. In the simulation of Figure 3a & b, events of the types ***i*** and ***ii*** are only included: i.e. segmental duplications (SD) followed by CNE losses. In Figure 3a, a simulated chromosome is monitored using consecutive snapshots taken every 50 SD, starting from an initial (at “time zero”) random distribution of markers representing genomic CNEs. We see that, gradually, a power-law-like linear region in log-log scale appears in the cumulative distributions of inter-CNE distances, as the ones observed in real chromosomes. This plot also shows the positive dependence of the observed extent of the linearity on the number of the occurred SD. In Figure 3b, the positive dependence of the extent of linearity on the number of the CNEs eliminated (lost after each segmental duplication) is shown. Here, the number of the eliminated CNEs is expressed as a fraction (fr) of the duplicated ones, because of the segmental duplication events. Finally, in Figure 3c additional eliminations of not duplicated CNEs are simulated (events of type ***iii***), and the positive dependence of the extent of linearity on the number of non-duplicated lost CNEs is also shown. As we discuss in more detail later, this finding is compatible with the extended linearities found in the distributions of teleosts' CNEs, where important losses of ancestral CNEs are reported. Such ancient CNEs are absent in the teleost genome while retained in the tetrapod lineage.

### Evolutionary origin and implications of CNE chromosomal distributions

In the Results section we have seen that the power-law-like distributional pattern reported in the present work is proper to CNEs, UCEs and other highly conserved elements, not being a mere consequence of genic spatial distributions (see Figure 2 and Table 1). The complete study of gene-masked chromosomes has been conducted in the human genome for the most abundant sets of CNEs, as well as for the most ancient elements. In five out of six examined cases (see Table 1, datasets. **i**, **iia**, **iib**, **vi**, **viii**), inspection of both average extent of linearity for all chromosomes (avg E) and for the five chromosomes with the more extended linearity (avg E-5) reveals that the extent of the linear region in log-log scale of the original distribution follows an increasing trend (or is at least preserved) after masking of the CNEs positioned next to, or inside genes (for details see “Methods”). In the remaining one case (dataset **v**, Human/Zebrafish) well-shaped power-law-like distributions are still present after masking, with reduction of the length of the linearity. In Table S1 more information on the related statistics is given. The persistence of the linearity in log-log plots after gene-masking shows the independence of the two patterns. The fact that, in most cases, the average extent is not only preserved, but increased, further strengthens this conclusion. The frequent improvement of the power-law features when gene-masking applies, might indicate that, when the whole CNE chromosomal population is studied, the observed distributional features reflect *a superposition of two distinct dynamics*. Both include molecular events belonging to the same types but with different rates, corresponding to the distinctive evolutionary modalities of gene-uncorrelated CNEs and of genes (with which gene-proximal CNEs are spatially associated). This superposition of distributions with different features (the slope and the length scale of intervening sequences) is expected to reduce the observed linearity, because of a transformation of part of the superposed linear log-log distributions into a curved shape. Another evidence in support of the divergence between the distributional patterns followed by genes and CNEs stems from the observation reported by several research groups that UCEs and CNEs are abundant in gene deserts, see e.g. [19], [20].

A link between CNEs and Segmental Duplication, and an additional insight about the fate of duplicated CNEs is provided in the work of Derti *et al*. [76]. These authors found that segmental duplications (SD) are depleted in UCEs (100% conserved elements). They explained this finding as a result of counter selection of duplications when they contain UCEs, probably due to dosage effects. If their result is valid for constrained elements independently of degree of conservation (denoted herein as CNEs in general), this implies that we should expect a low rate of duplication of CNEs, as is the case for genes under strong dosage dependence. In our proposed evolutionary scenario, we model these rare events that over long evolutionary time may have significantly contributed to the observed distributions. Note that in most of the cases of CNEs studied herein, the time from the divergence of the studied species is sufficient for accumulation of random mutations beyond recognition for a duplicated CNE, which is no longer under purifying selection. The finding of Derti *et al.* about counter selection of duplicated CNEs may drive to the inference that, when a SD containing a CNE is fixed in a population, we may have not only relaxed purifying selection on the second copy, but additionally, due to a deleterious dosage effect a fast rate of accumulation of mutations until all the functions of the duplicated CNE are lost.

In a study of the degree of conservation of distances between UCEs in vertebrates [77], real conservation of distances is found only between closely spaced elements, a range of distances which hardly contribute to the log-log linearity reported herein (see Figure 1 and Plot S1). The absence of considerable interspecies retention of distances between conserved elements is compatible with our claim that an aggregative model (like the one described in the previous section) is suitable for the explanation of the widespread occurrence of power-law type linearities in inter-CNE distance distributions.

Mattick and co-workers, in their article on ultraconserved elements (UCEs) in tetrapod genomes, observed a striking difference in UCE populations between tetrapod genomes, which are rich in UCEs, and fish genomes, where considerably lower UCE numbers have been found [20]. They proposed as the most parsimonious explanation that in the tetrapod lineage a massive exaptation of functional elements occurred, which would probably be required for the more complex morphology and different environmental challenges met by these organisms. On the other hand, the same authors mentioned that this finding might also be explained by the less probable assumption of a massive loss of such elements in the teleost fish genome. The subsequent sequencing of the first cartilaginous fish genome (elephant shark) led to the verification of this latter scenario, as Lee *et al.* found that the jawed vertebrate ancestor had an important number of UCEs which have been eliminated (diverged beyond recognition) at great extent in teleosts, while retained in tetrapods [78]. This finding fits well with our observation that the three globally best scores in linearity extent, as deduced by inspection of Table 1 (datasets **iv**, **v**, **vii**), are all cases of alignments including teleost fish genomes. Additionally, Wang *et al.* directly correlates the observed extended eliminations of UCEs in the teleost fish with the whole genome duplication that had occurred in the ray-fish lineage [79]. Note the significance of duplications (both whole genome and segmental ones) for our proposed model. Their role is twofold, as they make possible the subsequent elimination of duplicated CNEs due to redundancy, and simultaneously provide the necessary sequence extension for the formation of lengthy inter-CNE spacers (see previous subsection).

Evidence for extensive clustering of UCNEs in the vertebrate genome, in groups which are related to the regulation of one gene each, has been presented in a recent work [24]. When loss of duplicated UCNEs occurred, the retention of UCNEs is reported to be far from the expected on the basis of a random retention model. A “winner-takes-all retention pattern” applies, i.e. one gene retains many UCNEs whereas the other paralog loses all of them more often than expected on the grounds of pure chance. An over-representation of survived fish genes, which have lost all their ancestral UCNEs, has been found. This finding is in line with our observation of extended power-law-like linearity observed in the distributional pattern emerging in alignments including teleost fish genomes. Therein, the relatively frequent eliminations of whole UCNE clusters promote the appearance of large inter – UCNEs spacers, which contribute to the long tails and thus to the formation of extended linearity in log-log scale for the corresponding distance distributions [64]. Gene-centered functional clustering described by Bucher and co-workers of CNEs seems to represent one distinct component in the spatial distribution of CNEs. This clustering extends at length scales of the order of single gene neighborhoods. The findings presented herein deals with a broader length scale, the distribution of CNEs at the chromosomal level, resulting in a variety of CNE-rich and CNE-poor domains due to the described aggregative dynamics. These domains follow a fractal-like pattern, as witnessed by the power-law-like inter-CNE distance distributions. The model we propose here consists a better null model than the random positioning of CNEs at a chromosomal level, which then, has to converge with local, gene-specific organization trends. However, the state of our knowledge about the roles of CNEs and their quantitative interactions with genes is currently limited. Further research on evolutionary dynamics and functional roles of CNEs is required in order to better understand the interweaving of the two distributional trends.

The comparison of the extent of power-law-like linearity between Amniotic and Mammalian data sets of Kim and Prichard [19] is also in accordance with the proposed model (Table 1, datasets **iia**, **iib**). We see that the older in evolutionary time Amniotic CNE collection forms the most extended linear segments, as predicted by the hypotheses of the aggregation-elimination model, where, the more the system is exposed (in evolutionary time) to the CNE elimination – sequences insertion dynamics, the more extended the linearity in log-log scale is expected to get. The same trend is clearly present in four data sets extracted from another study [20]. These datasets (Table 1, **ib** - **ie**) consist of elements exapted in consecutive evolutionary periods: tetrapod, amniote and mammalian speciations. We observe that the mean extents of linearity for the “5 best” chromosomes strictly follow the expected increasing order (from the more recent to the older), while the same holds true when we examine the mean extents for complete chromosomal sets with only one inversion found (between **ic** and **ib**).

In a very interesting work, Martinez-Mekler *et al.* [80] have shown that a broad range of systems consist of elements which, when plotted in rank *vs.* frequency or size diagrams, at semi-logarithmic scale, fit closely to a functional form including two fitting parameters. We have not further elaborated on the relation of the fitness of our data to the rank-ordering distribution approach. The range of application of this approach is quite large, and as these authors suggest, there must be an underlying explanation, possibly of a statistical nature [80]. On the other hand, our principal aim is to focus on the specific events of molecular dynamics origin, which may have caused the linearity in double logarithmic scale, and the evolutionary model proposed herein serves this purpose. A question that remains still open is the molecular dynamics impact of the fitting parameters of the functional form proposed by Martinez-Mekler *et al.*, which however lies beyond the scope of the present work. This task might be more straightforward in the case of the genomic evolution of non-constrained elements [49], [50], where the evolutionary modeling is simpler.

In what concerns not *the distances between* functional genomic localizations, but *the sizes of the localizations themselves*, we have to mention again here that a power-law size distribution of “perfectly conserved” sequences between human and mouse (repeat-masked) genomes have been observed [51]. A related finding is that the size distribution of “conserved blocks” between *Drosophila melanogaster* and *Drosophila virilis* genomes fits well to a lognormal distribution [81]. Note that this distribution also presents linear regions in log-log scale [64]. A power-law-like distribution has been reported for 3′ untranslated regions earlier [82]. These findings, concerning the sizes of functional genomic sequence stretches, have to be the result of the action of mechanisms entirely different than the evolutionary scenario applied herein, as they extend in very short length scales (tenths to hundreds of nucleotides) while any random-aggregation procedure of the type proposed herein is unsuitable for the formation of functional sequence elements. Aggregative length growth is more suitable for the shaping of large genomic regions (e.g. intervening sequences) with low conservation requirements (see also [47], [53]). However, the interweaving of linearities in the form of power-laws at several orders of magnitude and for several functional elements or the distances between them, is probably related to the fractal globule structure reported for the whole human genome when it is in the form of the tightly packed chromatin, which is characterized by extended power-law-type size distributions of the distances between points of the chromosomal thread which come in close mutual contact due to the 3D chromatin folding (see Figure 4A in [83]).

## Supporting Information

Table S1 **“Supplementary Table” (including all the examined cases).**
(XLS)

File S1 **Description of the data sets that include the chromosomal coordinates of conserved noncoding elements (BED file-format) and the corresponding data sets after suitable gene masking as described in the “Materials and Methods”.** All these data sets are also available in a compressed form via the provided URL.
(TXT)

Plot S1 **“Plots” (including all the examined cases) and additional examples of model simulations.**
(PDF)

Plot S2 **“Plots” of EU100+ CNEs on 10 kb and 100 kb gene-masked genome (hg18).**
(PDF)
