# Supplementary material for: Conserved Noncoding Elements Follow Power-Law-Like Distributions in Several Genomes as a Result of Genome Dynamics
Source: PLoS One. 2014 May 2;9(5):e95437. doi: 10.1371/journal.pone.0095437 (PMC4008492; doi:10.1371/journal.pone.0095437)
Supplement: Plot S1 — “Plots” (including all the examined cases) and additional examples of model simulations. (PDF) [file pone.0095437.s003.pdf]

Amniotic CNEs (Dataset iib)

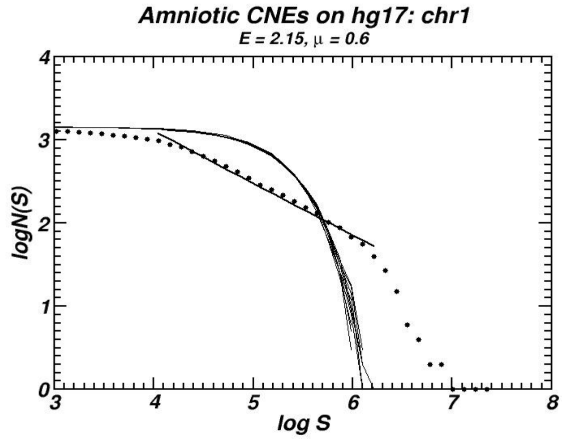

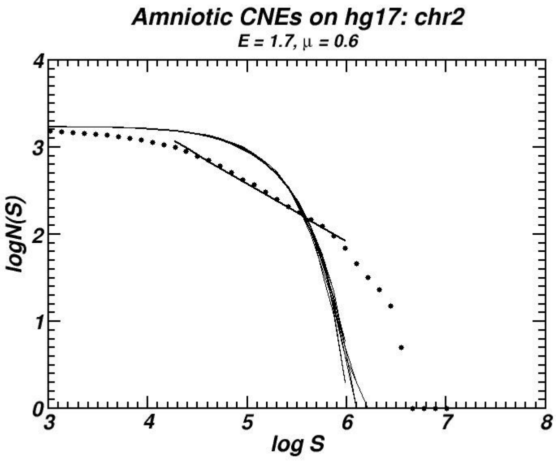

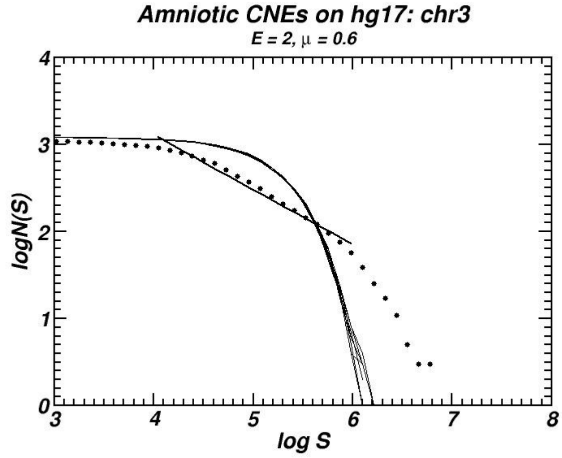

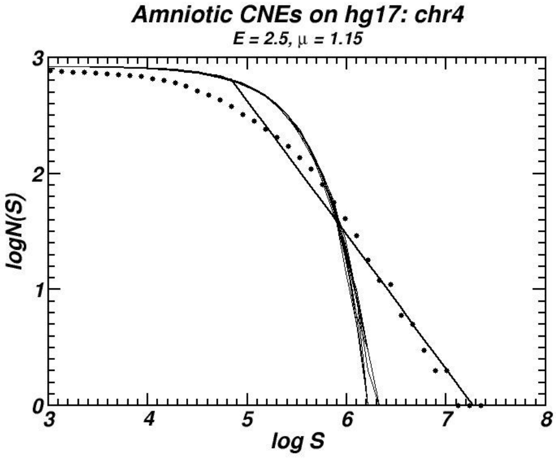

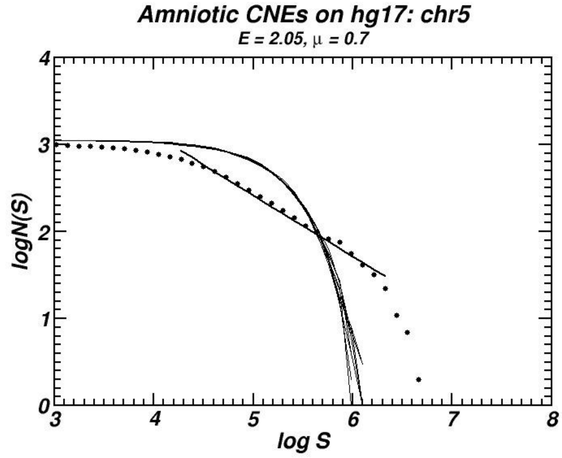

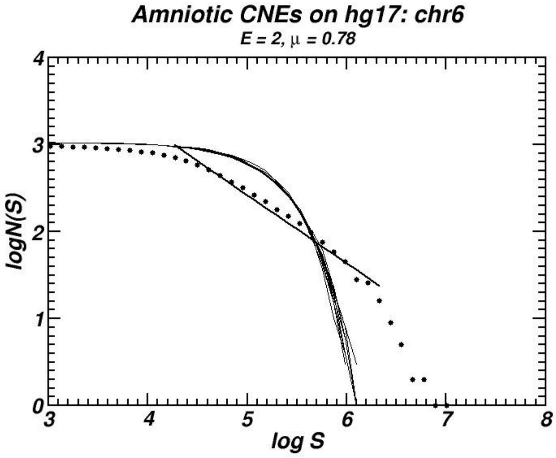

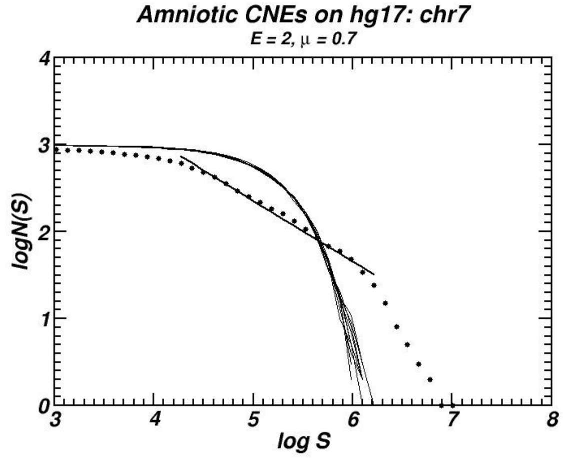
Amniotic CNEs on hg17: chr7
E = 2, μ = 0.7
logN(S)
log S

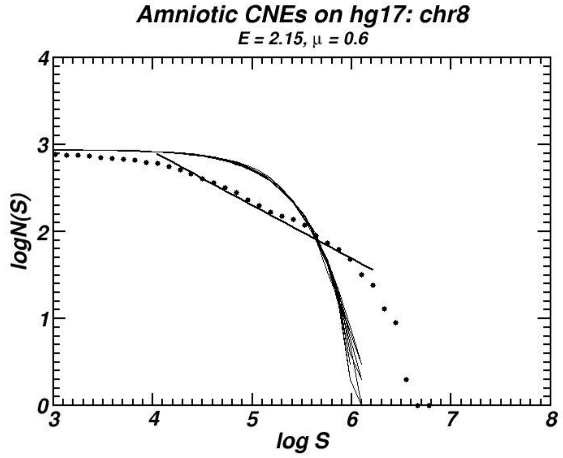
Amniotic CNEs on hg17: chr8
E = 2.15, μ = 0.6
logN(S)
log S

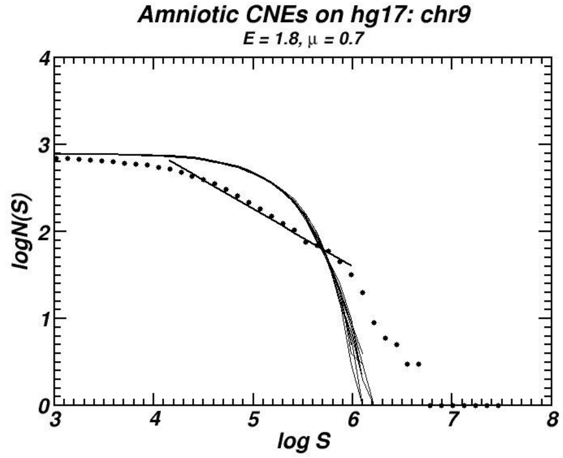
Amniotic CNEs on hg17: chr9
E = 1.8, μ = 0.7
logN(S)
log S

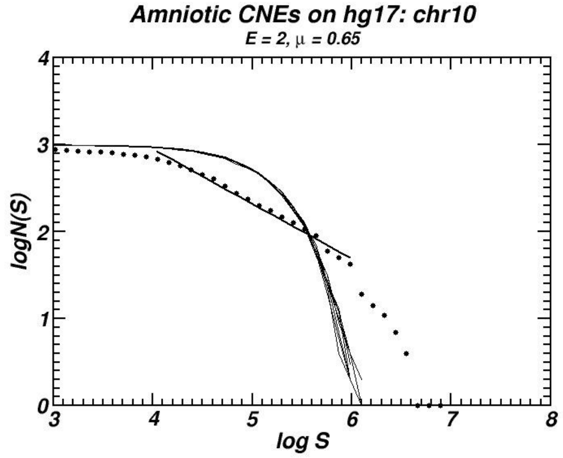
Amniotic CNEs on hg17: chr10
E = 2, μ = 0.65
logN(S)
log S

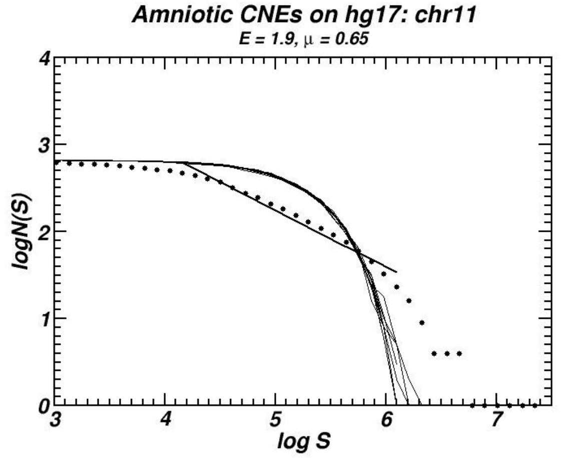
Amniotic CNEs on hg17: chr11
E = 1.9, μ = 0.65
logN(S)
log S

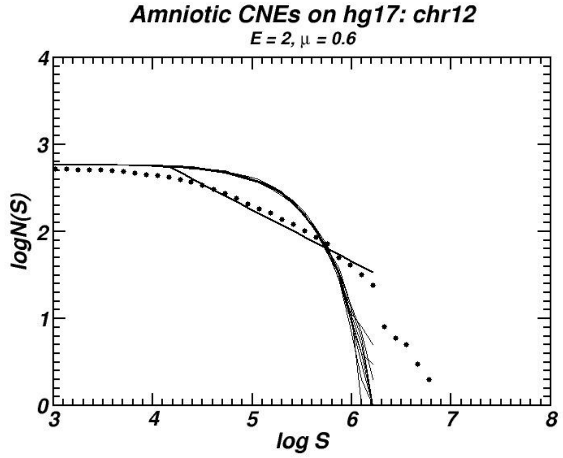
Amniotic CNEs on hg17: chr12
E = 2, μ = 0.6
logN(S)
log S

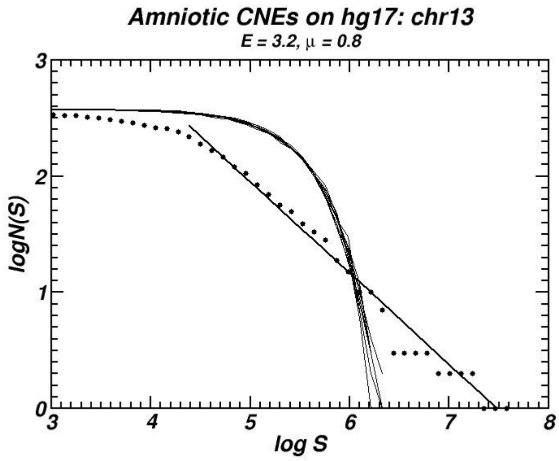
Amniotic CNEs on hg17: chr13
E = 3.2, μ = 0.8
logN(S)
log S

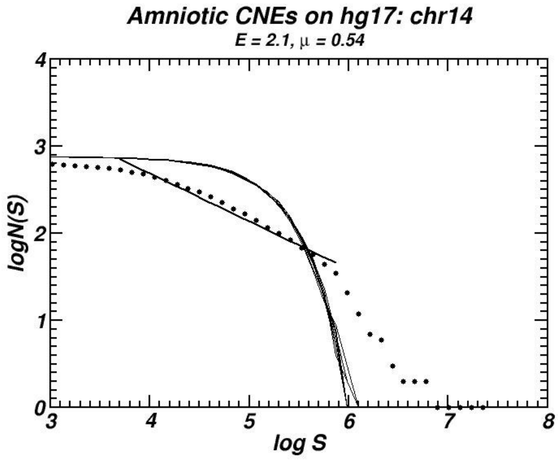
Amniotic CNEs on hg17: chr14
E = 2.1, μ = 0.54
logN(S)
log S

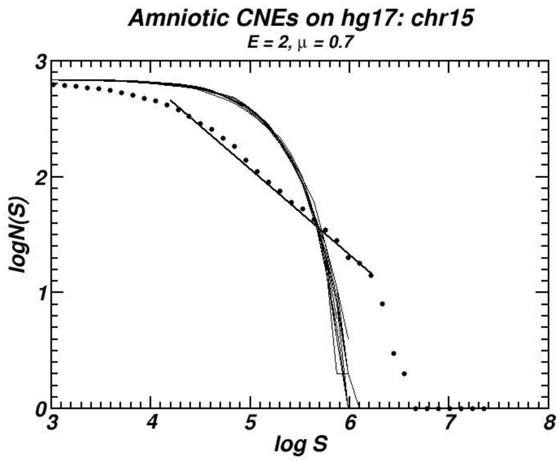
Amniotic CNEs on hg17: chr15
E = 2, μ = 0.7
logN(S)
log S

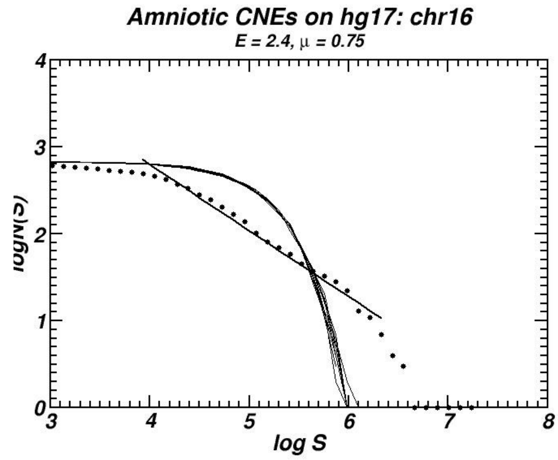
Amniotic CNEs on hg17: chr16
E = 2.4, μ = 0.75
log S

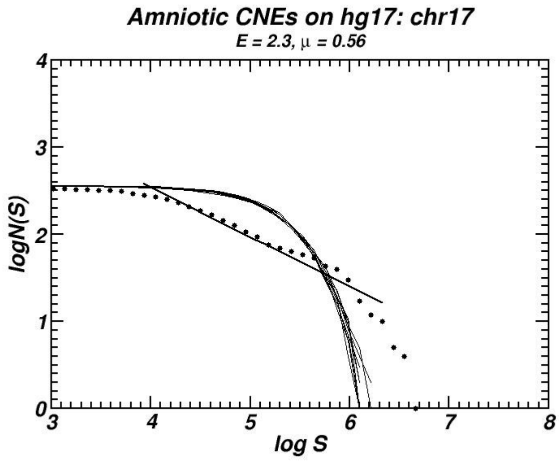
Amniotic CNEs on hg17: chr17
E = 2.3, μ = 0.56
logN(S)
log S

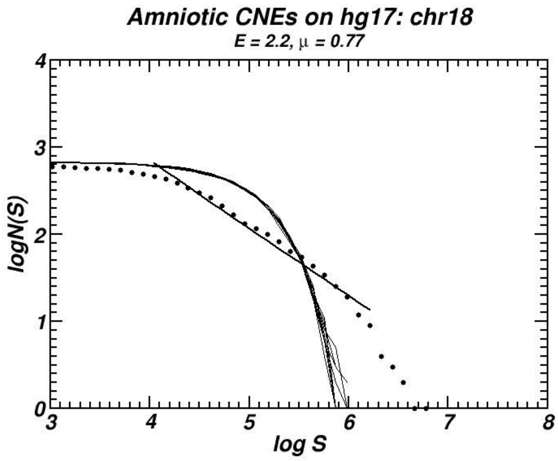
Amniotic CNEs on hg17: chr18
E = 2.2, μ = 0.77
logN(S)
log S

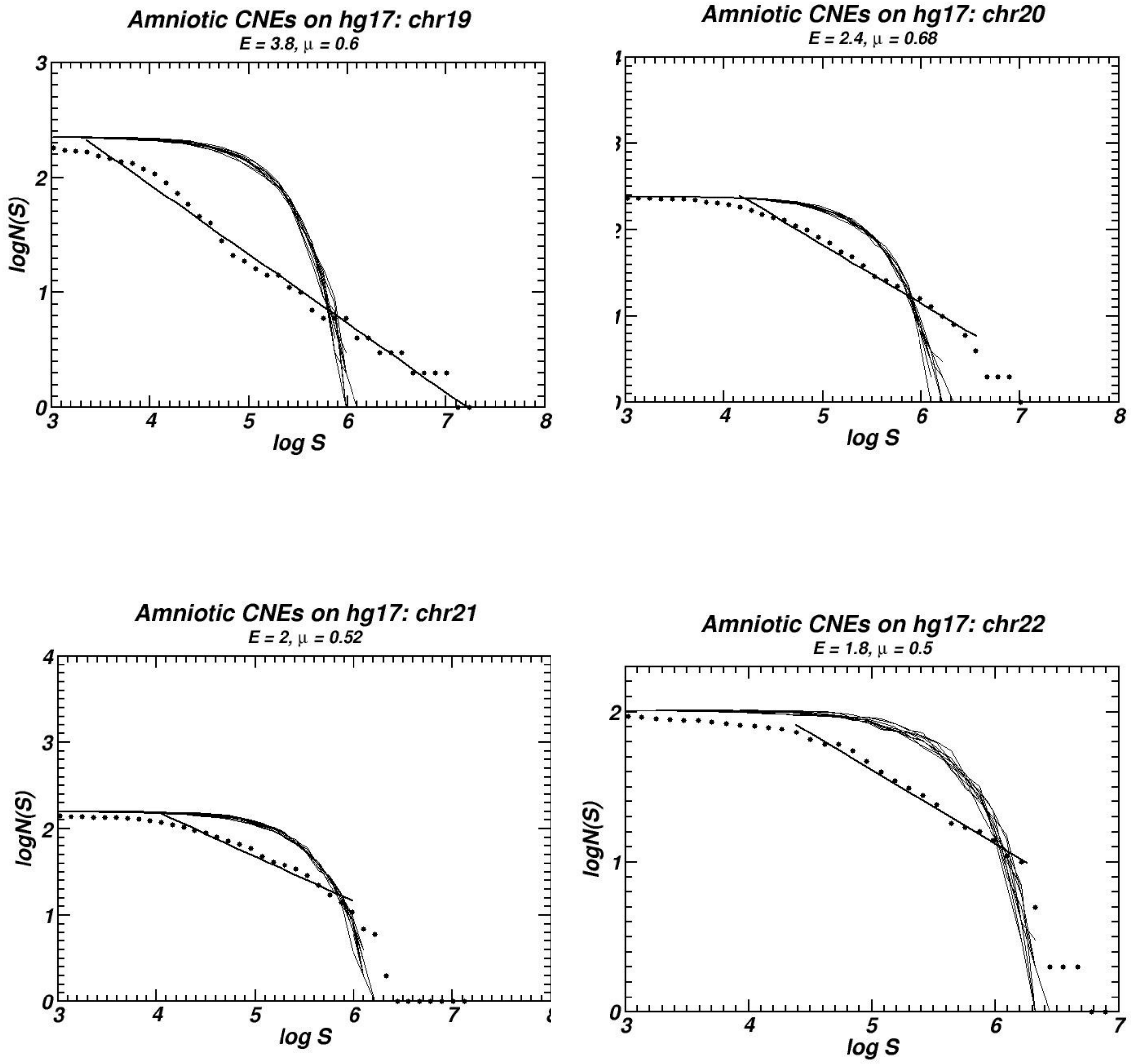
Amniotic CNEs on hg17: chr19
E = 3.8, μ = 0.6
Amniotic CNEs on hg17: chr20
E = 2.4, μ = 0.68
Amniotic CNEs on hg17: chr21
E = 2, μ = 0.52
Amniotic CNEs on hg17: chr22
E = 1.8, μ = 0.5
logN(S)
log S

Amniotic CNEs on gene-masked genome

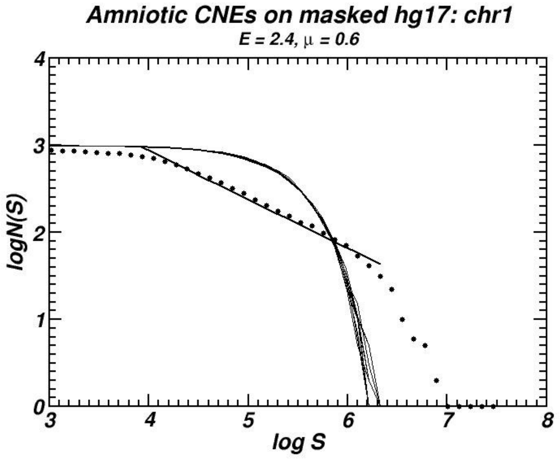

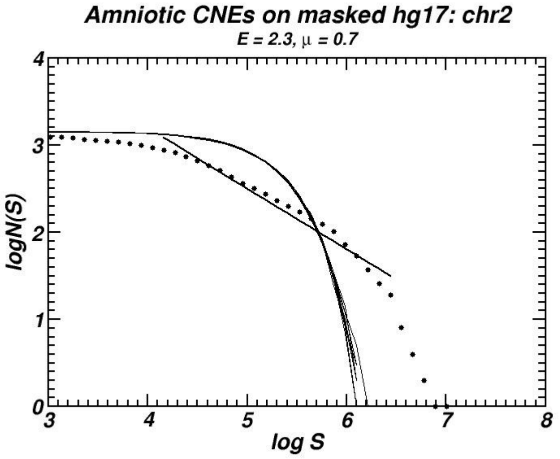

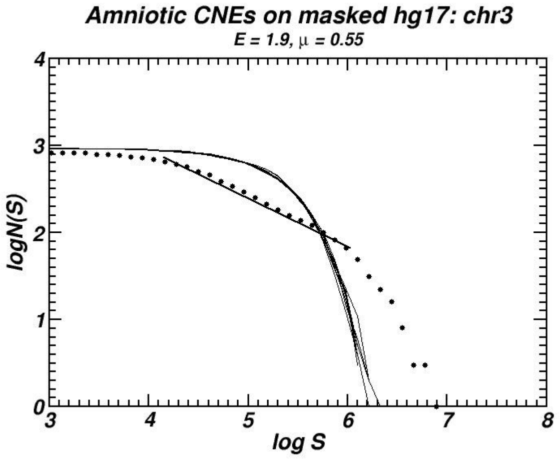

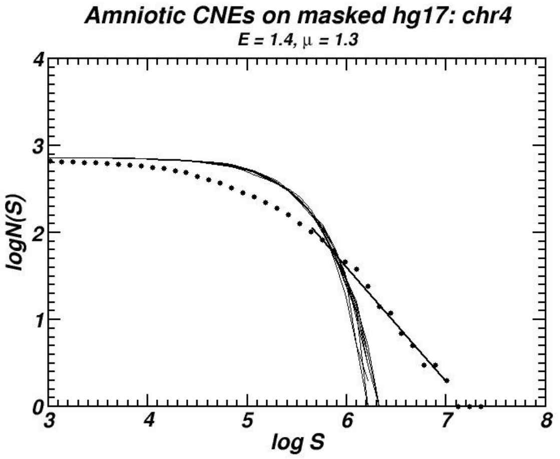

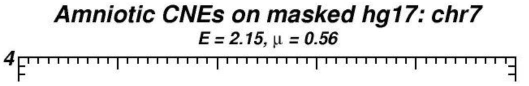

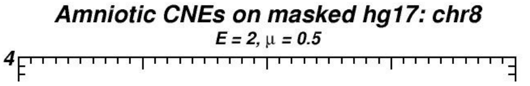

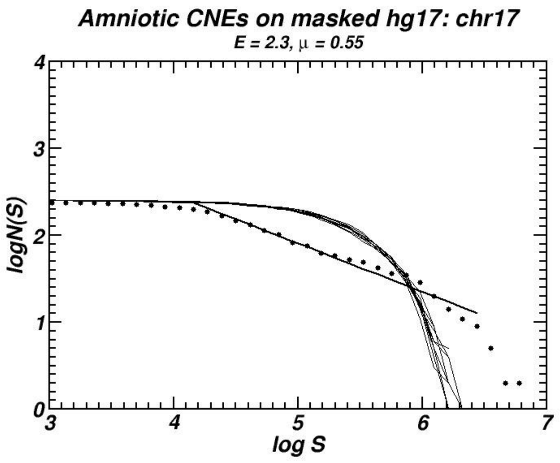

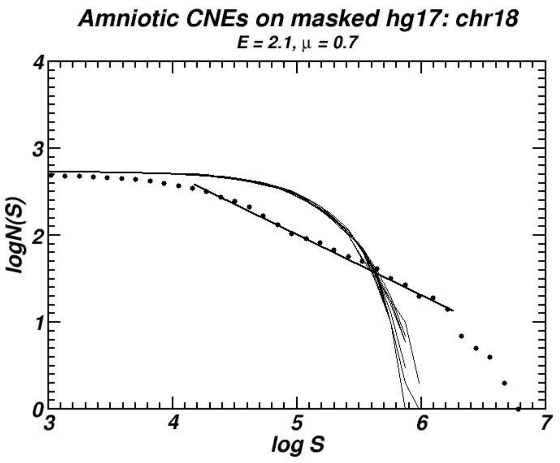

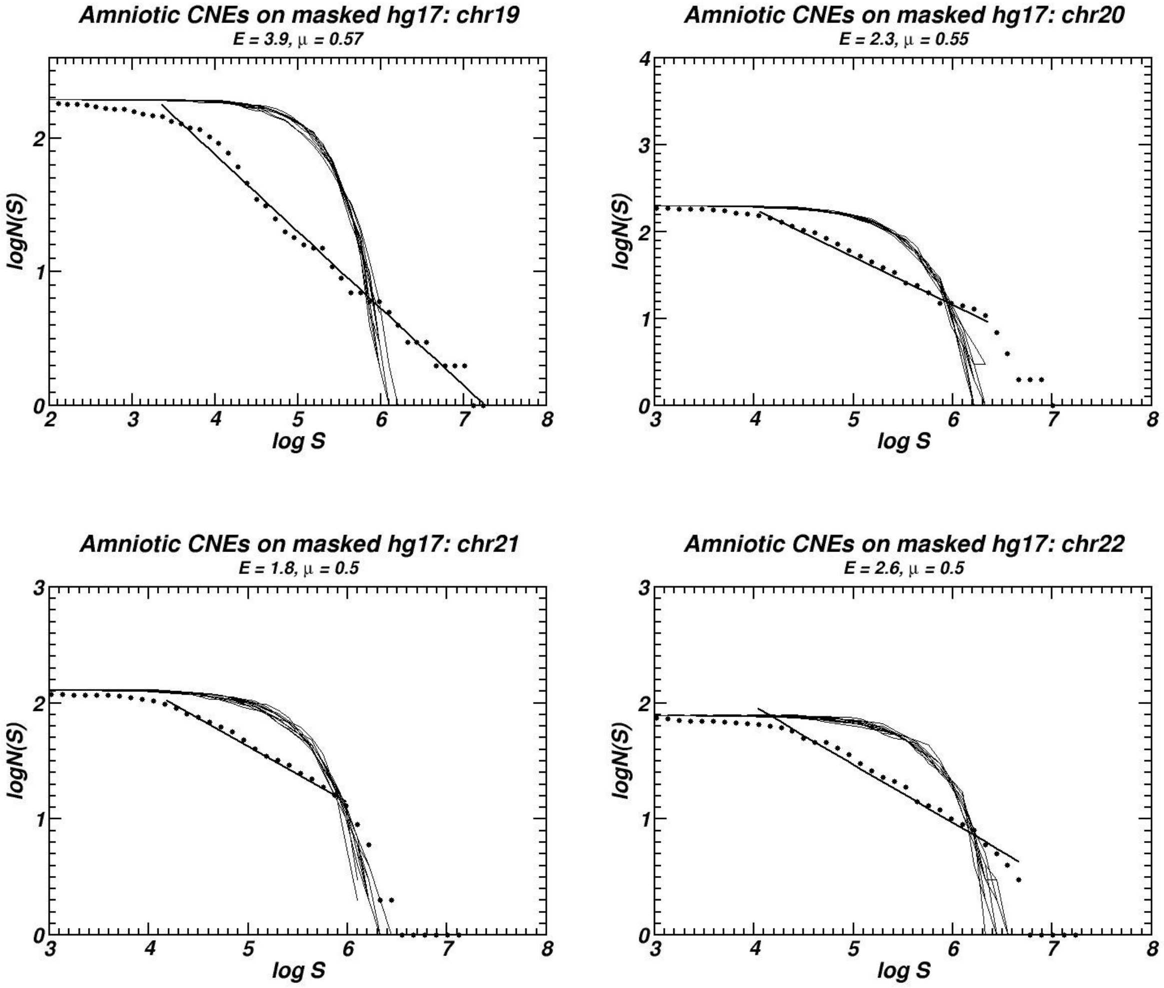
Amniotic CNEs on masked hg17: chr19
E = 3.9, μ = 0.57
logN(S)
log S
Amniotic CNEs on masked hg17: chr20
E = 2.3, μ = 0.55
logN(S)
log S
Amniotic CNEs on masked hg17: chr21
E = 1.8, μ = 0.5
logN(S)
log S
Amniotic CNEs on masked hg17: chr22
E = 2.6, μ = 0.5
logN(S)
log S

Mammalian CNEs (Dataset iia)

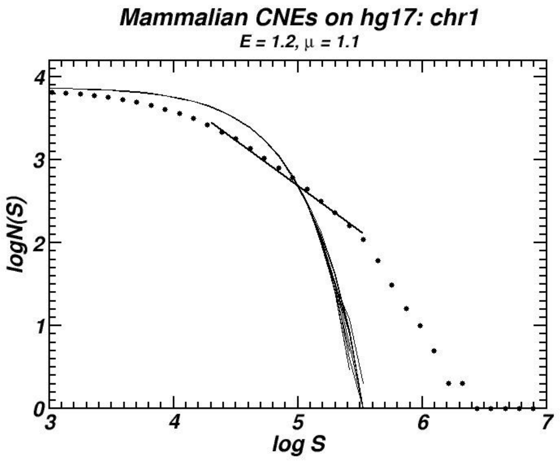

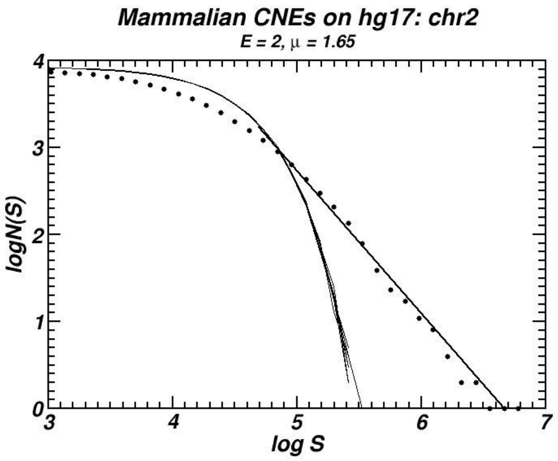

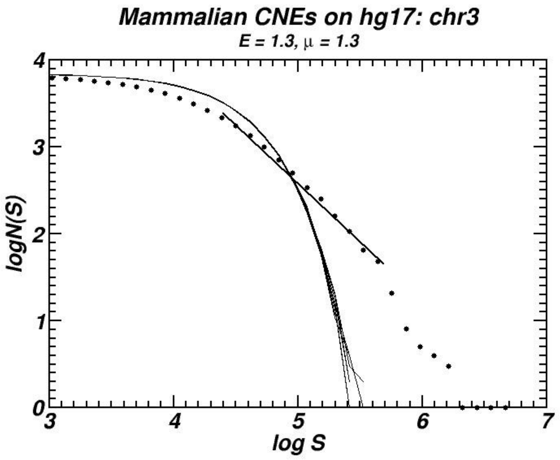

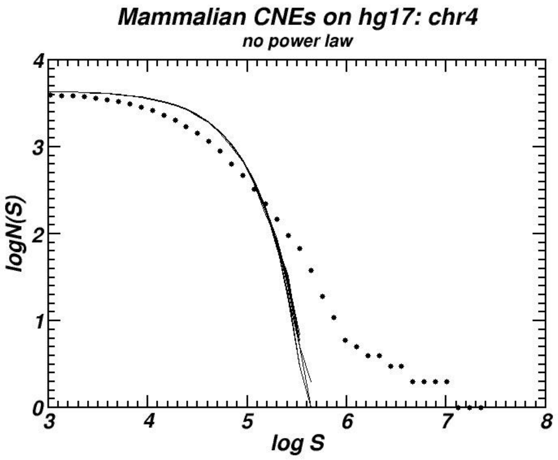

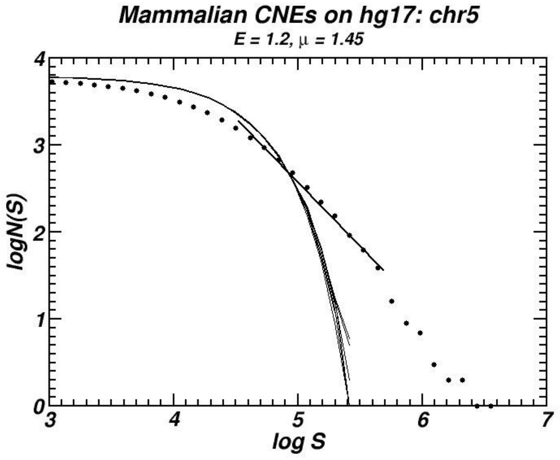

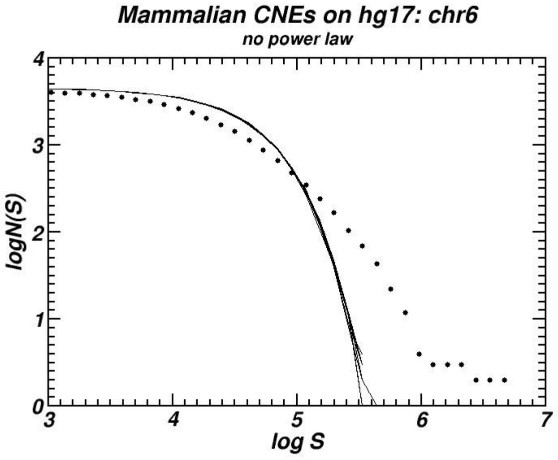

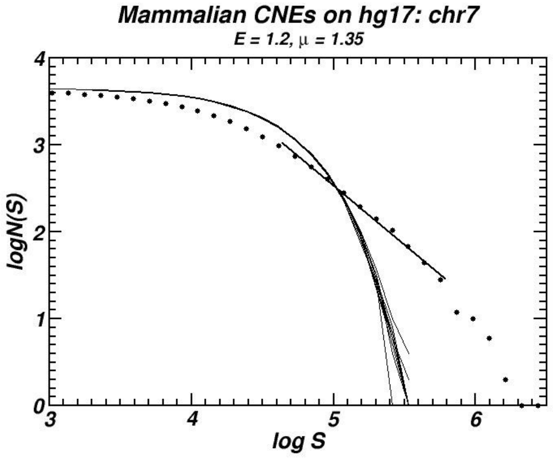
Mammalian CNEs on hg17: chr7
E = 1.2, μ = 1.35
logN(S)
log S

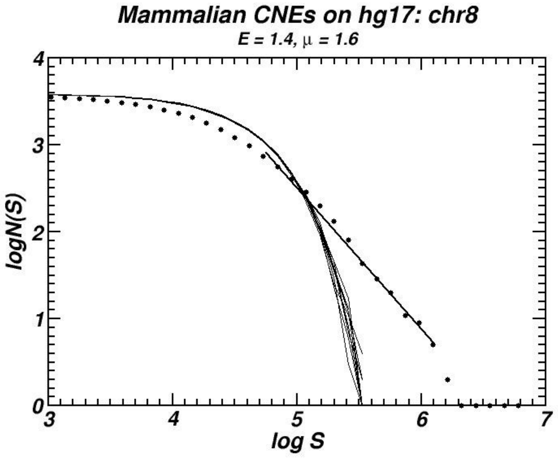
Mammalian CNEs on hg17: chr8
E = 1.4, μ = 1.6
logN(S)
log S

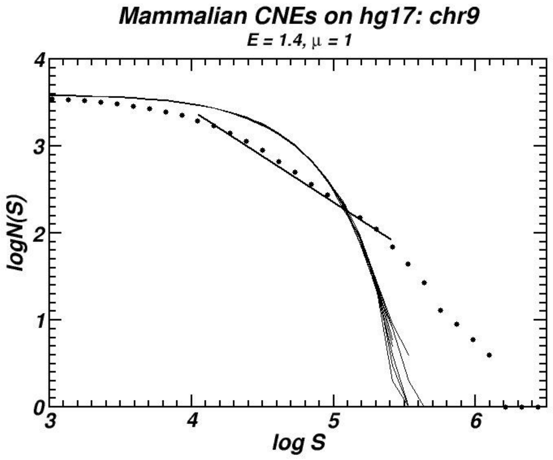
Mammalian CNEs on hg17: chr9
E = 1.4, μ = 1
logN(S)
log S

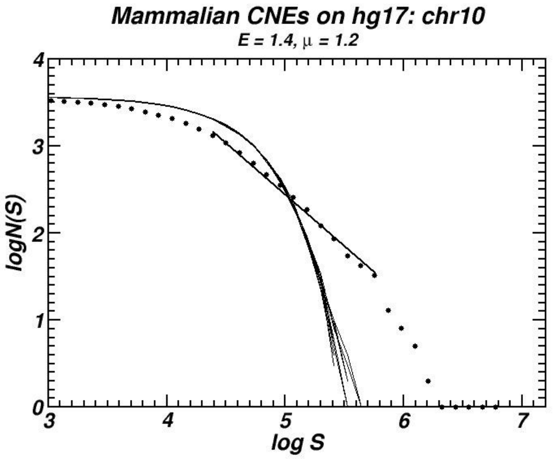
Mammalian CNEs on hg17: chr10
E = 1.4, μ = 1.2
logN(S)
log S

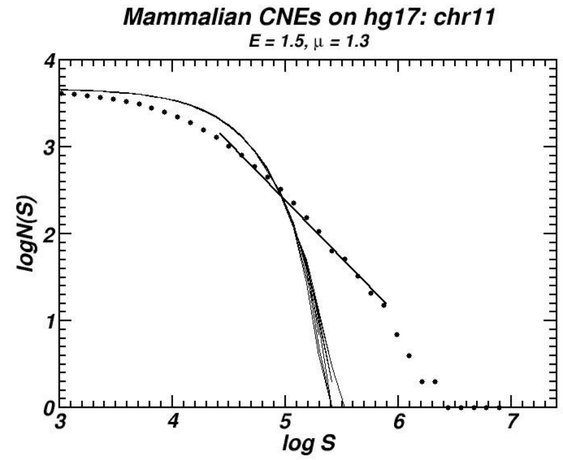
Mammalian CNEs on hg17: chr11
E = 1.5, μ = 1.3
logN(S)
log S

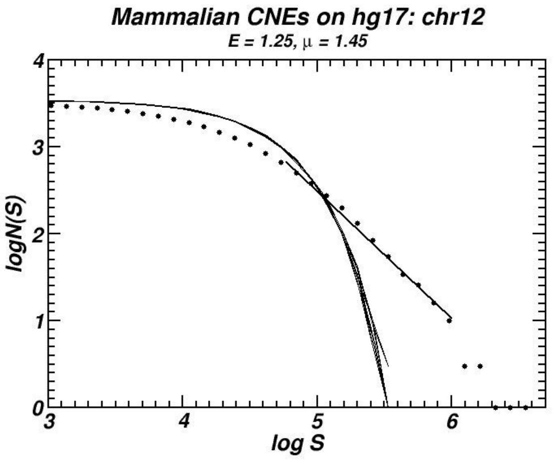
Mammalian CNEs on hg17: chr12
E = 1.25, μ = 1.45
logN(S)
log S

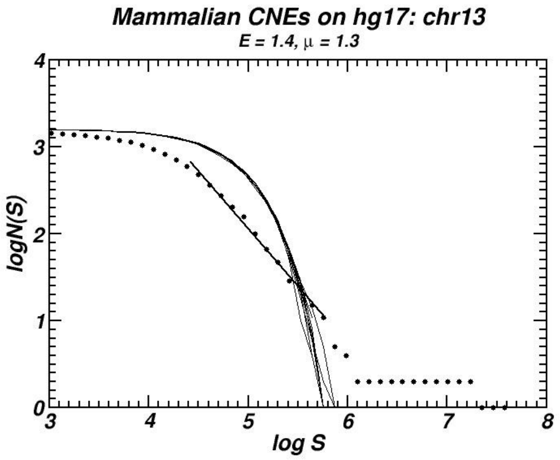
Mammalian CNEs on hg17: chr13
E = 1.4, μ = 1.3
logN(S)
log S

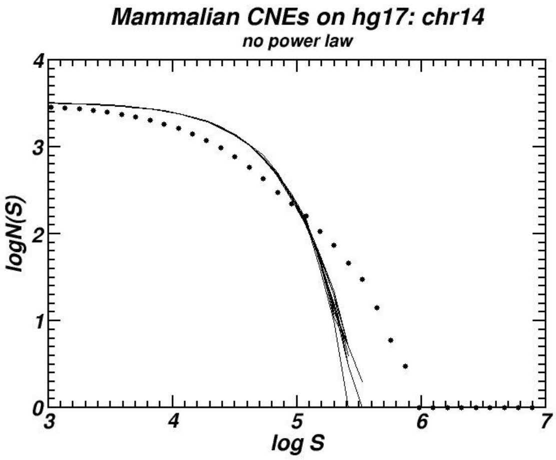
Mammalian CNEs on hg17: chr14
no power law
logN(S)
log S

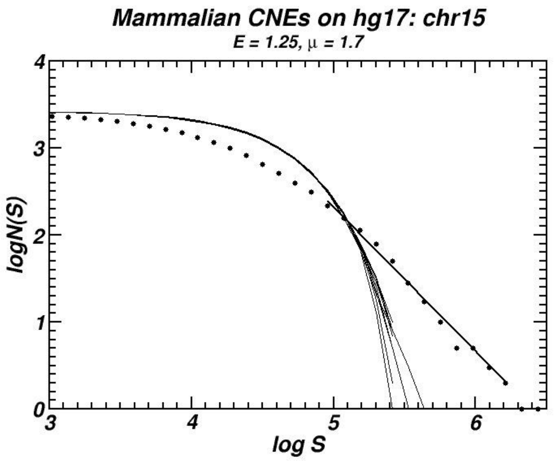
Mammalian CNEs on hg17: chr15
E = 1.25, μ = 1.7
logN(S)
log S

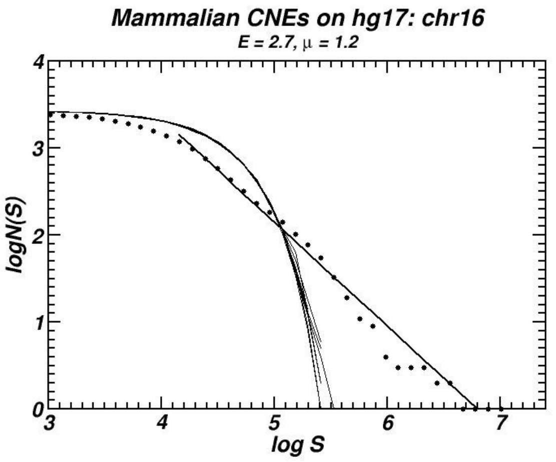
Mammalian CNEs on hg17: chr16
E = 2.7, μ = 1.2
logN(S)
log S

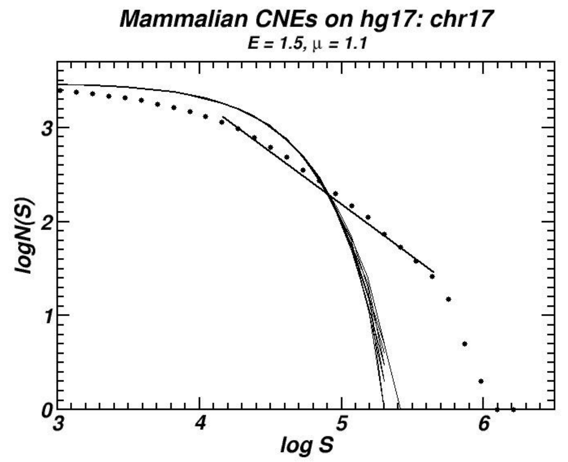
Mammalian CNEs on hg17: chr17
E = 1.5, μ = 1.1
logN(S)
log S

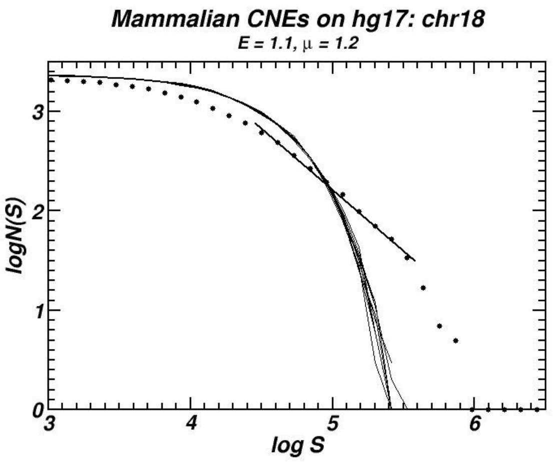
Mammalian CNEs on hg17: chr18
E = 1.1, μ = 1.2
logN(S)
log S

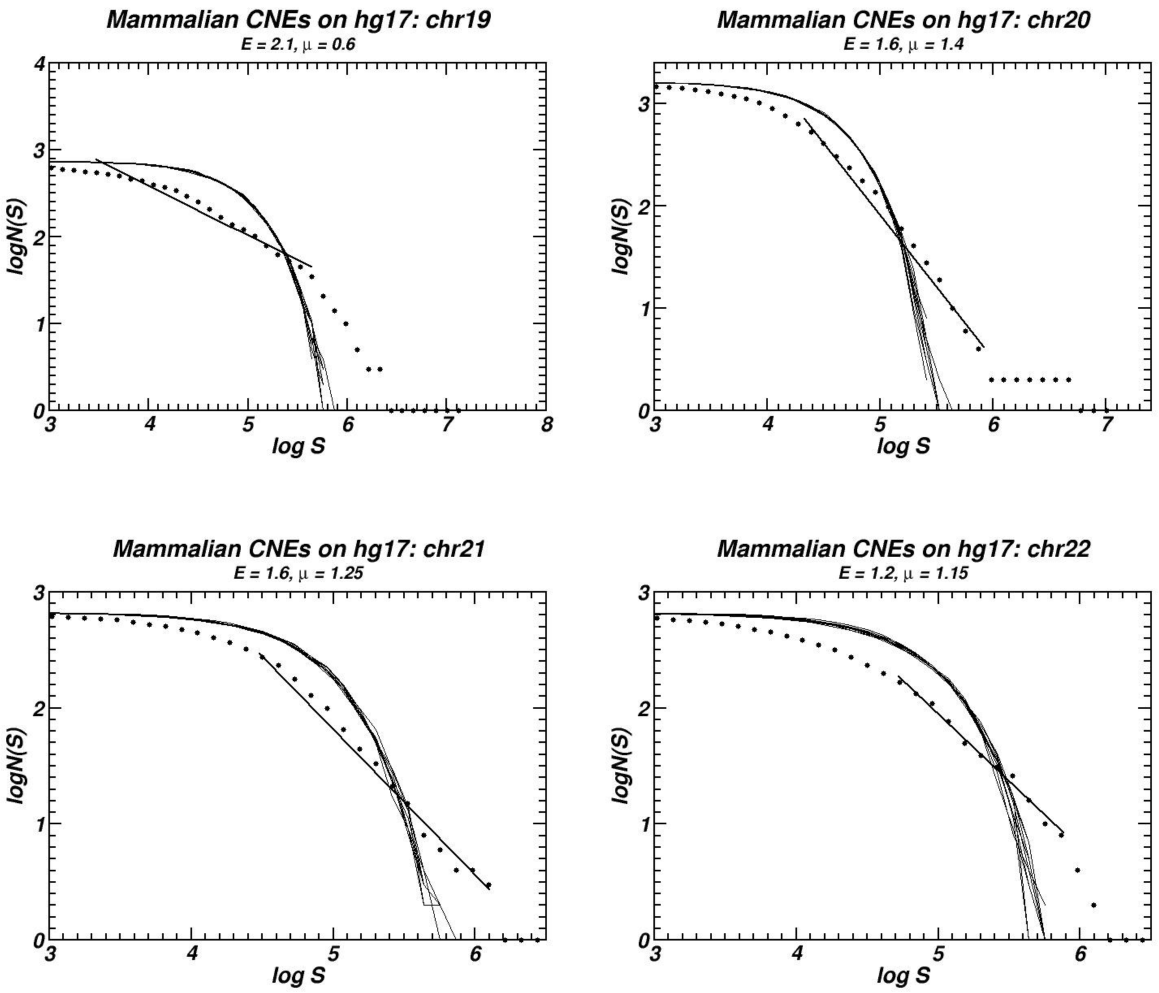
Mammalian CNEs on hg17: chr19
E = 2.1, μ = 0.6
logN(S)
log S
Mammalian CNEs on hg17: chr20
E = 1.6, μ = 1.4
logN(S)
log S
Mammalian CNEs on hg17: chr21
E = 1.6, μ = 1.25
logN(S)
log S
Mammalian CNEs on hg17: chr22
E = 1.2, μ = 1.15
logN(S)
log S

Mammalian CNEs on gene-masked genome

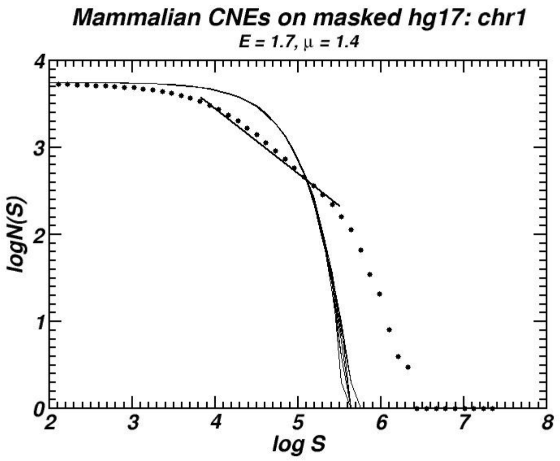

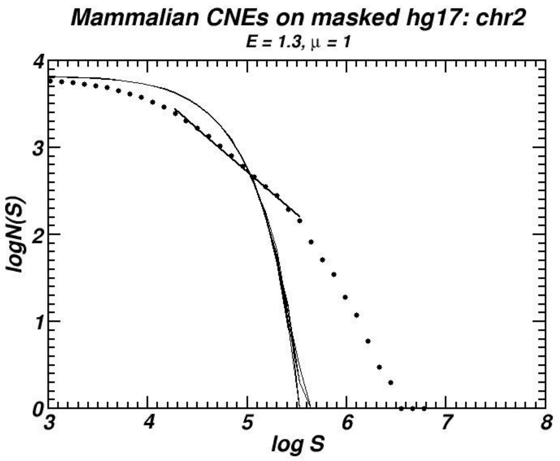

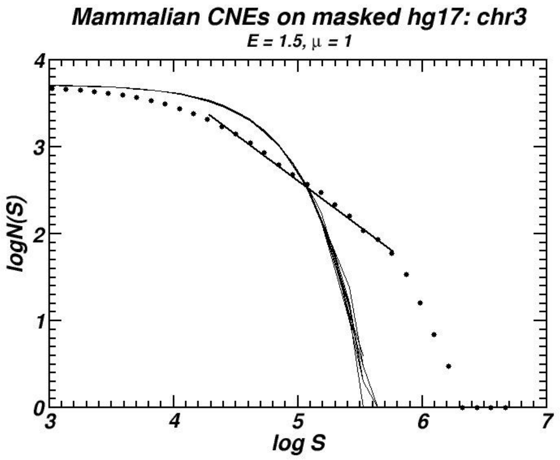

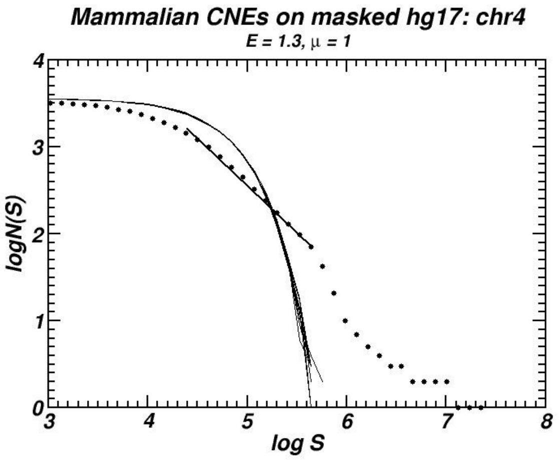

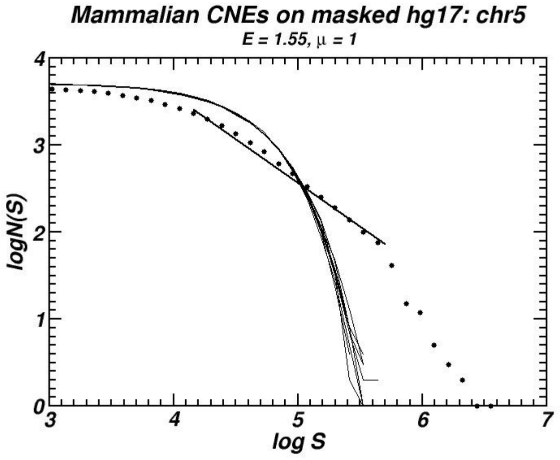

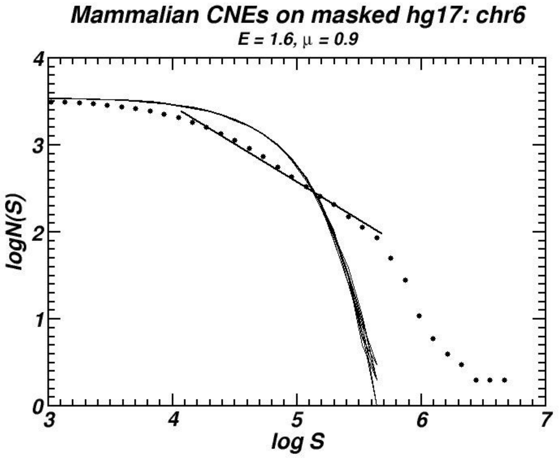

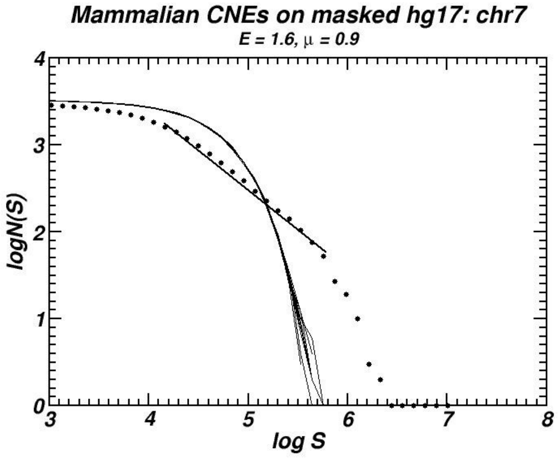
Mammalian CNEs on masked hg17: chr7
E = 1.6, μ = 0.9
logN(S)
log S

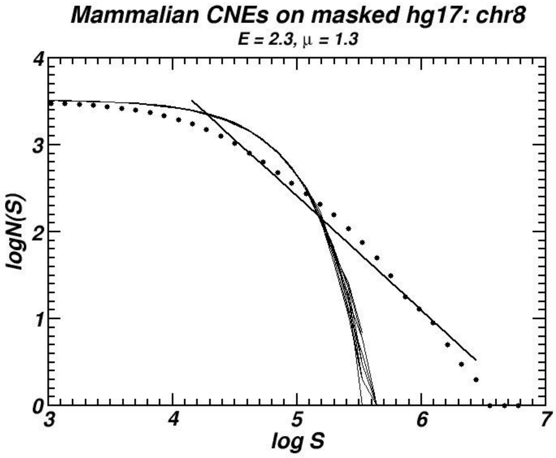
Mammalian CNEs on masked hg17: chr8
E = 2.3, μ = 1.3
logN(S)
log S

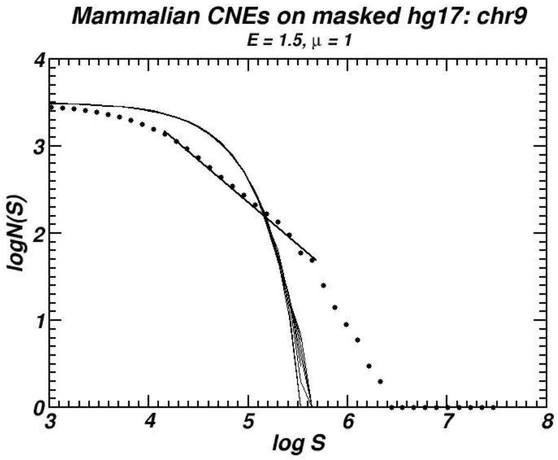
Mammalian CNEs on masked hg17: chr9
E = 1.5, μ = 1
logN(S)
log S

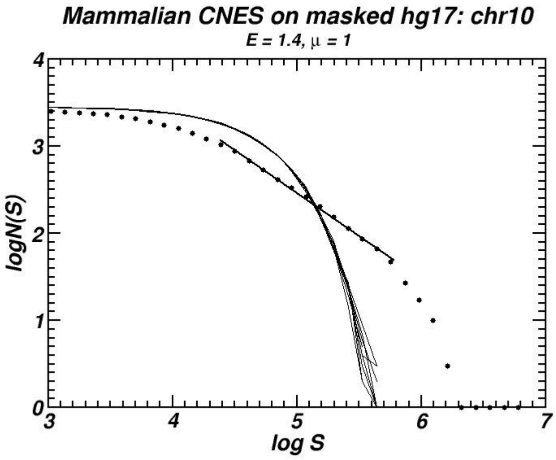
Mammalian CNES on masked hg17: chr10
E = 1.4, μ = 1
logN(S)
log S

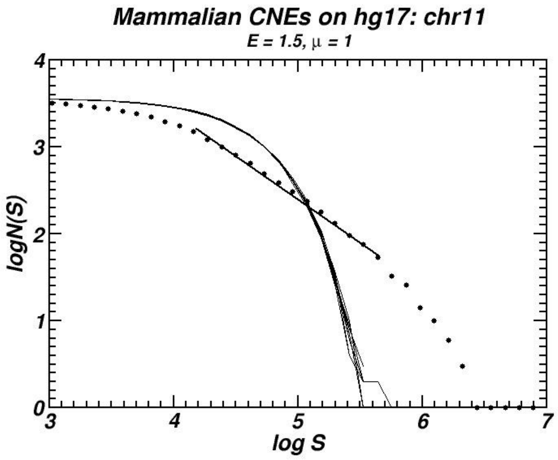
Mammalian CNEs on hg17: chr11
E = 1.5, μ = 1
logN(S)
log S

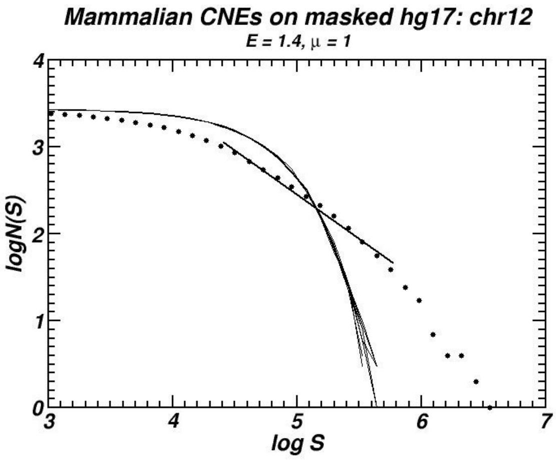
Mammalian CNEs on masked hg17: chr12
E = 1.4, μ = 1
logN(S)
log S

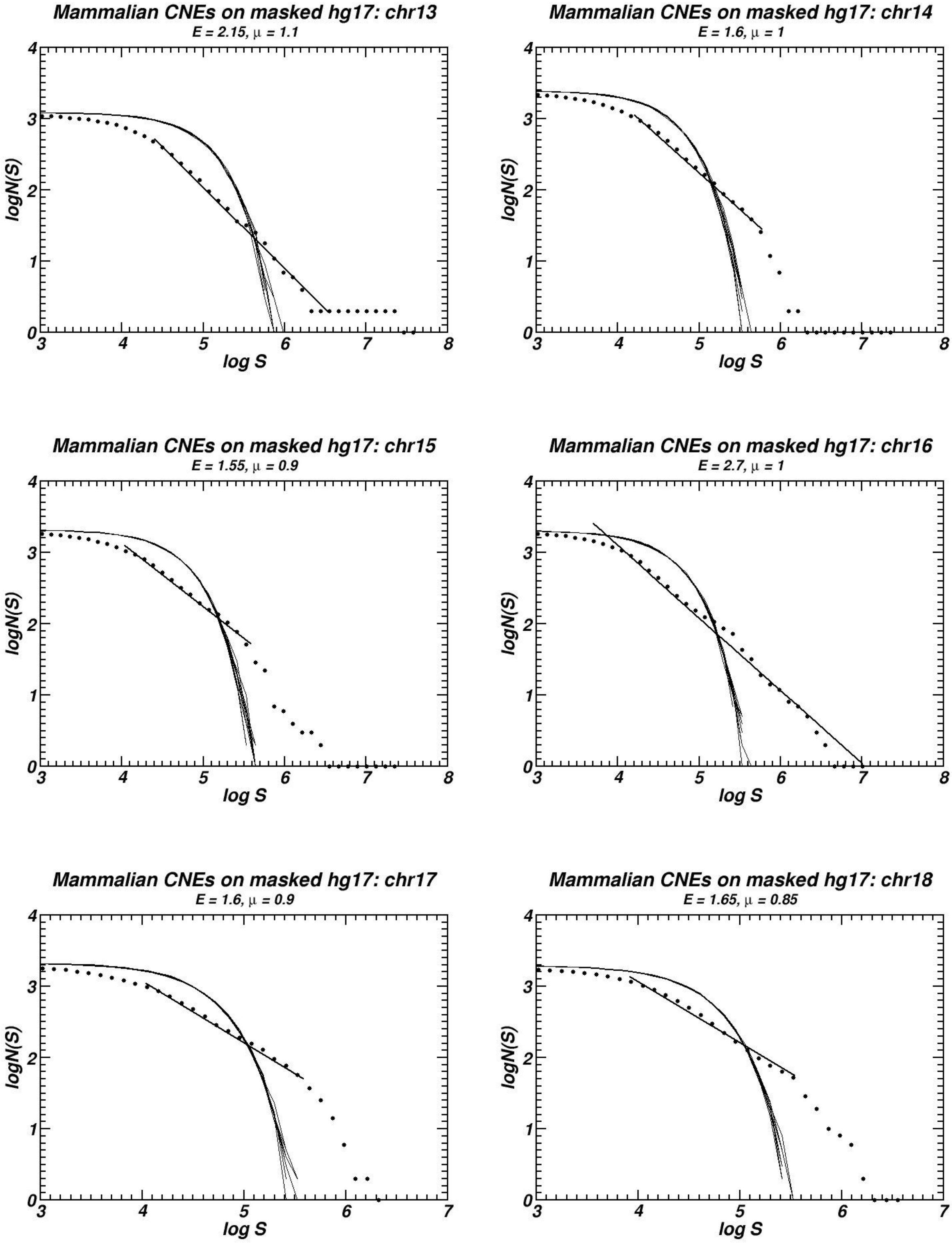

Mammalian CNEs on masked hg17: chr13
E = 2.15, μ = 1.1
Mammalian CNEs on masked hg17: chr14
E = 1.6, μ = 1
Mammalian CNEs on masked hg17: chr15
E = 1.55, μ = 0.9
Mammalian CNEs on masked hg17: chr16
E = 2.7, μ = 1
Mammalian CNEs on masked hg17: chr17
E = 1.6, μ = 0.9
Mammalian CNEs on masked hg17: chr18
E = 1.65, μ = 0.85
logN(S)
log S

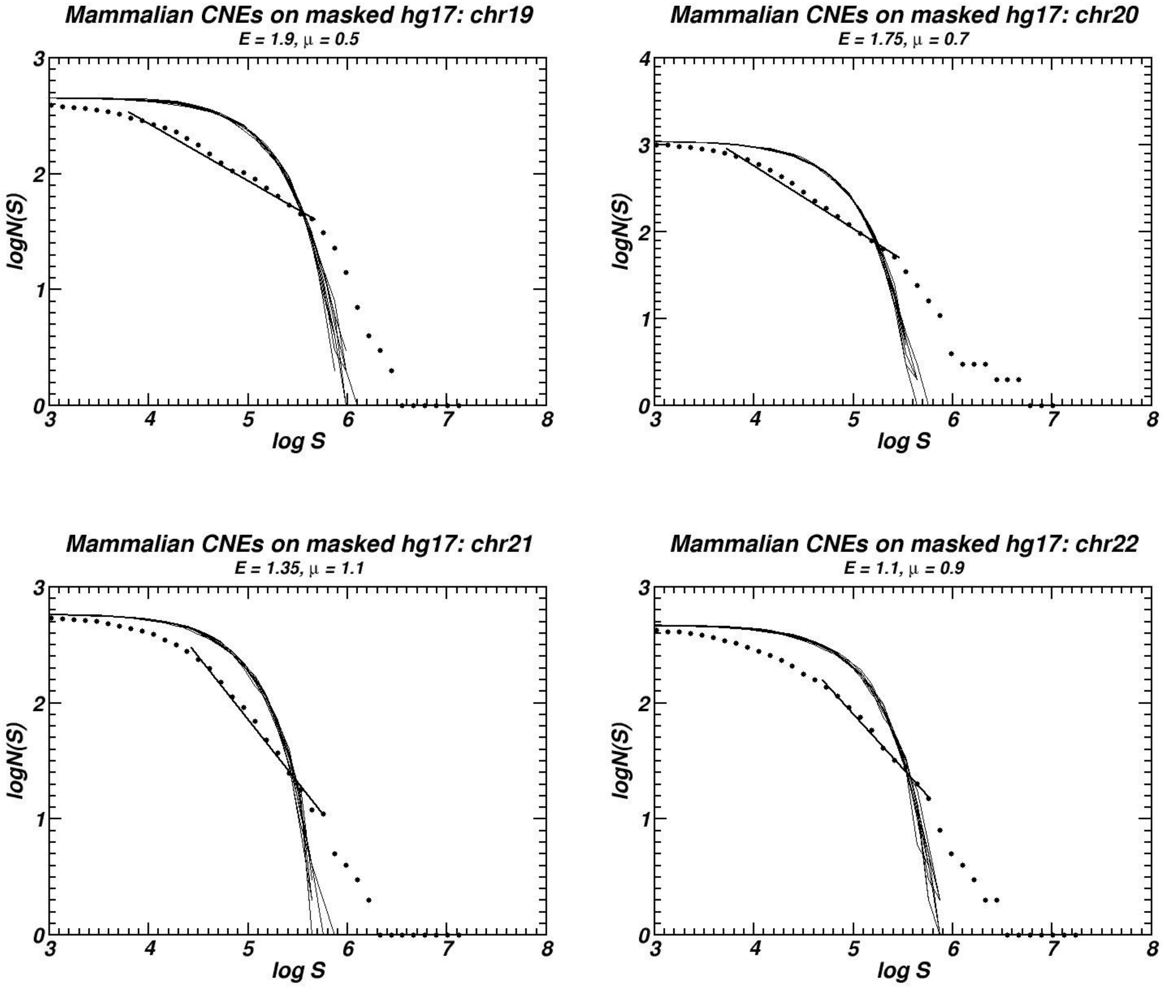
Mammalian CNEs on masked hg17: chr19
E = 1.9, μ = 0.5
Mammalian CNEs on masked hg17: chr20
E = 1.75, μ = 0.7
Mammalian CNEs on masked hg17: chr21
E = 1.35, μ = 1.1
Mammalian CNEs on masked hg17: chr22
E = 1.1, μ = 0.9
logN(S)
log S

EU100+ CNEs (Dataset i)

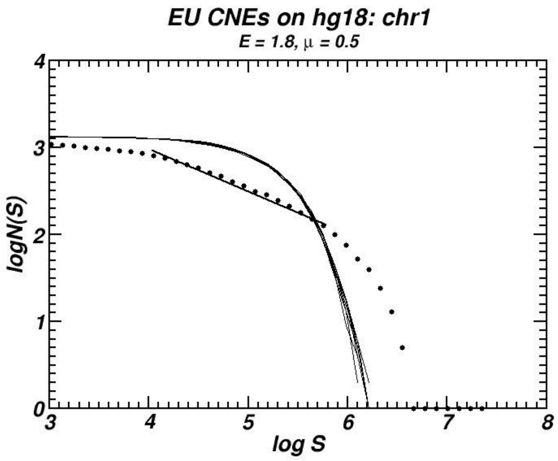

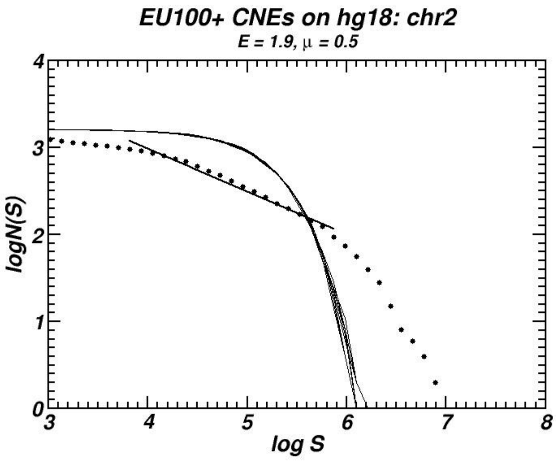

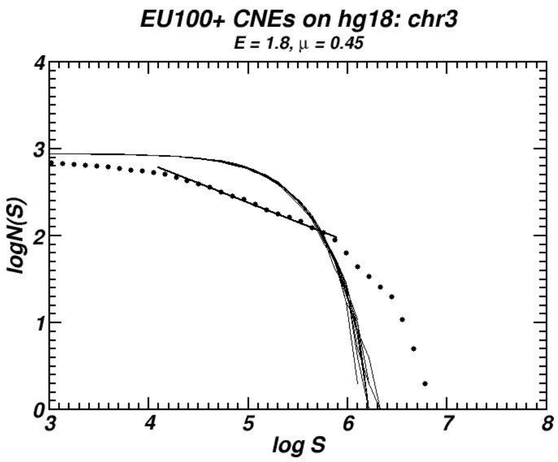

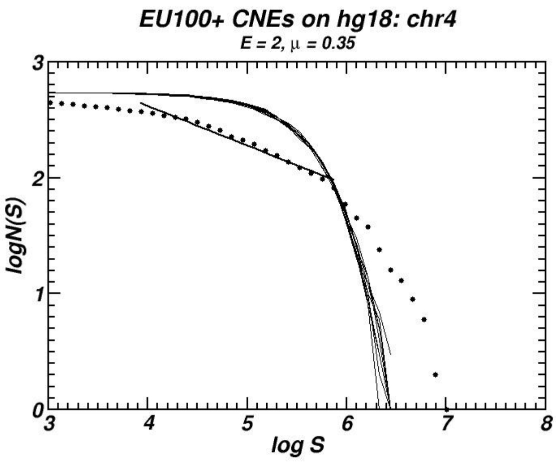

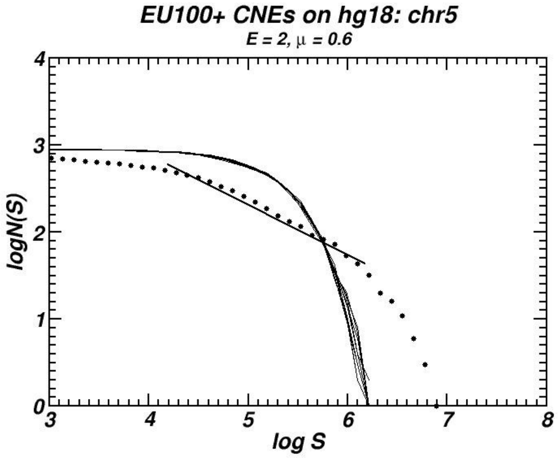

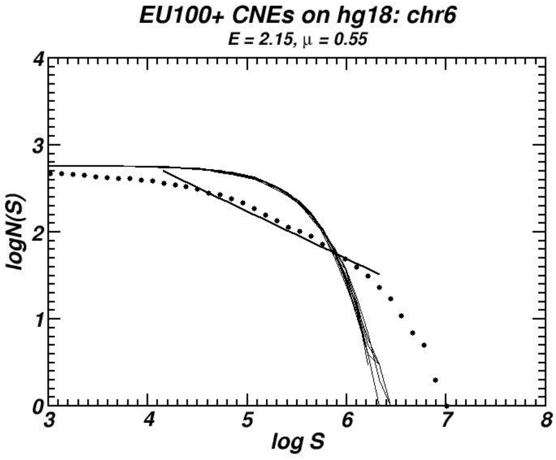

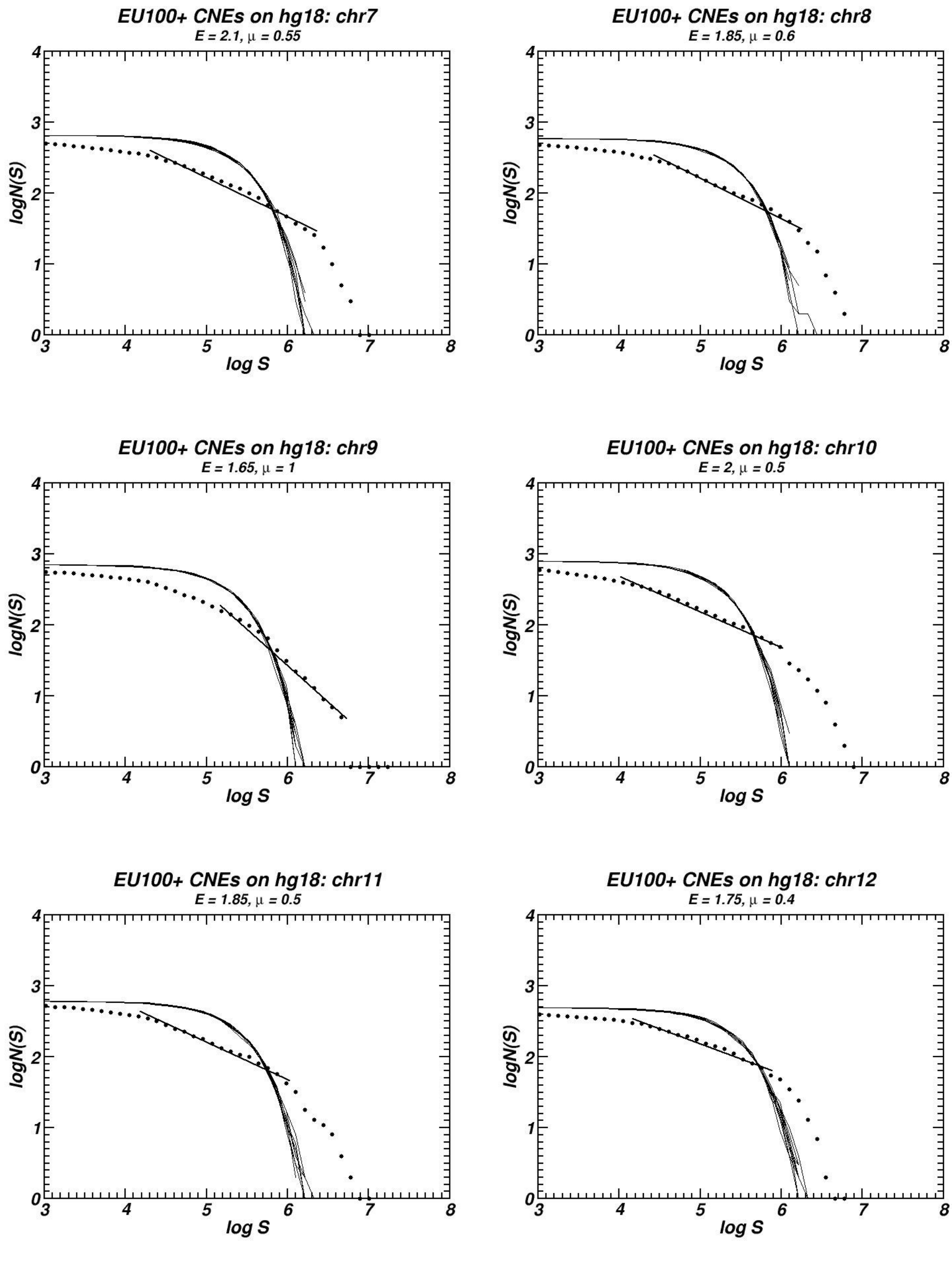
EU100+ CNEs on hg18: chr7
E = 2.1, μ = 0.55
EU100+ CNEs on hg18: chr8
E = 1.85, μ = 0.6
EU100+ CNEs on hg18: chr9
E = 1.65, μ = 1
EU100+ CNEs on hg18: chr10
E = 2, μ = 0.5
EU100+ CNEs on hg18: chr11
E = 1.85, μ = 0.5
EU100+ CNEs on hg18: chr12
E = 1.75, μ = 0.4
logN(S)
log S

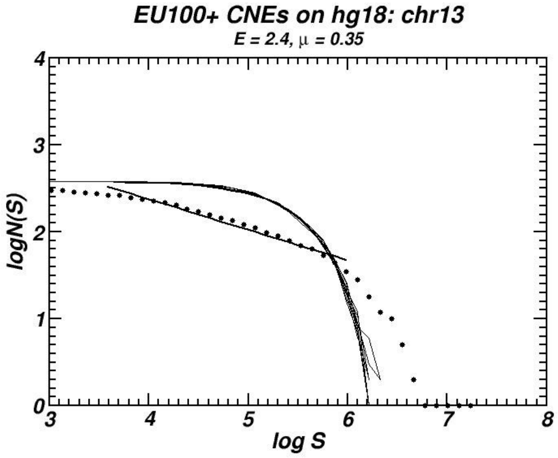
EU100+ CNEs on hg18: chr13
E = 2.4, μ = 0.35
logN(S)
log S

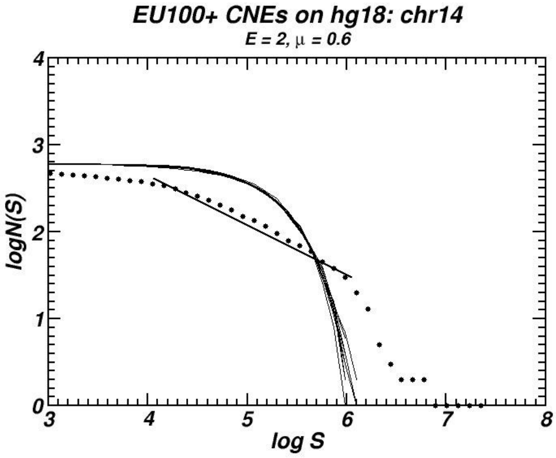
EU100+ CNEs on hg18: chr14
E = 2, μ = 0.6
logN(S)
log S

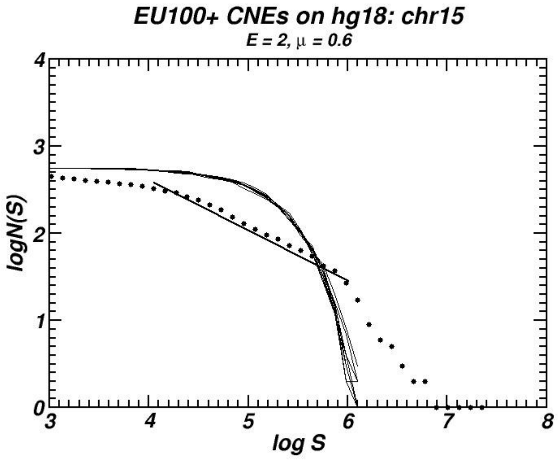
EU100+ CNEs on hg18: chr15
E = 2, μ = 0.6
logN(S)
log S

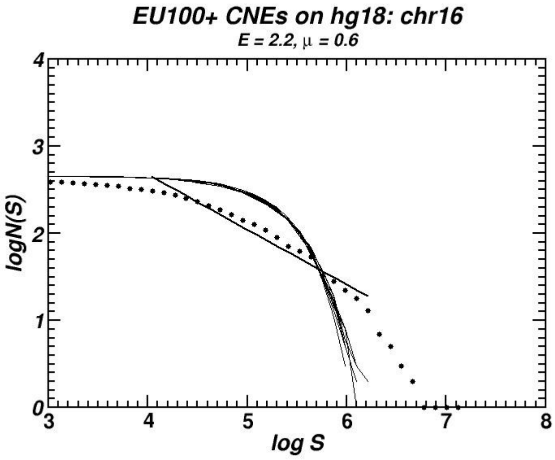
EU100+ CNEs on hg18: chr16
E = 2.2, μ = 0.6
logN(S)
log S

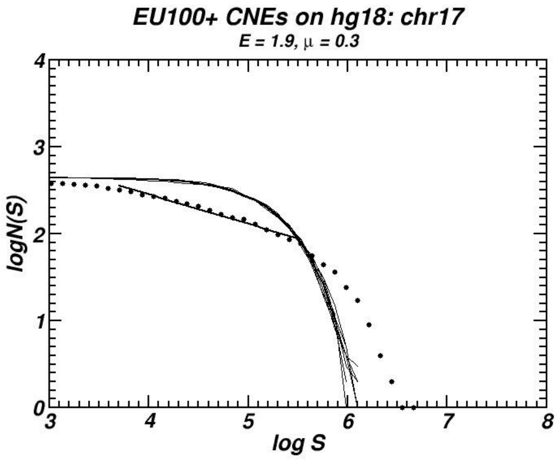
EU100+ CNEs on hg18: chr17
E = 1.9, μ = 0.3
logN(S)
log S

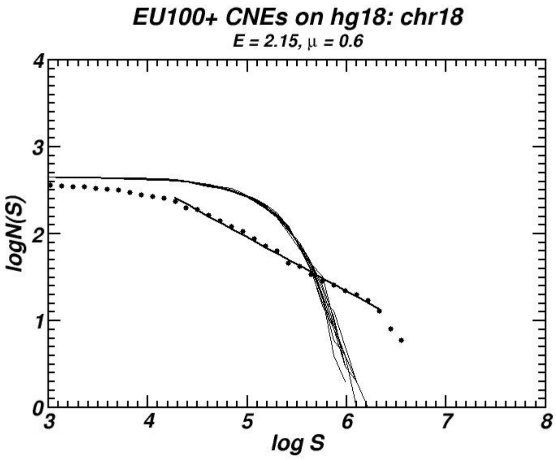
EU100+ CNEs on hg18: chr18
E = 2.15, μ = 0.6
logN(S)
log S

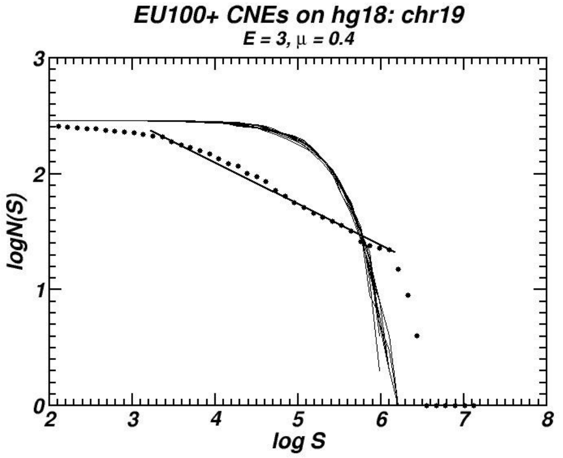
EU100+ CNEs on hg18: chr19
E = 3, μ = 0.4
logN(S)
log S

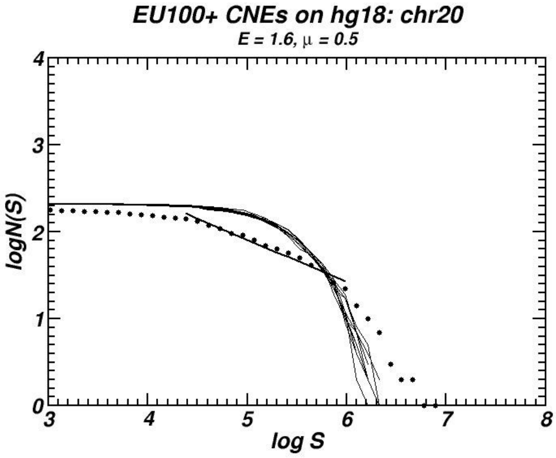
EU100+ CNEs on hg18: chr20
E = 1.6, μ = 0.5
logN(S)
log S

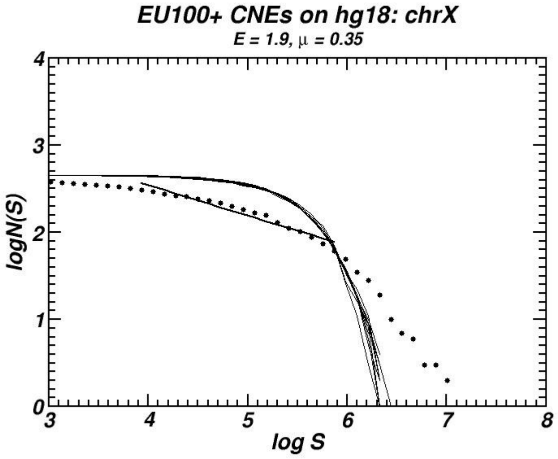
EU100+ CNEs on hg18: chrX
E = 1.9, μ = 0.35
logN(S)
log S

EU100+ CNEs on gene-masked genome

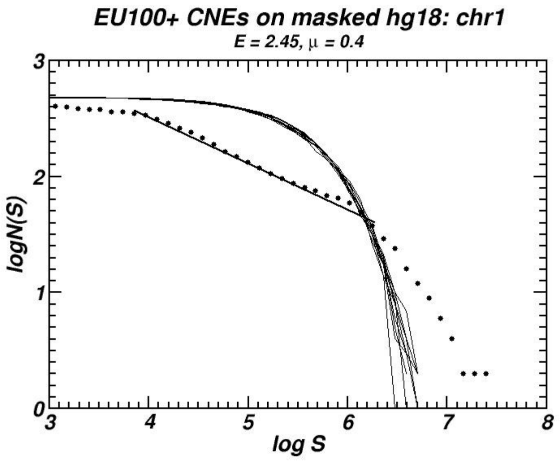

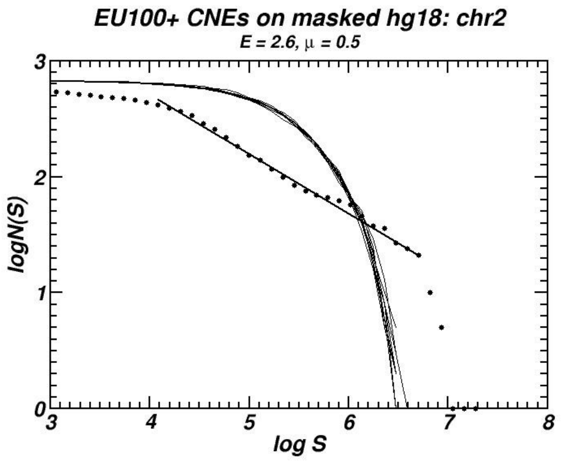

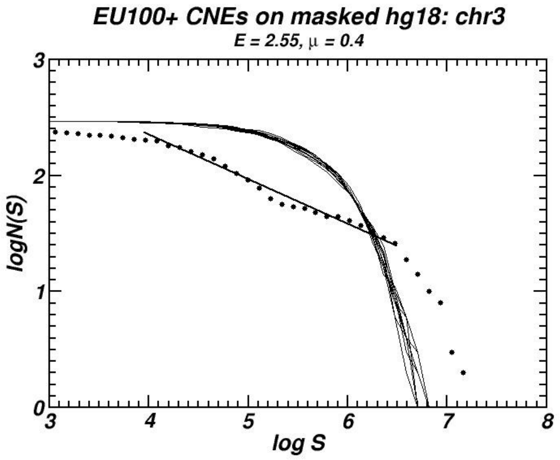

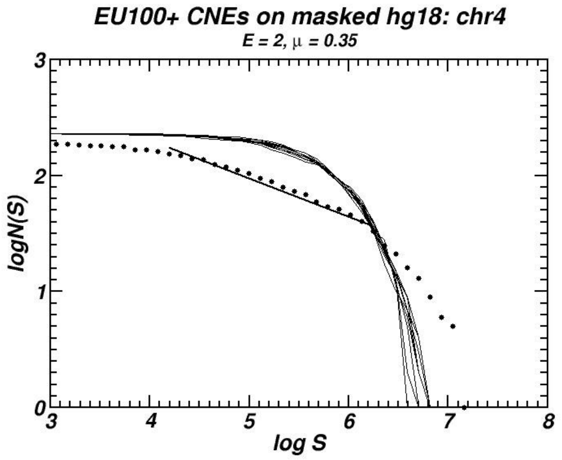

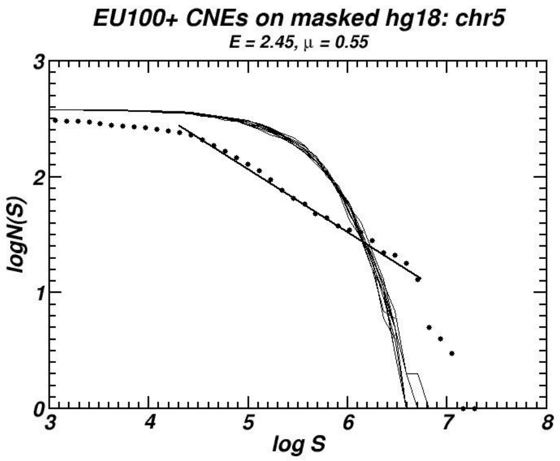

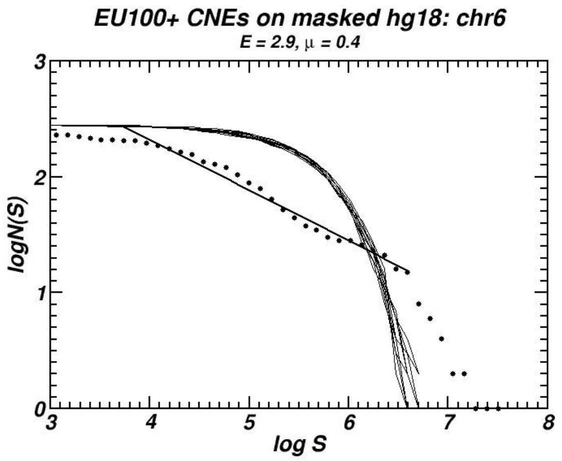

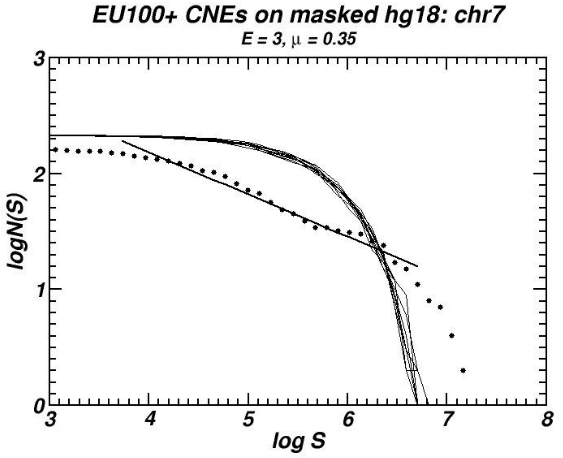
EU100+ CNEs on masked hg18: chr7
E = 3, μ = 0.35
logN(S)
log S
0 1 2 3
3 4 5 6 7 8

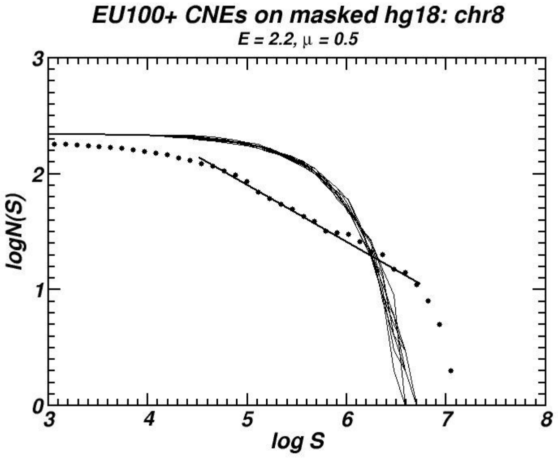
EU100+ CNEs on masked hg18: chr8
E = 2.2, μ = 0.5
logN(S)
log S
0 1 2 3
3 4 5 6 7 8

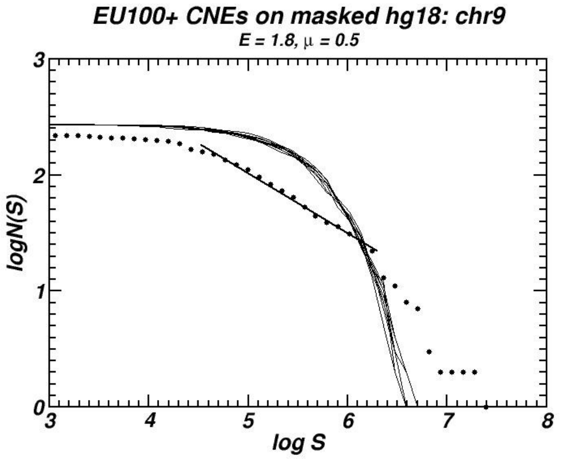
EU100+ CNEs on masked hg18: chr9
E = 1.8, μ = 0.5
logN(S)
log S
0 1 2 3
3 4 5 6 7 8

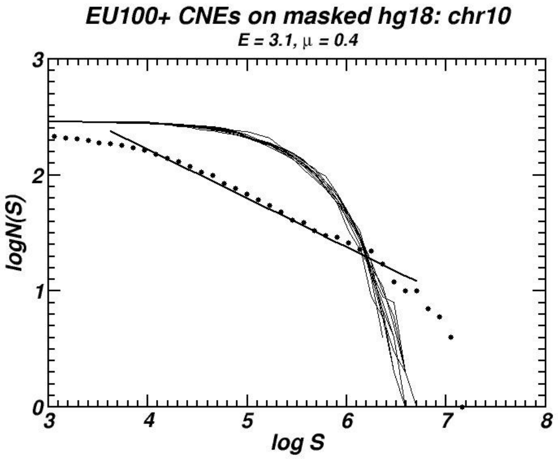
EU100+ CNEs on masked hg18: chr10
E = 3.1, μ = 0.4
logN(S)
log S
0 1 2 3
3 4 5 6 7 8

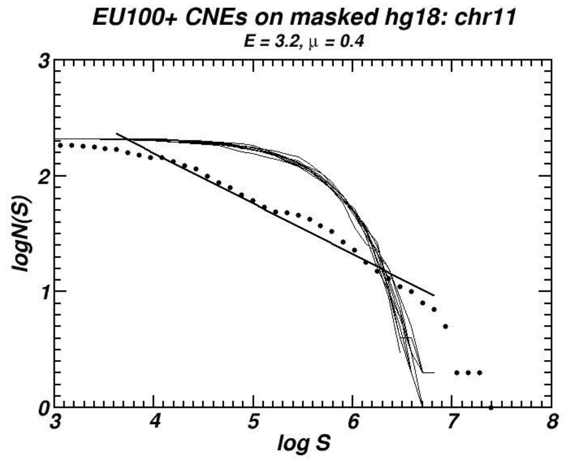
EU100+ CNEs on masked hg18: chr11
E = 3.2, μ = 0.4
logN(S)
log S
0 1 2 3
3 4 5 6 7 8

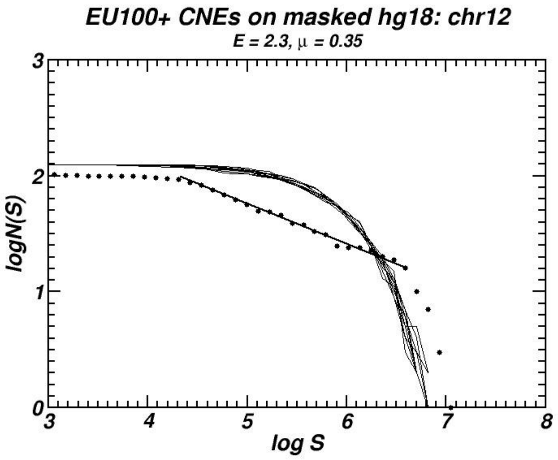
EU100+ CNEs on masked hg18: chr12
E = 2.3, μ = 0.35
logN(S)
log S
0 1 2 3
3 4 5 6 7 8

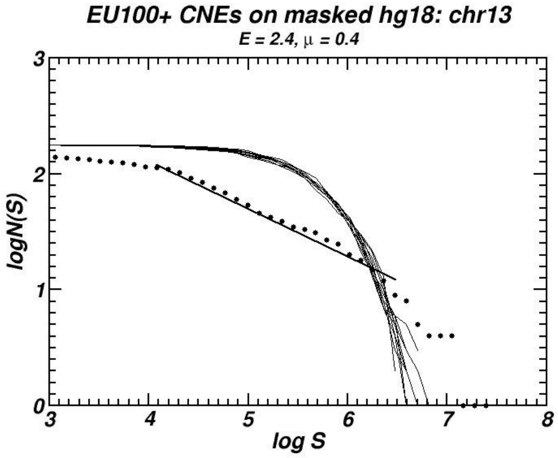
EU100+ CNEs on masked hg18: chr13
E = 2.4, μ = 0.4
logN(S)
log S

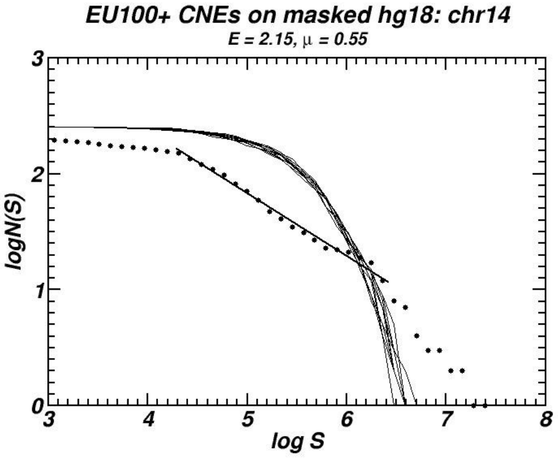
EU100+ CNEs on masked hg18: chr14
E = 2.15, μ = 0.55
logN(S)
log S

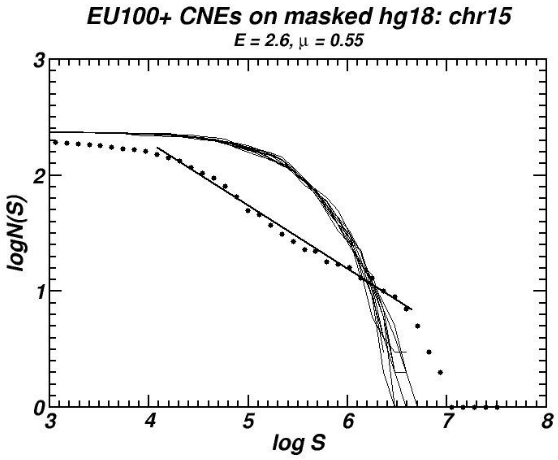
EU100+ CNEs on masked hg18: chr15
E = 2.6, μ = 0.55
logN(S)
log S

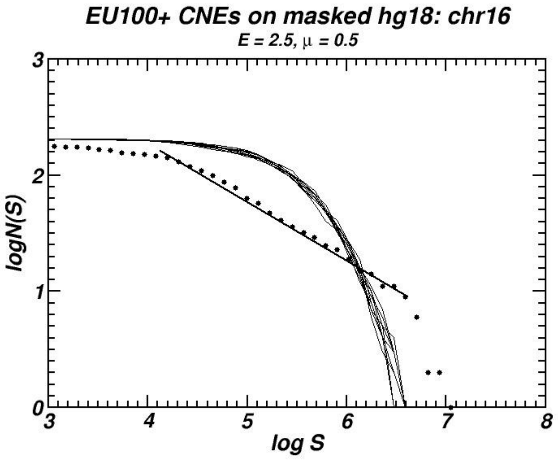
EU100+ CNEs on masked hg18: chr16
E = 2.5, μ = 0.5
logN(S)
log S

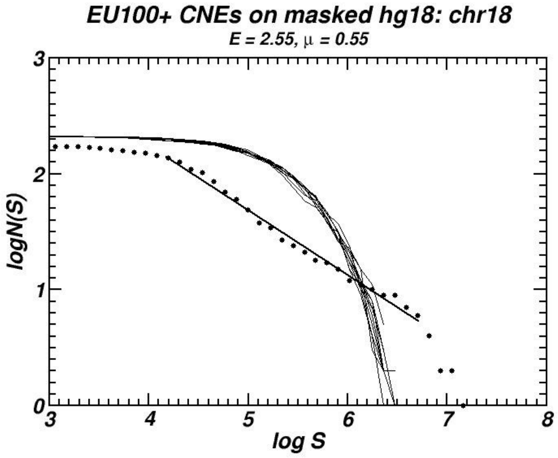
EU100+ CNEs on masked hg18: chr18
E = 2.55, μ = 0.55
logN(S)
log S

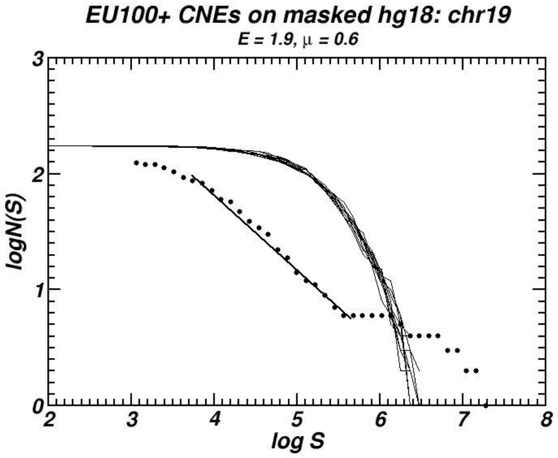
EU100+ CNEs on masked hg18: chr19
E = 1.9, μ = 0.6
logN(S)
log S

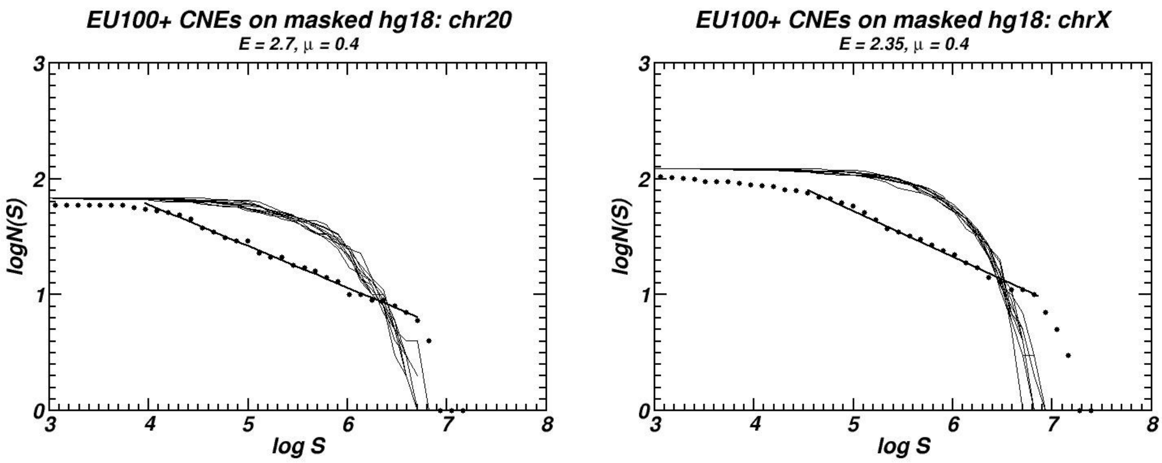
EU100+ CNEs on masked hg18: chr20
E = 2.7, μ = 0.4
logN(S)
log S
EU100+ CNEs on masked hg18: chrX
E = 2.35, μ = 0.4
logN(S)
log S

Worm CNEs (Dataset ix)

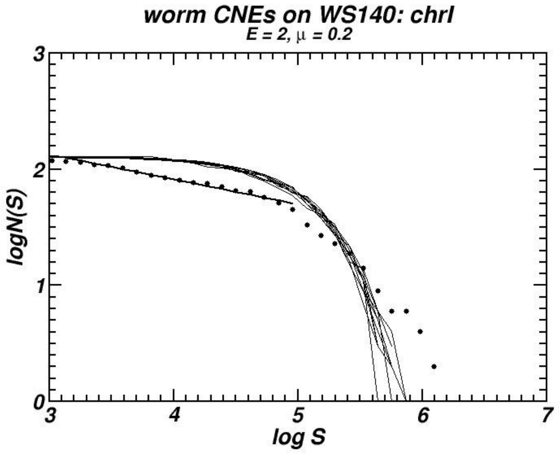

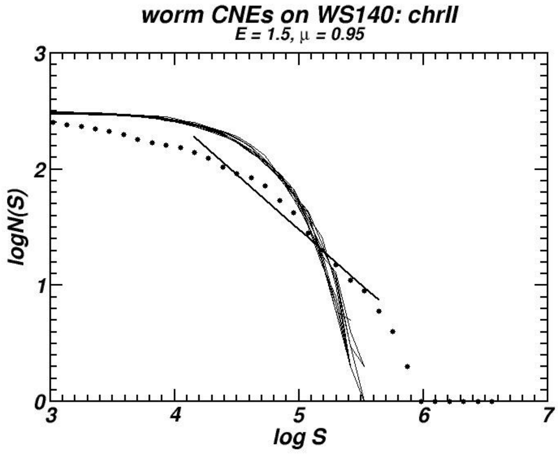

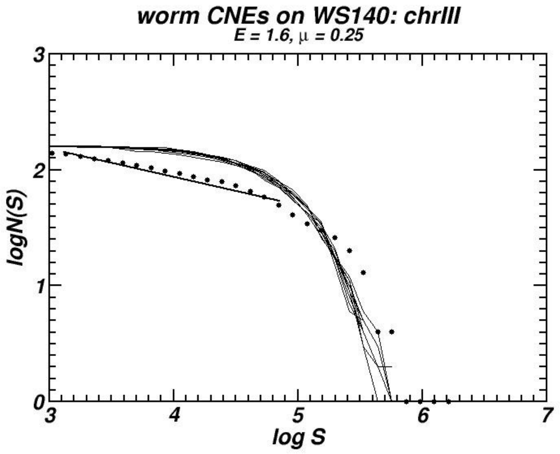

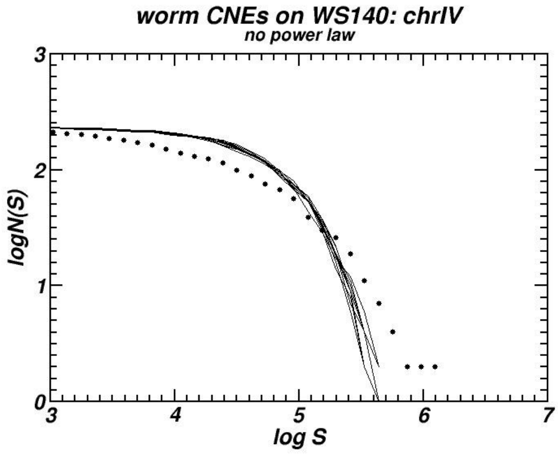

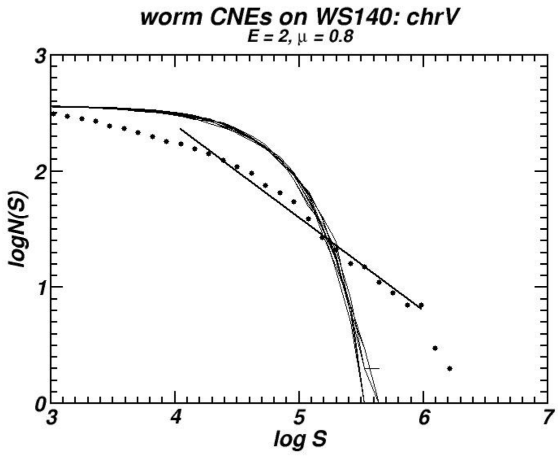

Human-Zebrafish CNEs (Dataset v)

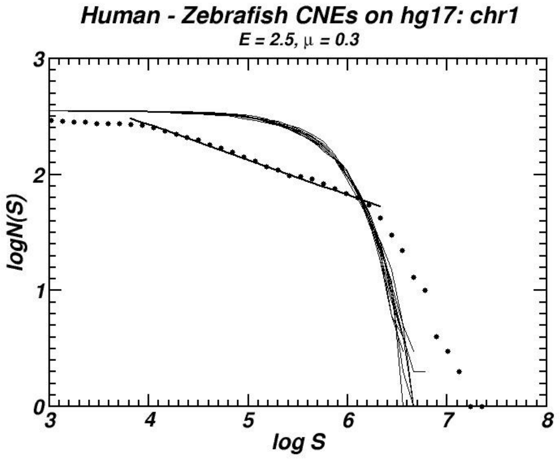

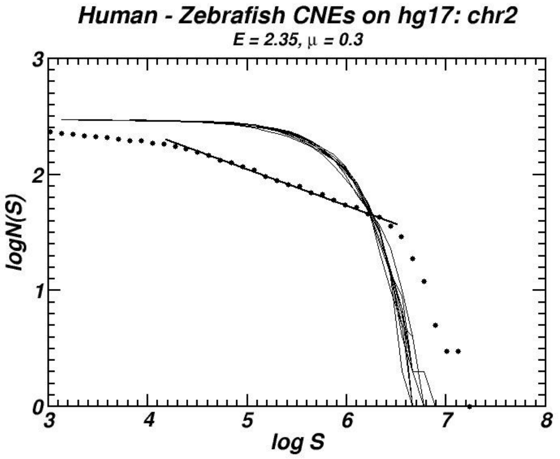

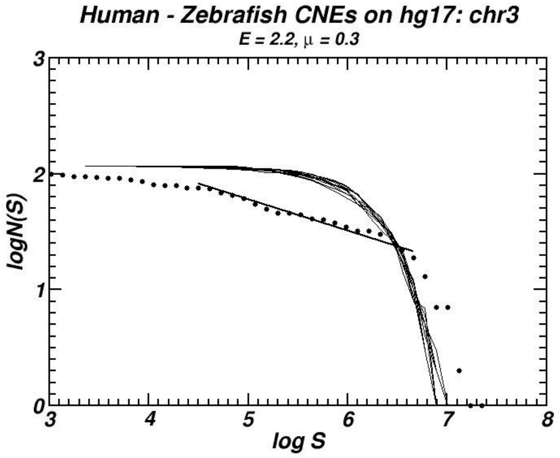

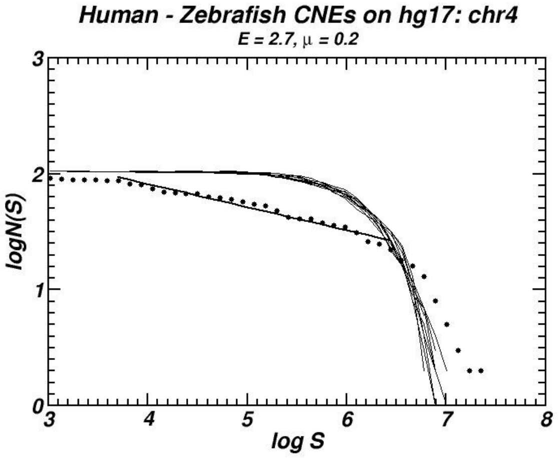

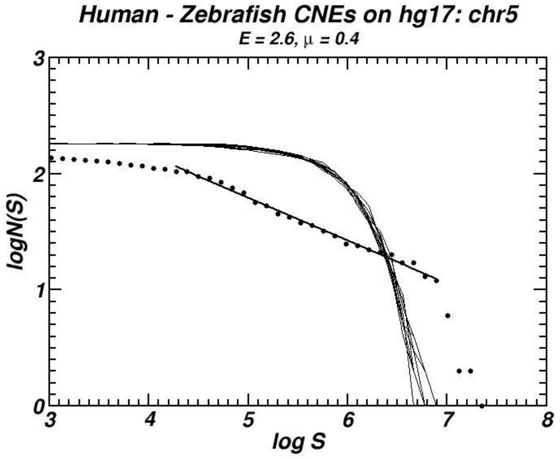

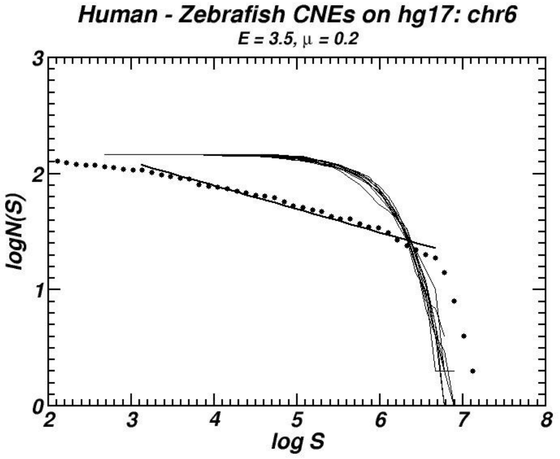

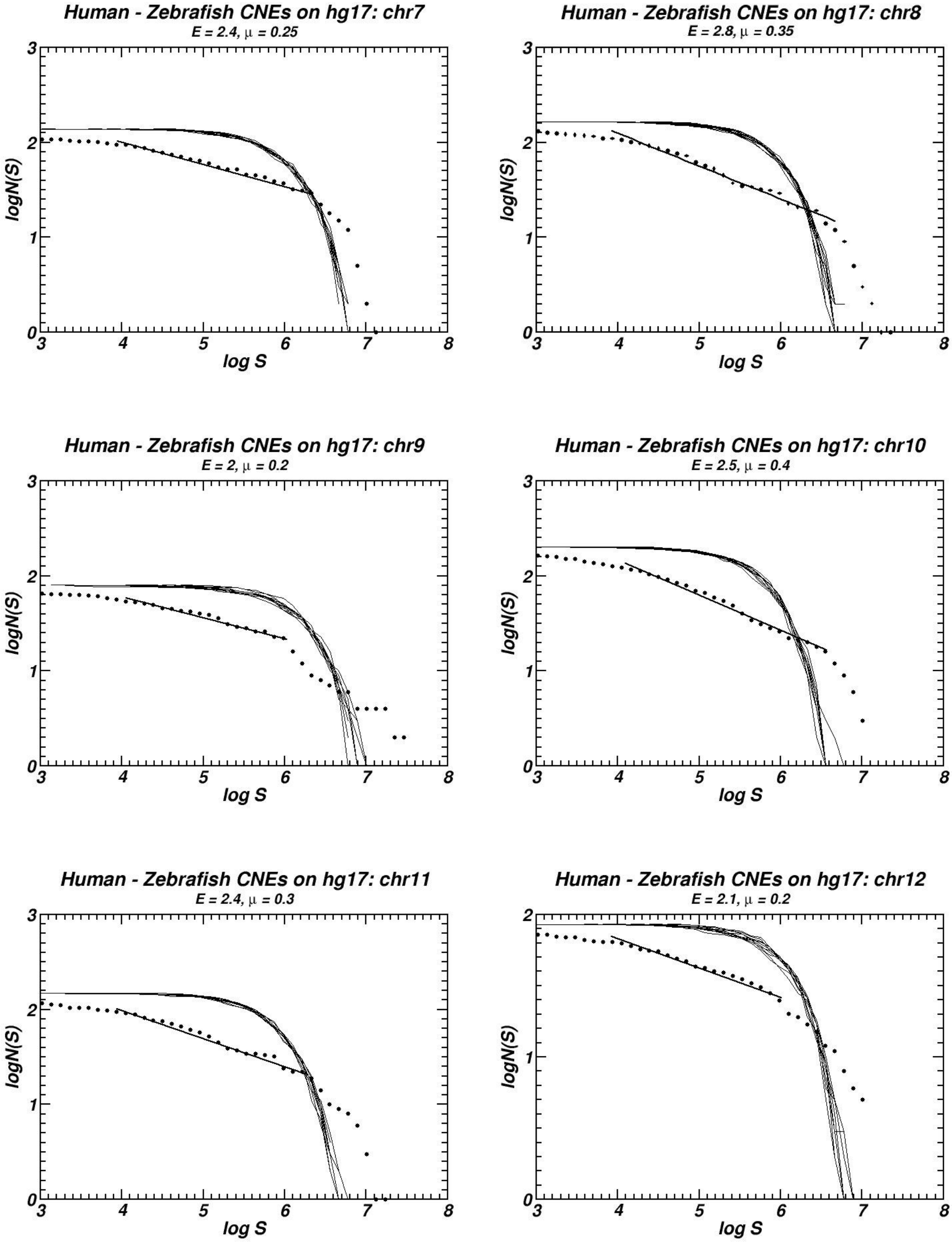

Human - Zebrafish CNEs on hg17: chr7
E = 2.4, μ = 0.25
Human - Zebrafish CNEs on hg17: chr8
E = 2.8, μ = 0.35
Human - Zebrafish CNEs on hg17: chr9
E = 2, μ = 0.2
Human - Zebrafish CNEs on hg17: chr10
E = 2.5, μ = 0.4
Human - Zebrafish CNEs on hg17: chr11
E = 2.4, μ = 0.3
Human - Zebrafish CNEs on hg17: chr12
E = 2.1, μ = 0.2
logN(S)
log S

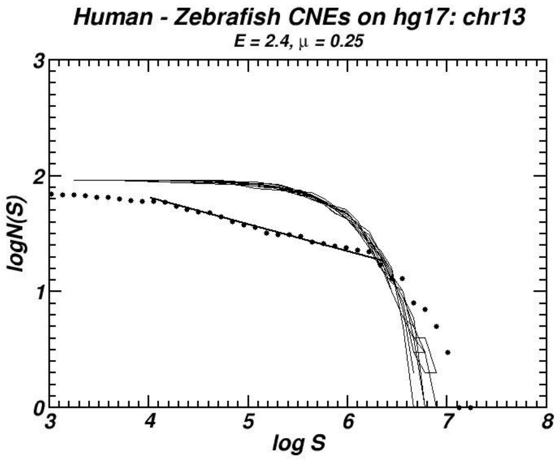
Human - Zebrafish CNEs on hg17: chr13
E = 2.4, μ = 0.25
logN(S)
log S

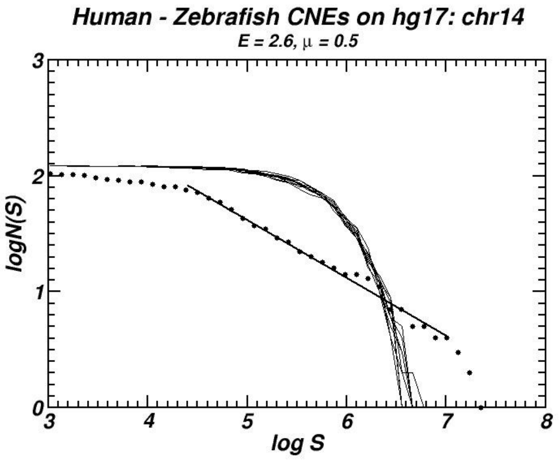
Human - Zebrafish CNEs on hg17: chr14
E = 2.6, μ = 0.5
logN(S)
log S

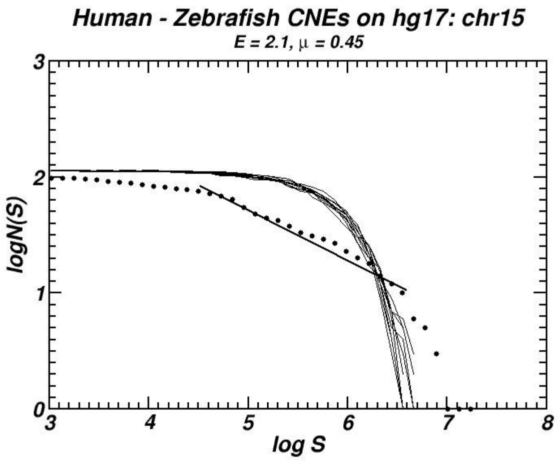
Human - Zebrafish CNEs on hg17: chr15
E = 2.1, μ = 0.45
logN(S)
log S

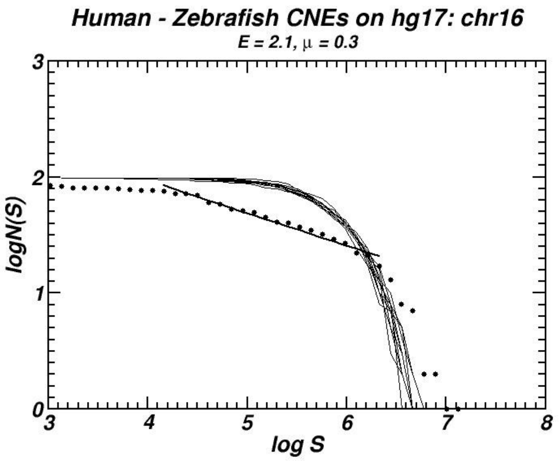
Human - Zebrafish CNEs on hg17: chr16
E = 2.1, μ = 0.3
logN(S)
log S

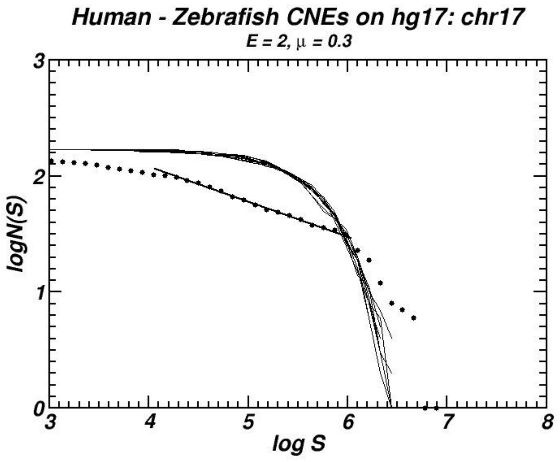
Human - Zebrafish CNEs on hg17: chr17
E = 2, μ = 0.3
logN(S)
log S

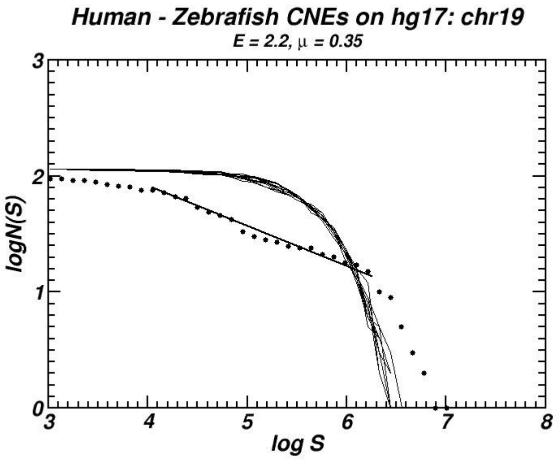
Human - Zebrafish CNEs on hg17: chr19
E = 2.2, μ = 0.35
logN(S)
log S

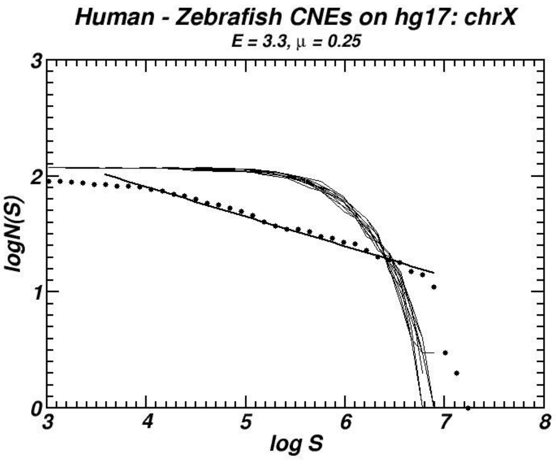

Human - Zebrafish CNEs on hg17: chrX
E = 3.3, μ = 0.25
logN(S)
log S
0
1
2
3
3
4
5
6
7
8

Human-Zebrafish CNEs on gene-masked genome

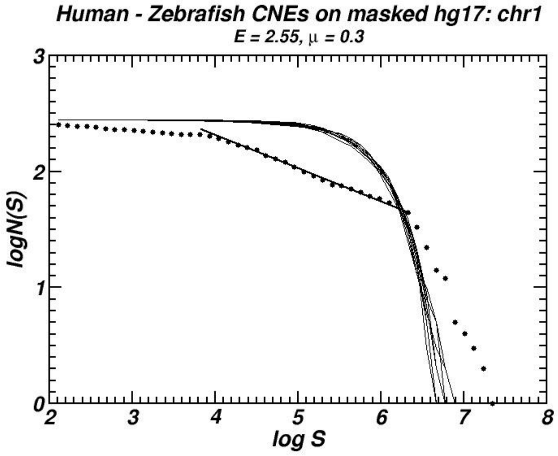

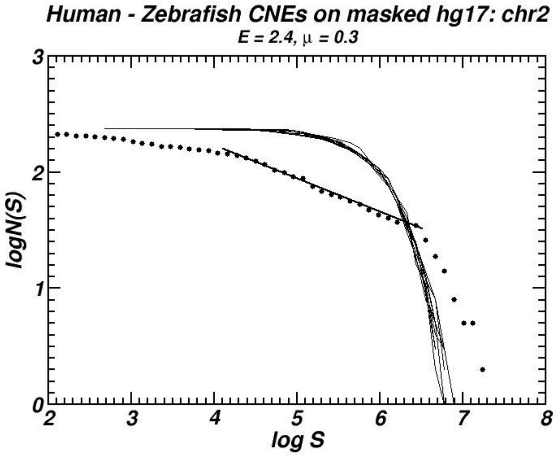

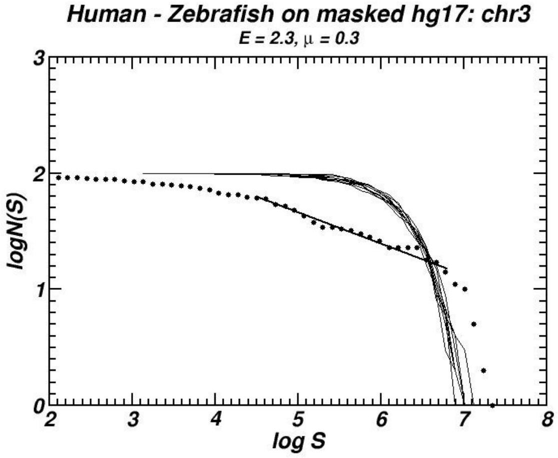

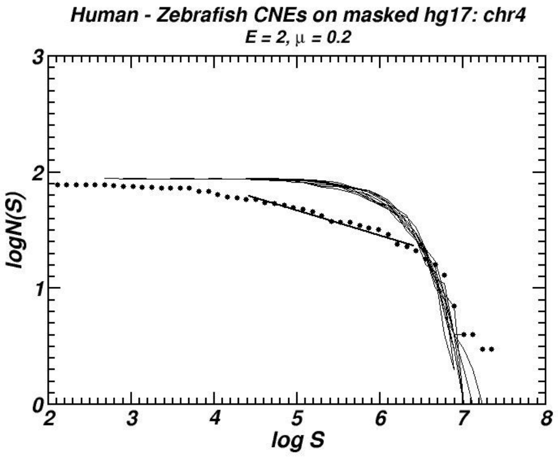

Human-Fugu CNEs (Dataset iv)

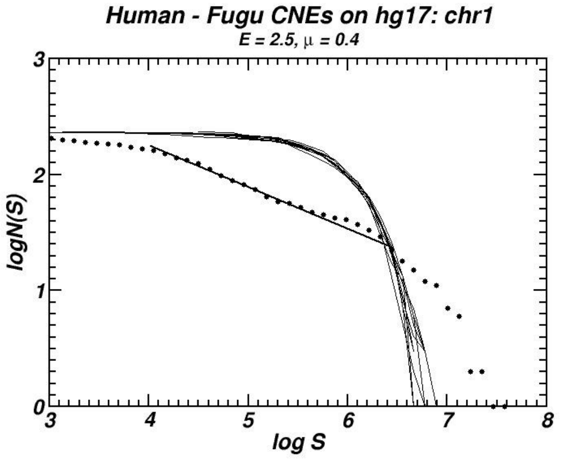

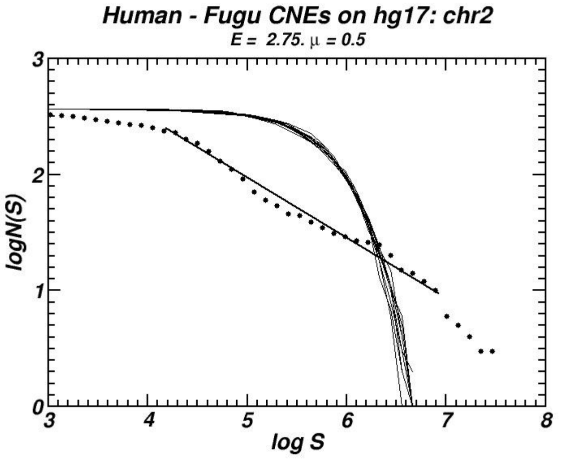

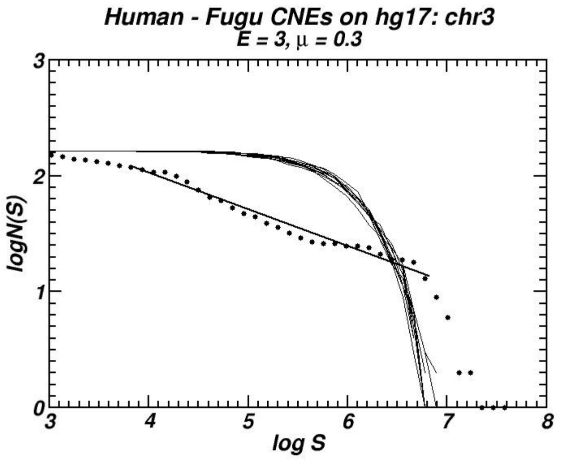

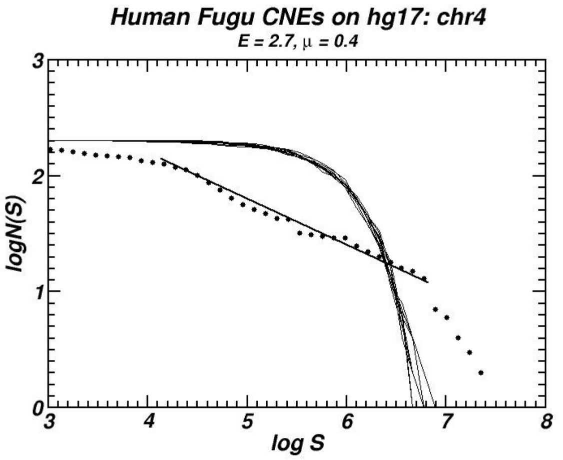

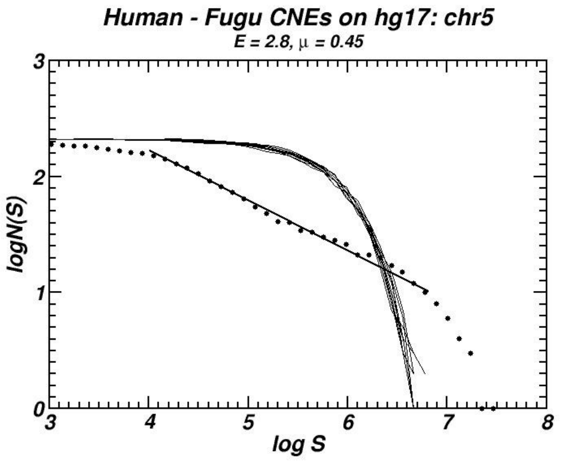

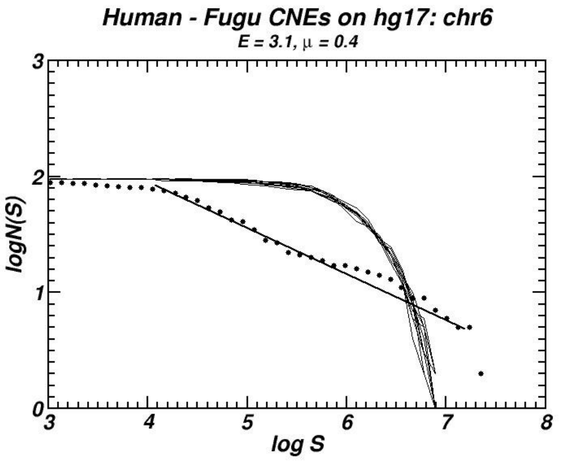

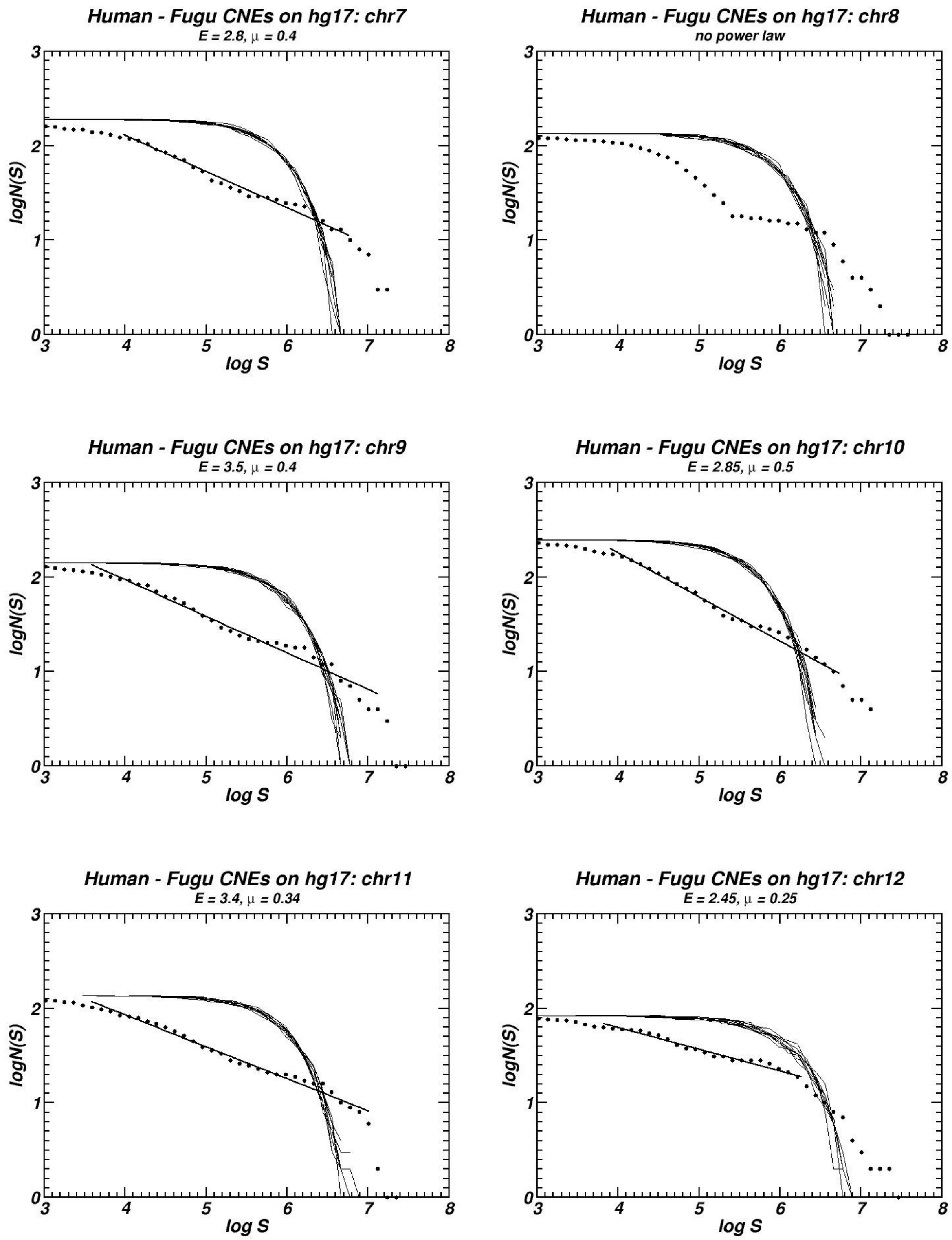
Human - Fugu CNEs on hg17: chr7
E = 2.8, μ = 0.4
Human - Fugu CNEs on hg17: chr8
no power law
Human - Fugu CNEs on hg17: chr9
E = 3.5, μ = 0.4
Human - Fugu CNEs on hg17: chr10
E = 2.85, μ = 0.5
Human - Fugu CNEs on hg17: chr11
E = 3.4, μ = 0.34
Human - Fugu CNEs on hg17: chr12
E = 2.45, μ = 0.25
logN(S)
log S

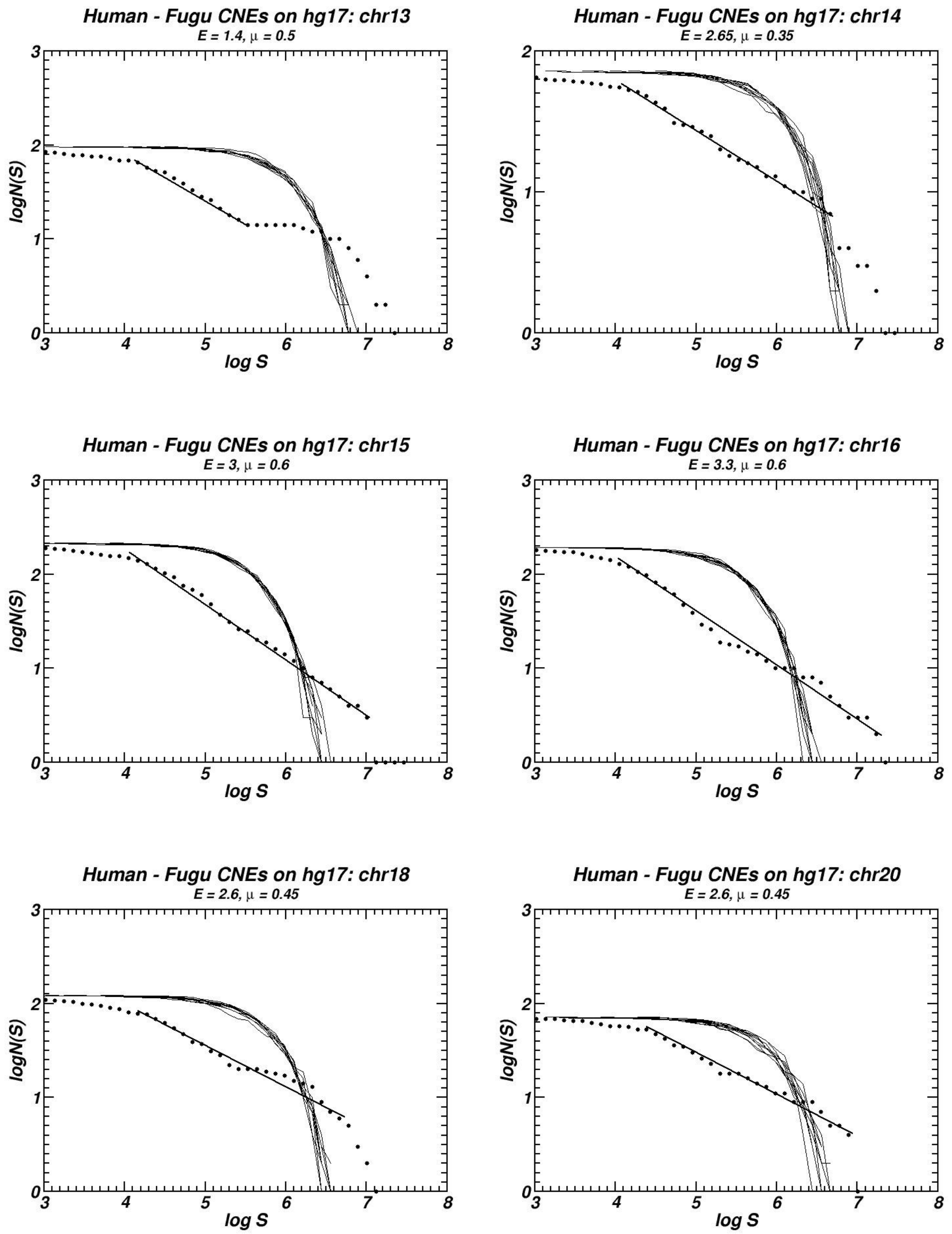
Human - Fugu CNEs on hg17: chr13
E = 1.4, μ = 0.5
logN(S)
log S
Human - Fugu CNEs on hg17: chr14
E = 2.65, μ = 0.35
logN(S)
log S
Human - Fugu CNEs on hg17: chr15
E = 3, μ = 0.6
logN(S)
log S
Human - Fugu CNEs on hg17: chr16
E = 3.3, μ = 0.6
logN(S)
log S
Human - Fugu CNEs on hg17: chr18
E = 2.6, μ = 0.45
logN(S)
log S
Human - Fugu CNEs on hg17: chr20
E = 2.6, μ = 0.45
logN(S)
log S

Human-Elephant Shark CNEs (Dataset vi)

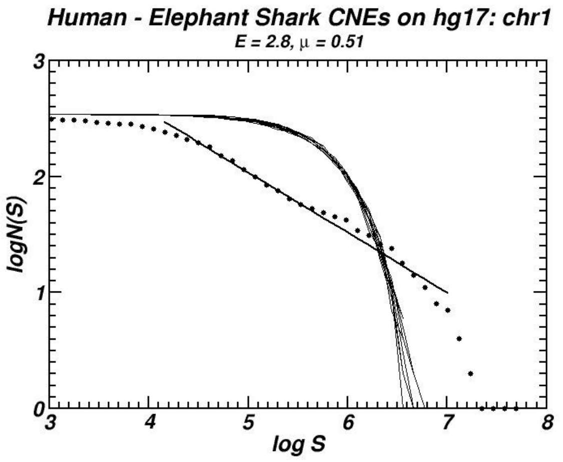

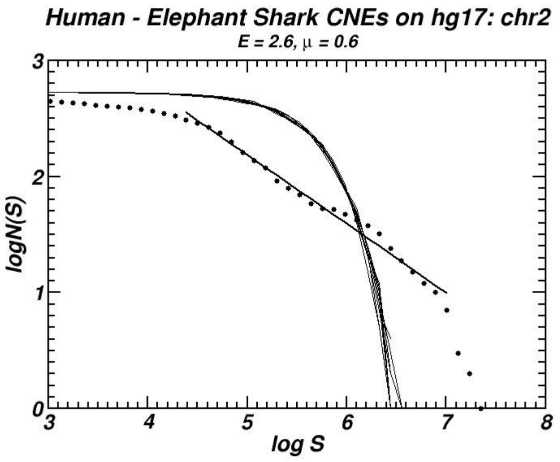

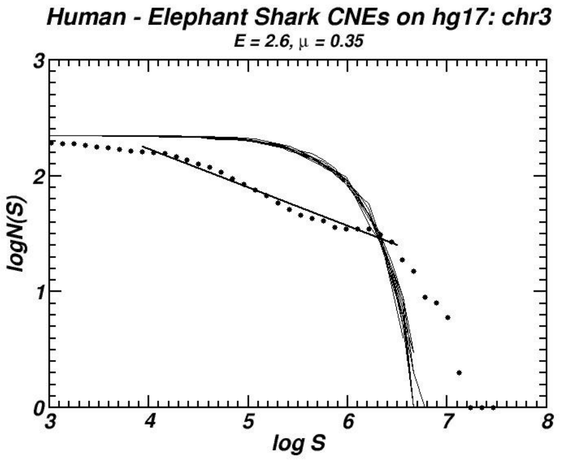

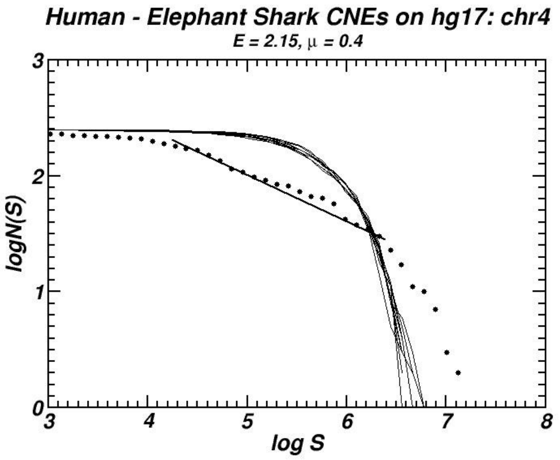

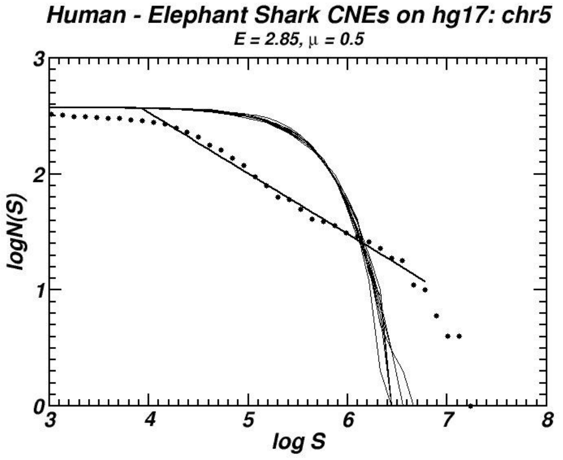

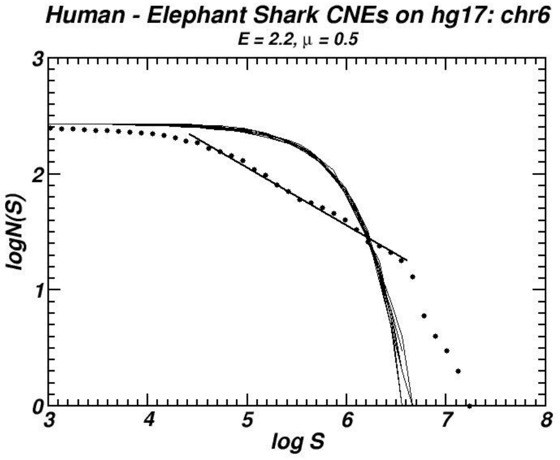

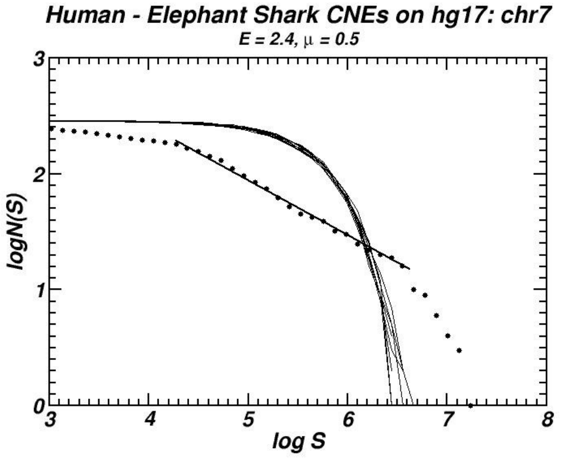
Human - Elephant Shark CNEs on hg17: chr7
E = 2.4, μ = 0.5
logN(S)
log S

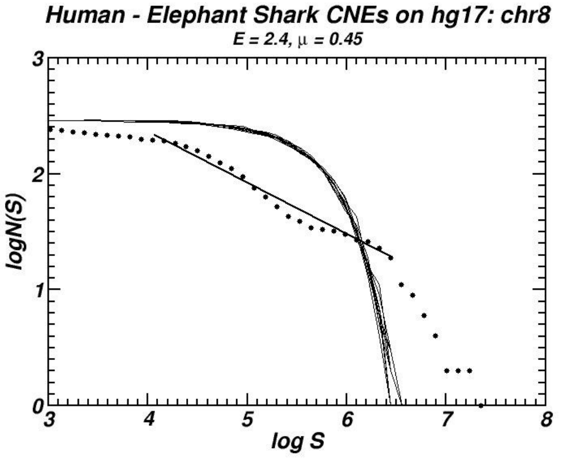
Human - Elephant Shark CNEs on hg17: chr8
E = 2.4, μ = 0.45
logN(S)
log S

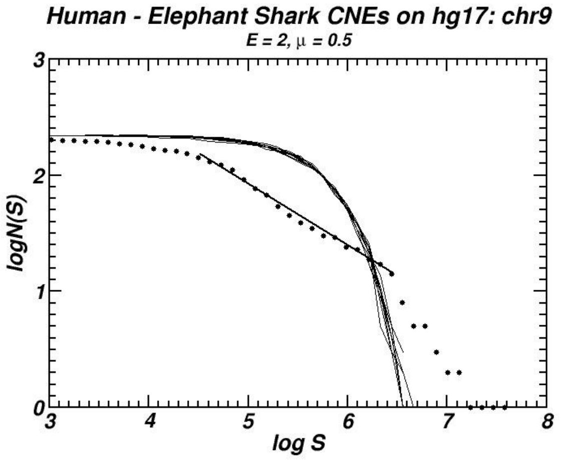
Human - Elephant Shark CNEs on hg17: chr9
E = 2, μ = 0.5
logN(S)
log S

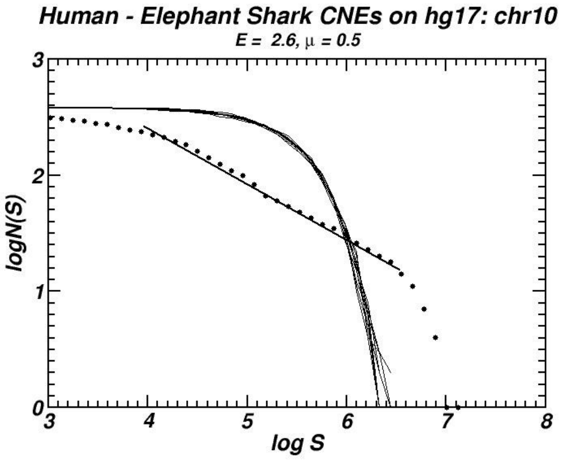
Human - Elephant Shark CNEs on hg17: chr10
E = 2.6, μ = 0.5
logN(S)
log S

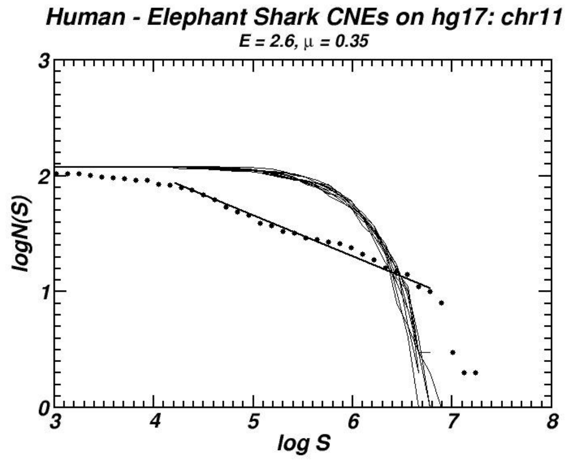
Human - Elephant Shark CNEs on hg17: chr11
E = 2.6, μ = 0.35
logN(S)
log S

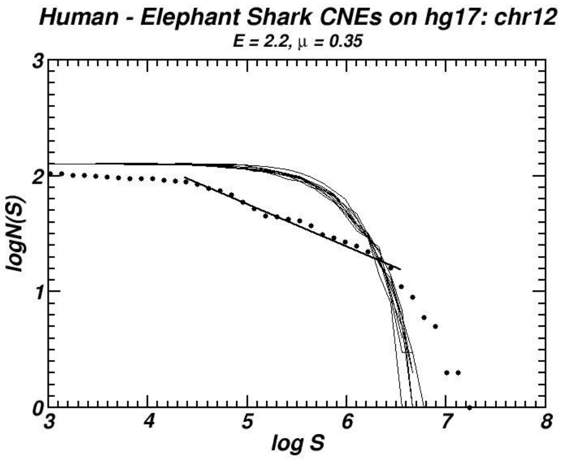
Human - Elephant Shark CNEs on hg17: chr12
E = 2.2, μ = 0.35
logN(S)
log S

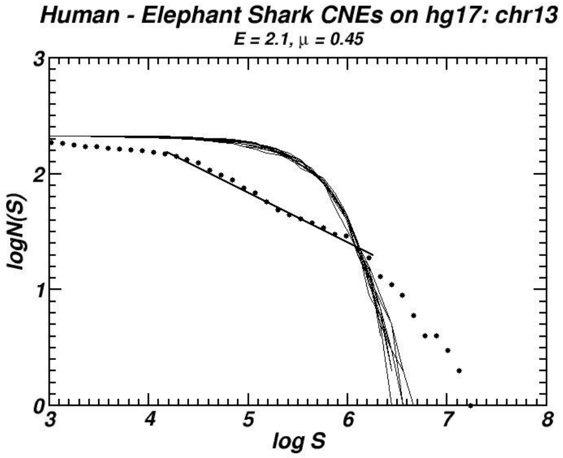
Human - Elephant Shark CNEs on hg17: chr13
E = 2.1, μ = 0.45
logN(S)
log S

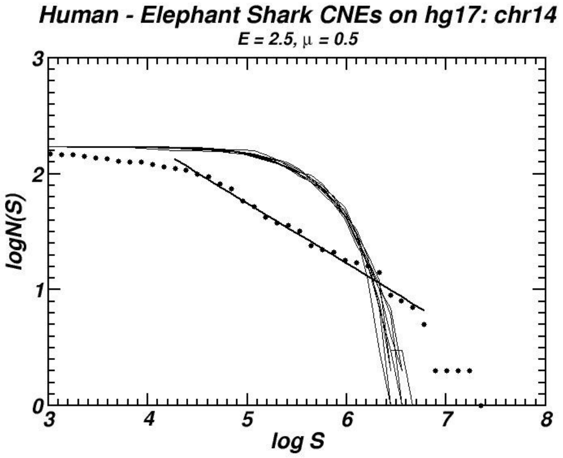
Human - Elephant Shark CNEs on hg17: chr14
E = 2.5, μ = 0.5
logN(S)
log S

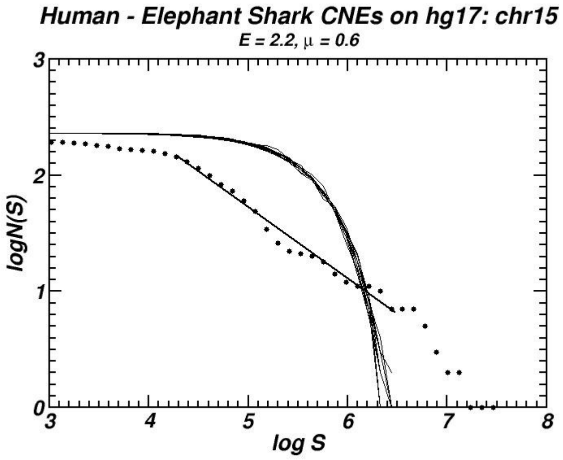
Human - Elephant Shark CNEs on hg17: chr15
E = 2.2, μ = 0.6
logN(S)
log S

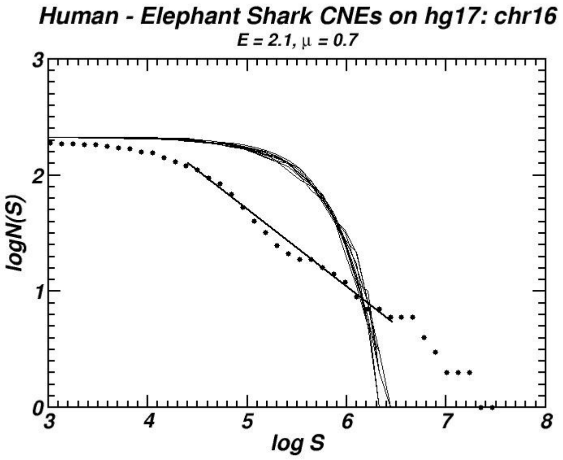
Human - Elephant Shark CNEs on hg17: chr16
E = 2.1, μ = 0.7
logN(S)
log S

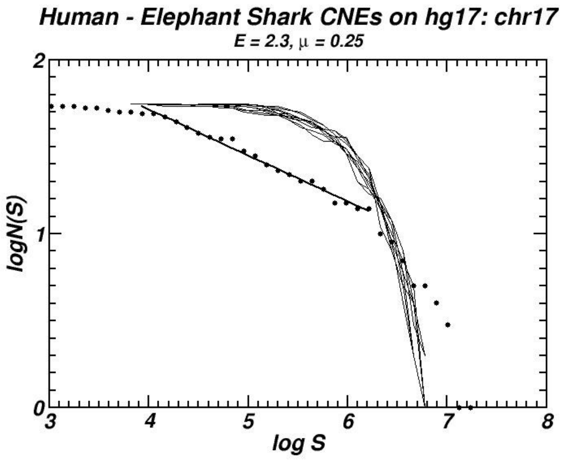
Human - Elephant Shark CNEs on hg17: chr17
E = 2.3, μ = 0.25
logN(S)
log S

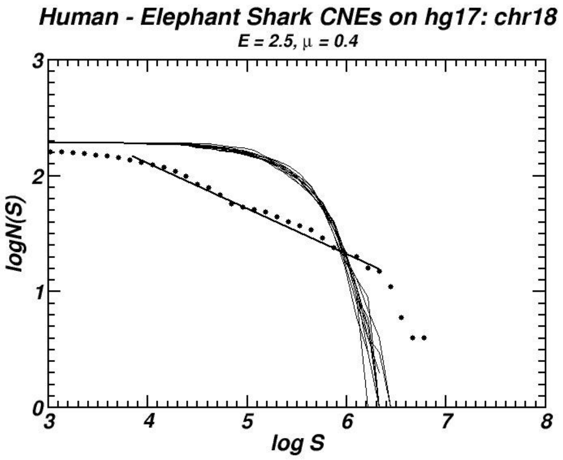
Human - Elephant Shark CNEs on hg17: chr18
E = 2.5, μ = 0.4
logN(S)
log S

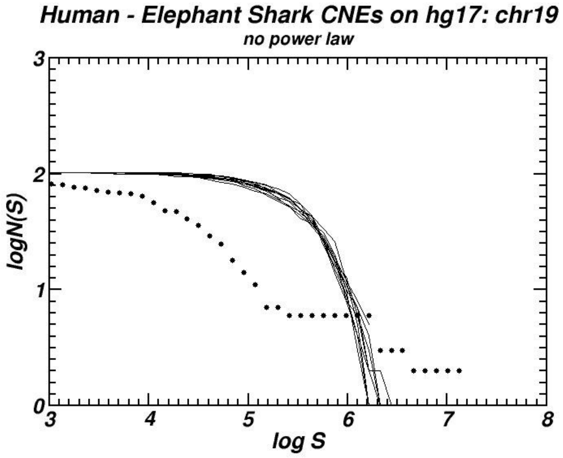
Human - Elephant Shark CNEs on hg17: chr19
no power law
logN(S)
log S

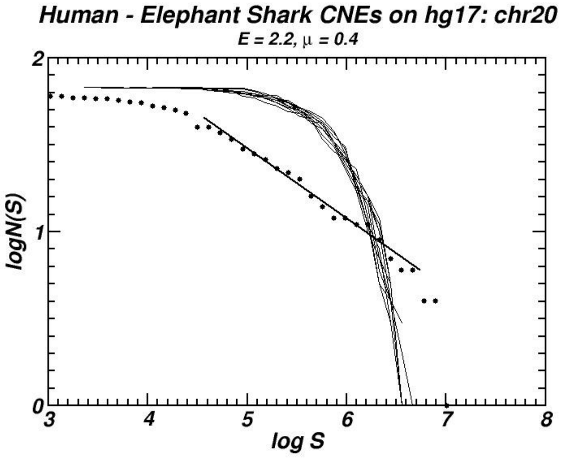
Human - Elephant Shark CNEs on hg17: chr20
E = 2.2, μ = 0.4
logN(S)
log S

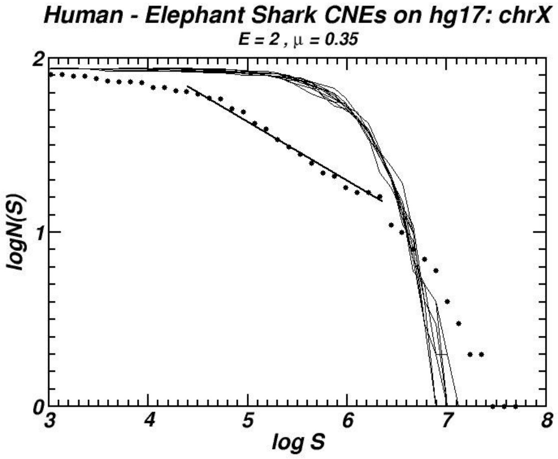
Human - Elephant Shark CNEs on hg17: chrX
E = 2 , μ = 0.35
logN(S)
log S

Human-Elephant Shark CNEs on gene-masked genome

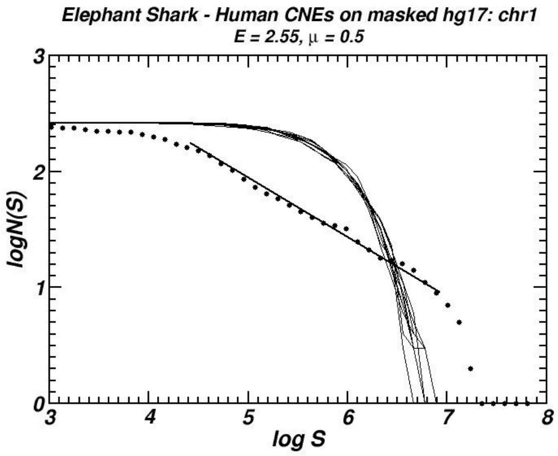

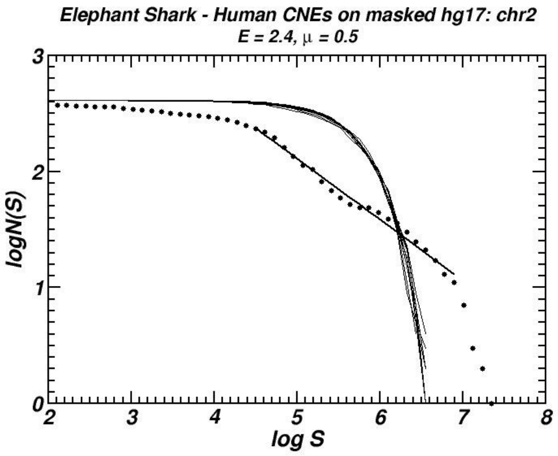

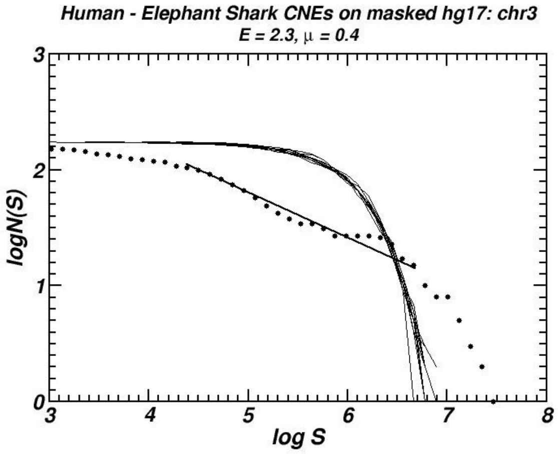

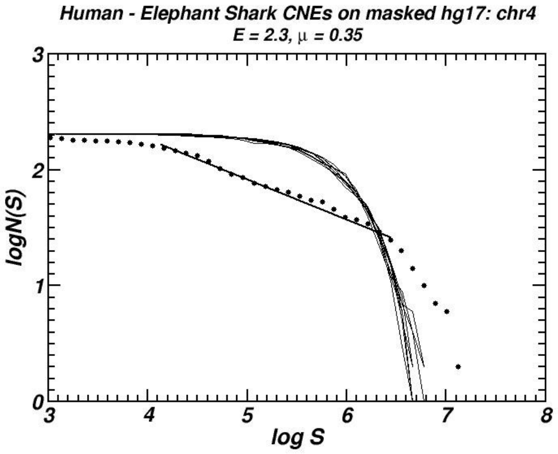

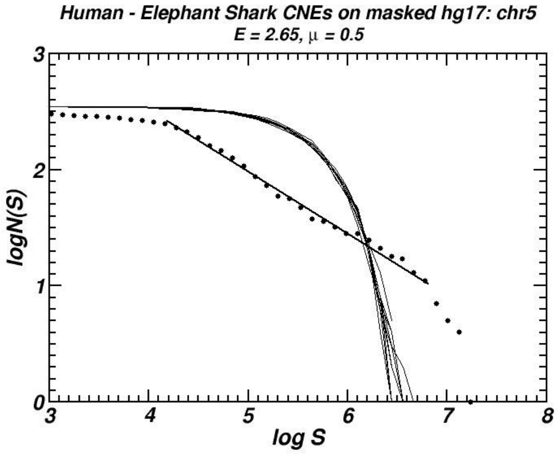

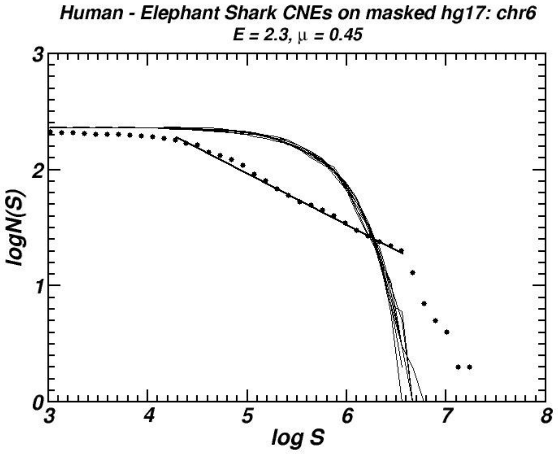

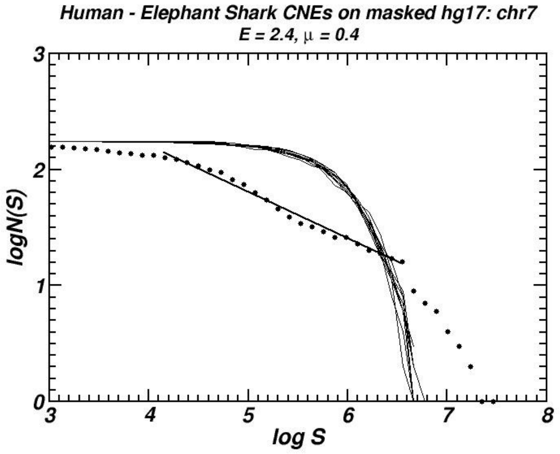
Human - Elephant Shark CNEs on masked hg17: chr7
E = 2.4, μ = 0.4
logN(S)
log S

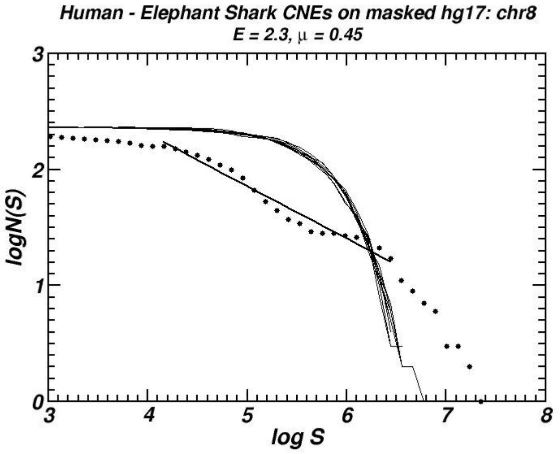
Human - Elephant Shark CNEs on masked hg17: chr8
E = 2.3, μ = 0.45
logN(S)
log S

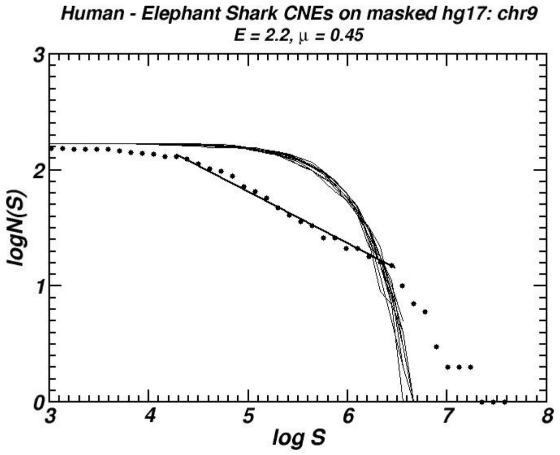
Human - Elephant Shark CNEs on masked hg17: chr9
E = 2.2, μ = 0.45
logN(S)
log S

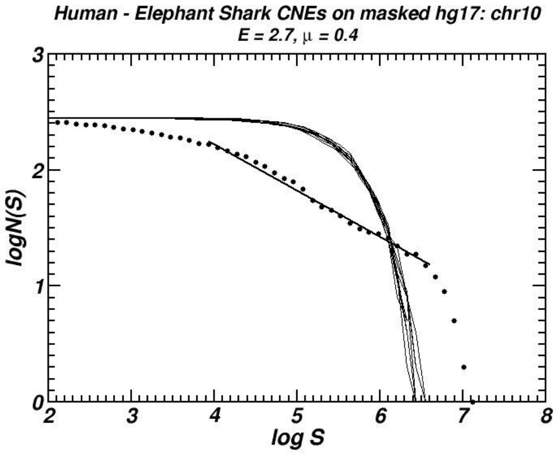
Human - Elephant Shark CNEs on masked hg17: chr10
E = 2.7, μ = 0.4
logN(S)
log S

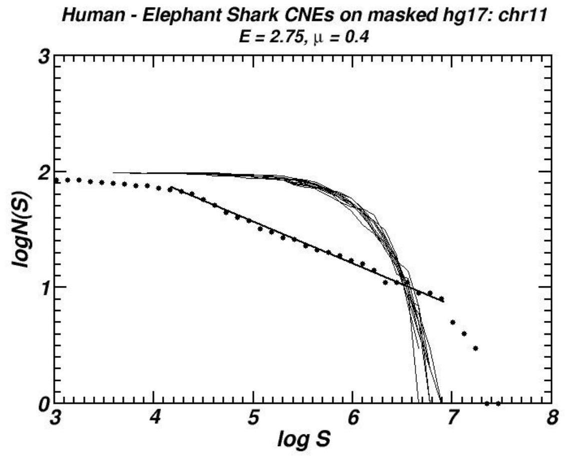
Human - Elephant Shark CNEs on masked hg17: chr11
E = 2.75, μ = 0.4
logN(S)
log S

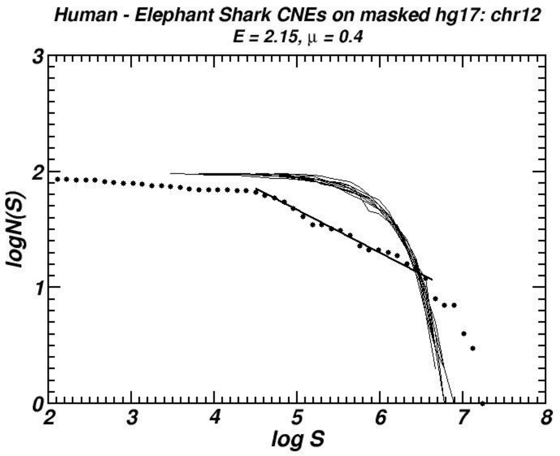
Human - Elephant Shark CNEs on masked hg17: chr12
E = 2.15, μ = 0.4
logN(S)
log S

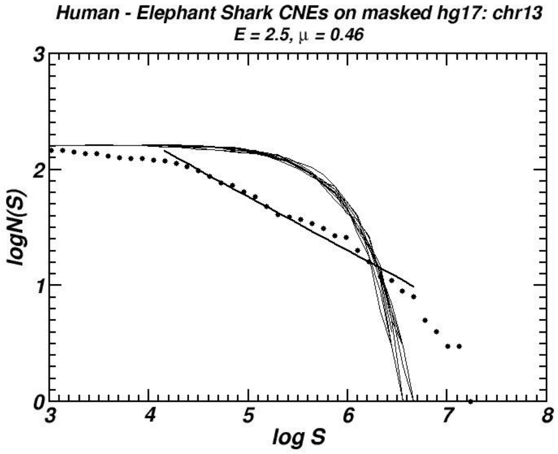
Human - Elephant Shark CNEs on masked hg17: chr13
E = 2.5, μ = 0.46
logN(S)
log S

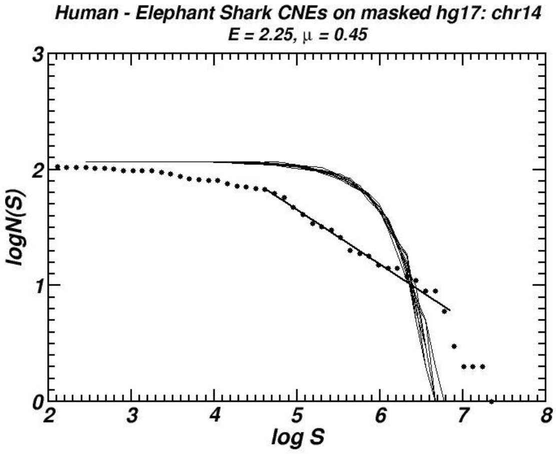
Human - Elephant Shark CNEs on masked hg17: chr14
E = 2.25, μ = 0.45
logN(S)
log S

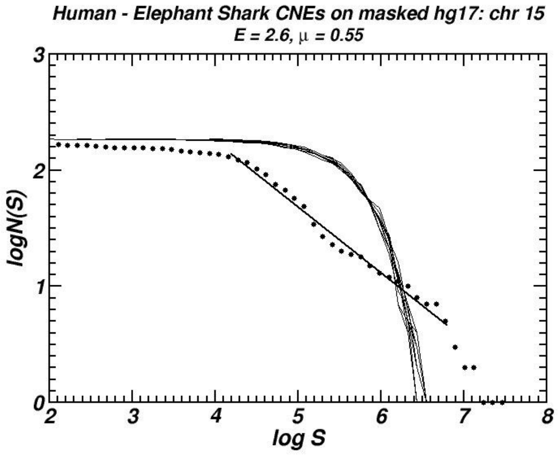
Human - Elephant Shark CNEs on masked hg17: chr 15
E = 2.6, μ = 0.55
logN(S)
log S

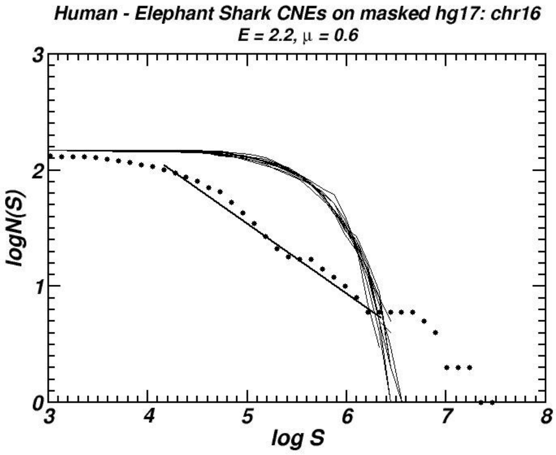
Human - Elephant Shark CNEs on masked hg17: chr16
E = 2.2, μ = 0.6
logN(S)
log S

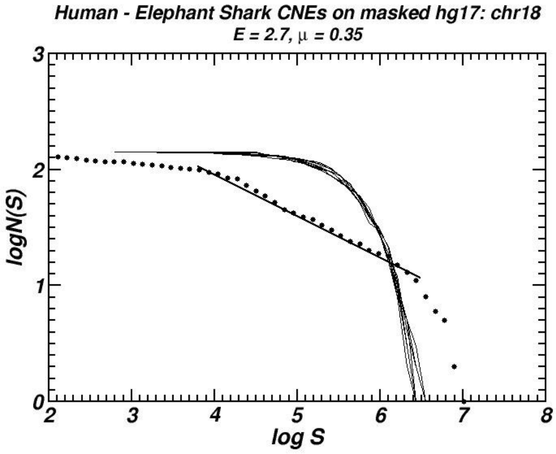
Human - Elephant Shark CNEs on masked hg17: chr18
E = 2.7, μ = 0.35
logN(S)
log S

Human-Chicken UCNEs (Dataset iii)

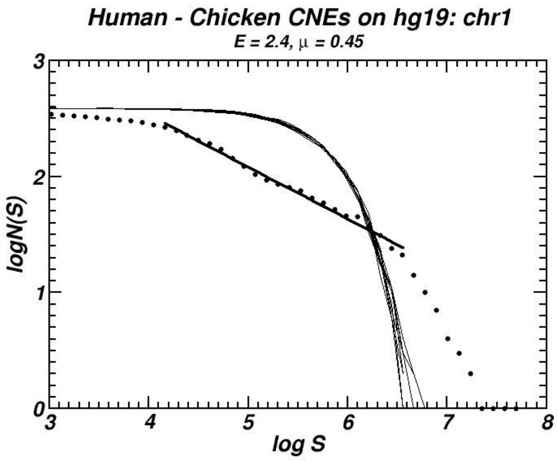

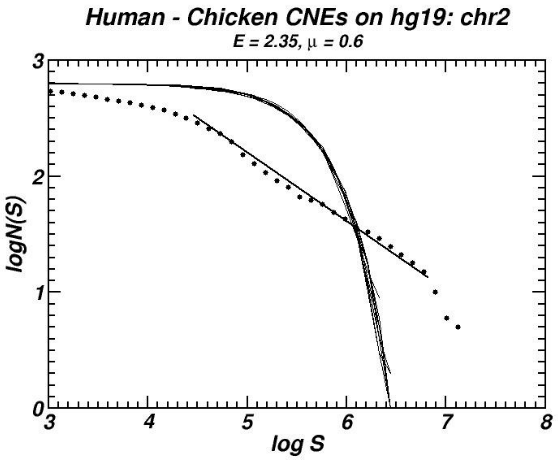

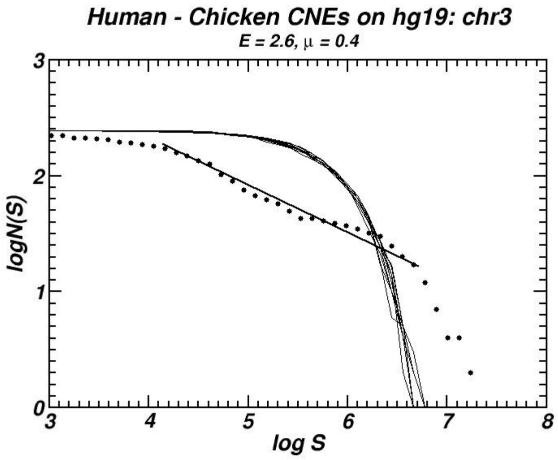

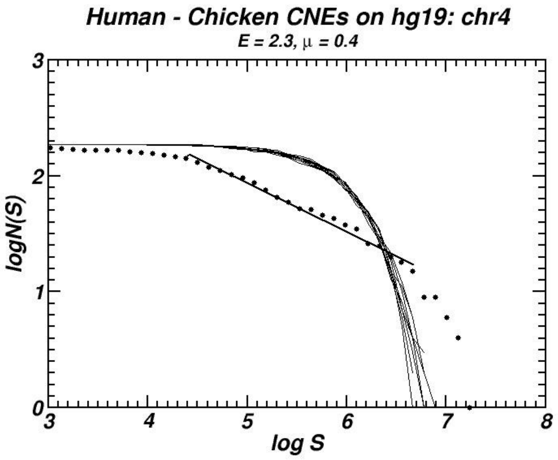

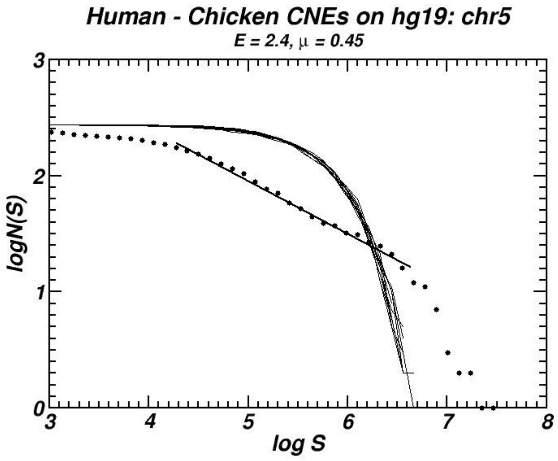

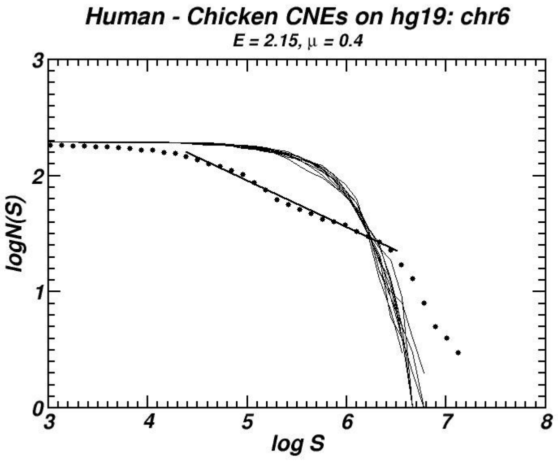

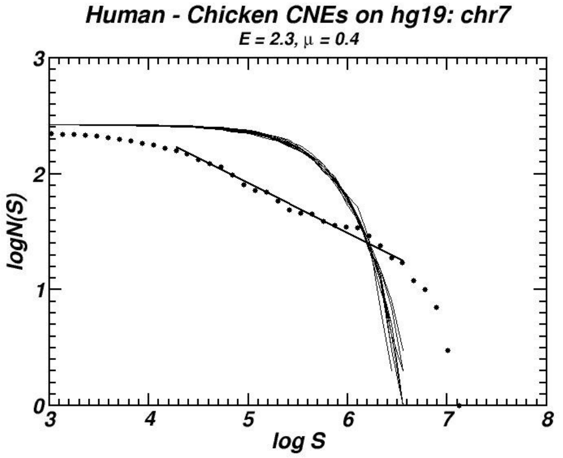
Human - Chicken CNEs on hg19: chr7
E = 2.3, μ = 0.4
logN(S)
log S

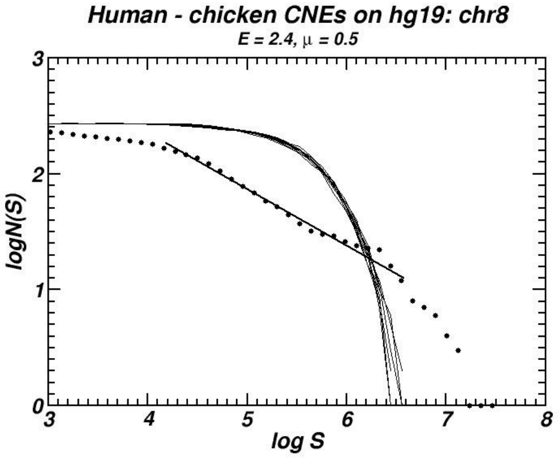
Human - chicken CNEs on hg19: chr8
E = 2.4, μ = 0.5
logN(S)
log S

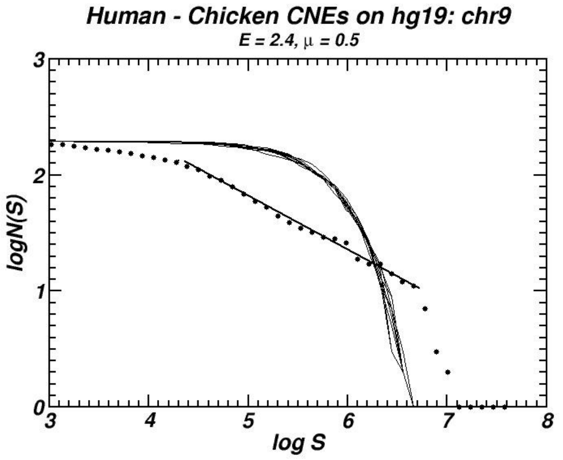
Human - Chicken CNEs on hg19: chr9
E = 2.4, μ = 0.5
logN(S)
log S

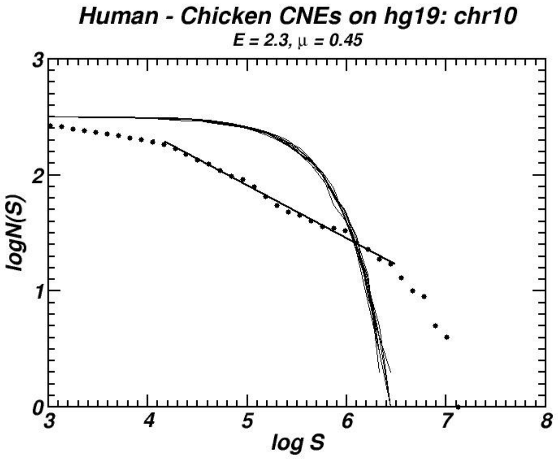
Human - Chicken CNEs on hg19: chr10
E = 2.3, μ = 0.45
logN(S)
log S

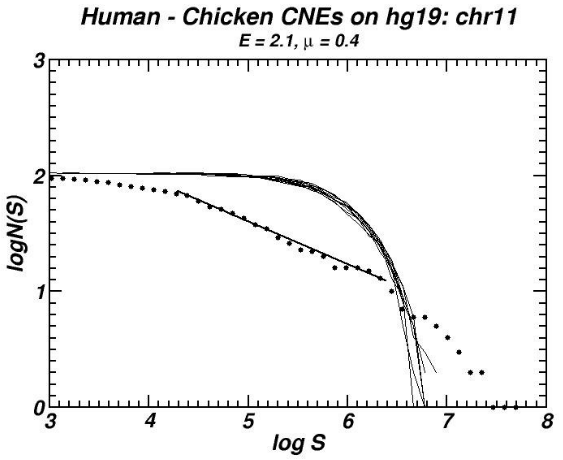
Human - Chicken CNEs on hg19: chr11
E = 2.1, μ = 0.4
logN(S)
log S

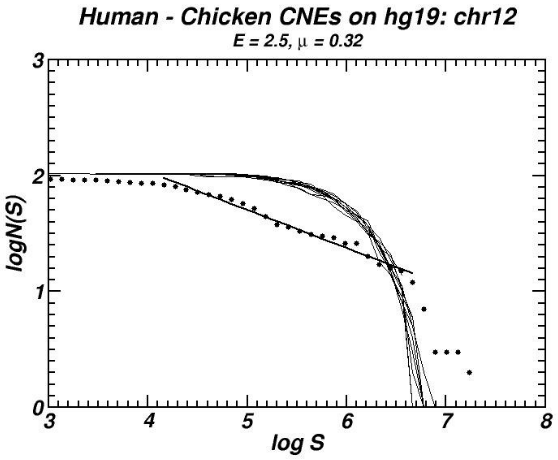
Human - Chicken CNEs on hg19: chr12
E = 2.5, μ = 0.32
logN(S)
log S

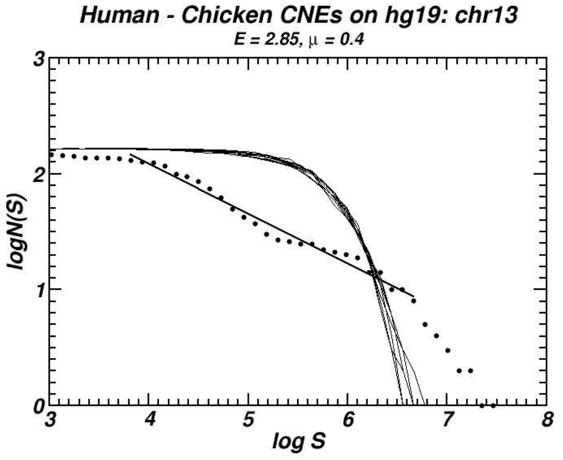
Human - Chicken CNEs on hg19: chr13
E = 2.85, μ = 0.4
logN(S)
log S

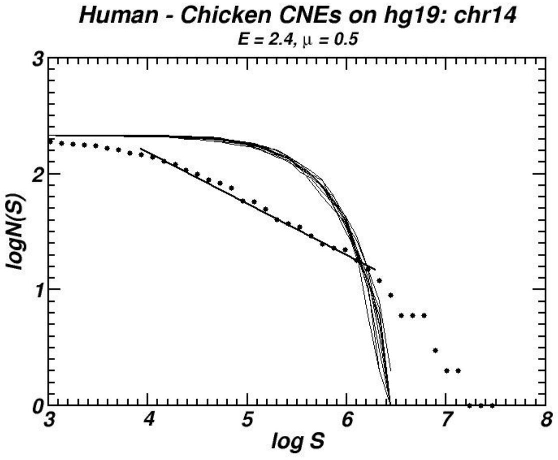
Human - Chicken CNEs on hg19: chr14
E = 2.4, μ = 0.5
logN(S)
log S

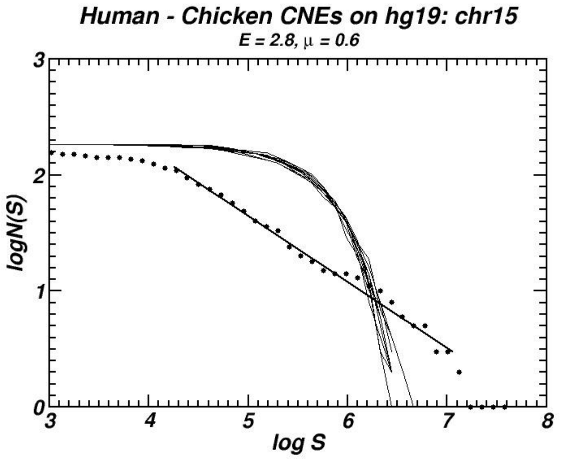
Human - Chicken CNEs on hg19: chr15
E = 2.8, μ = 0.6
logN(S)
log S

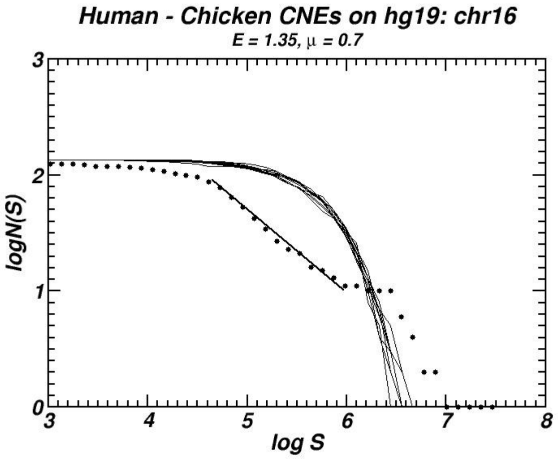
Human - Chicken CNEs on hg19: chr16
E = 1.35, μ = 0.7
logN(S)
log S

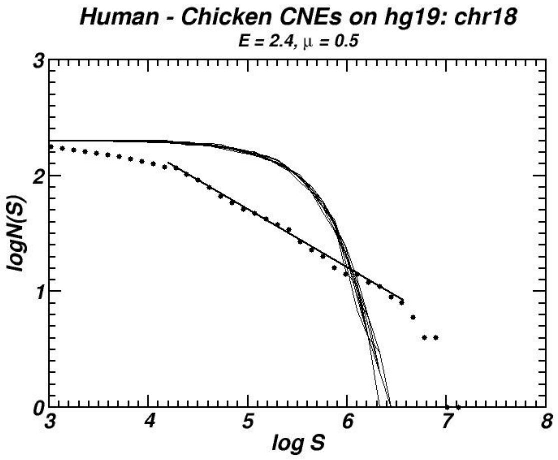
Human - Chicken CNEs on hg19: chr18
E = 2.4, μ = 0.5
logN(S)
log S

Insect CNEs (Dataset viii)

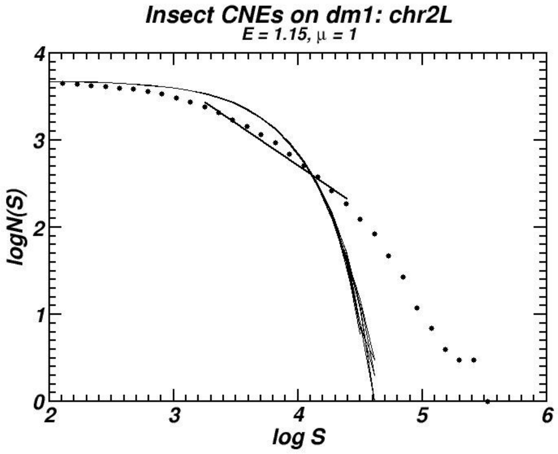

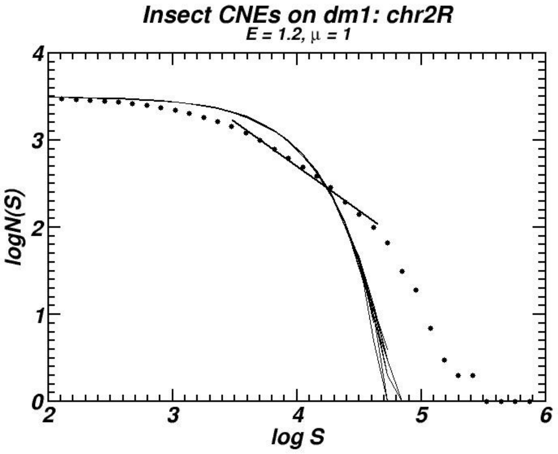

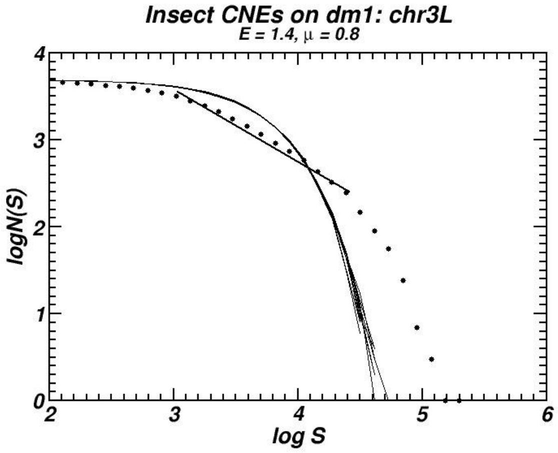

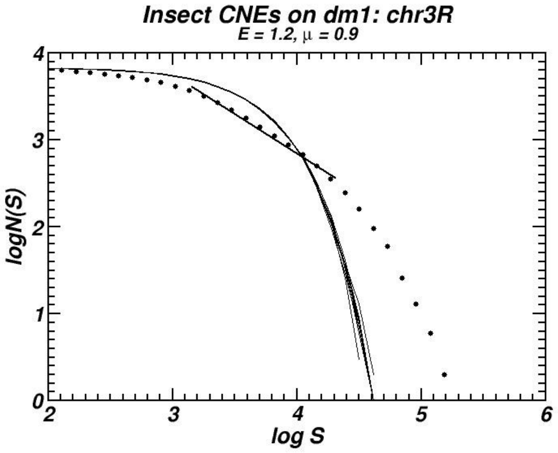

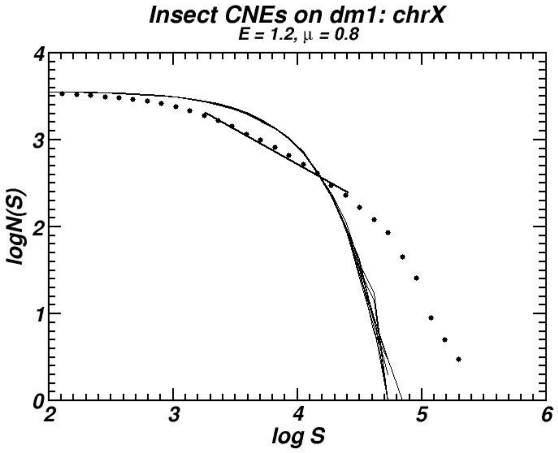

Insect CNEs on gene-masked genome

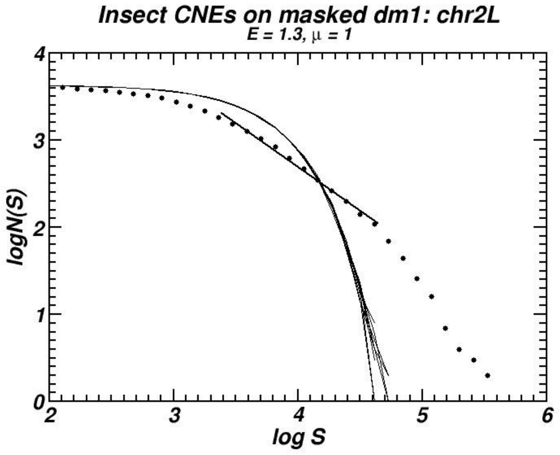

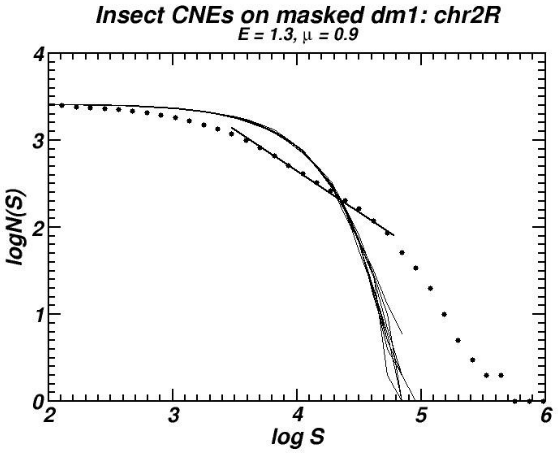

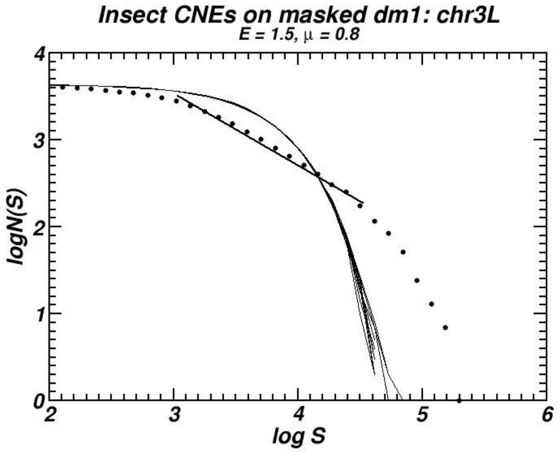

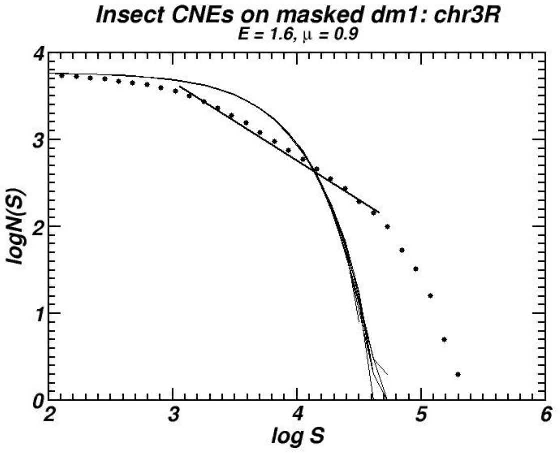

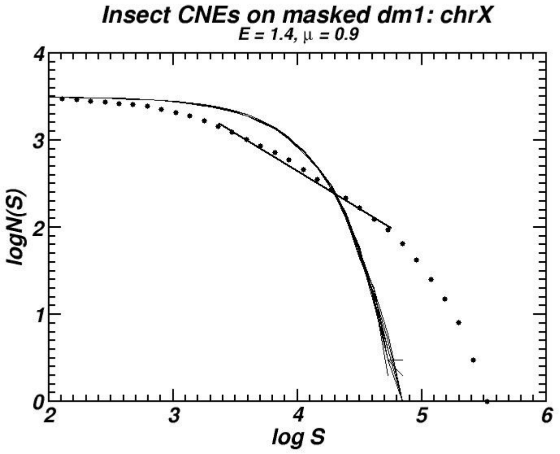

D.rerio PCNEs (Dataset vii)

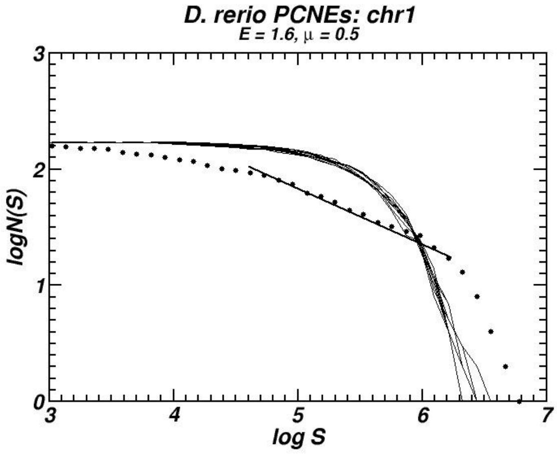

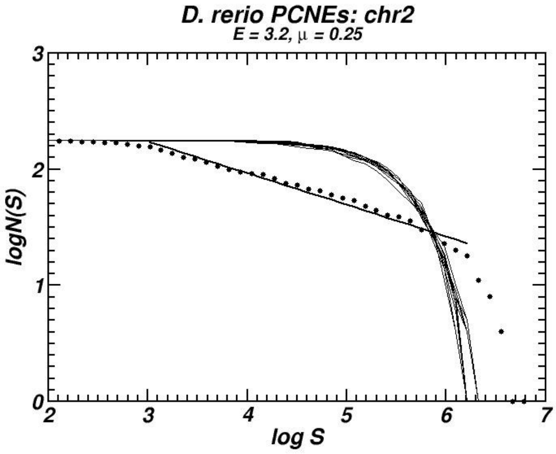

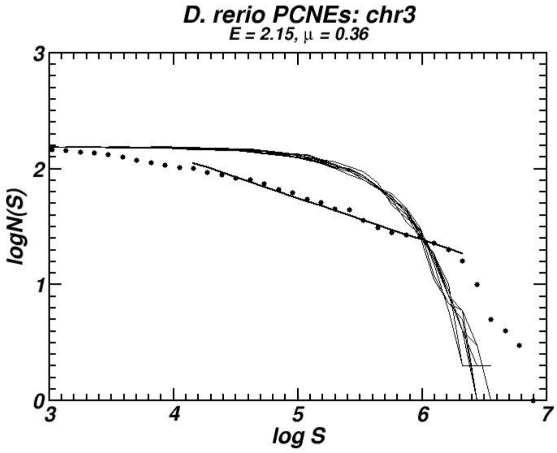

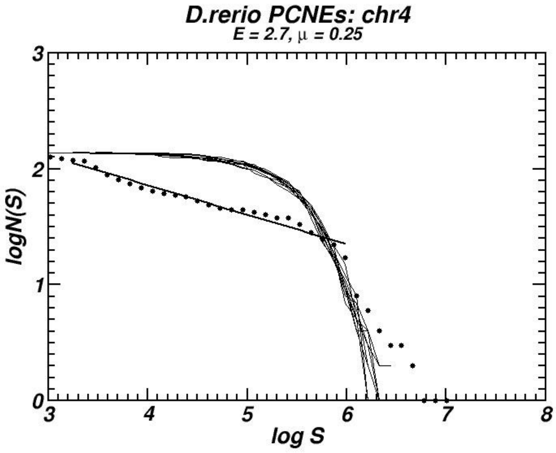

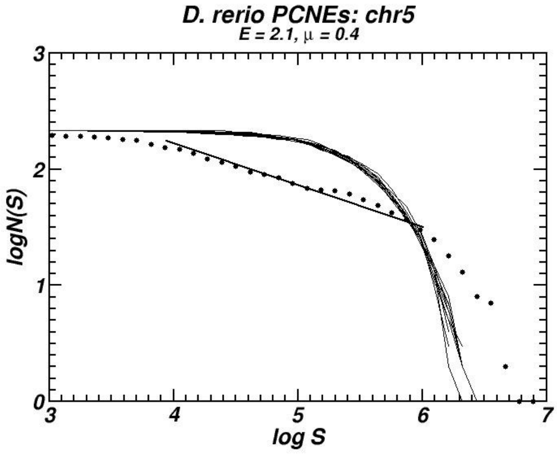

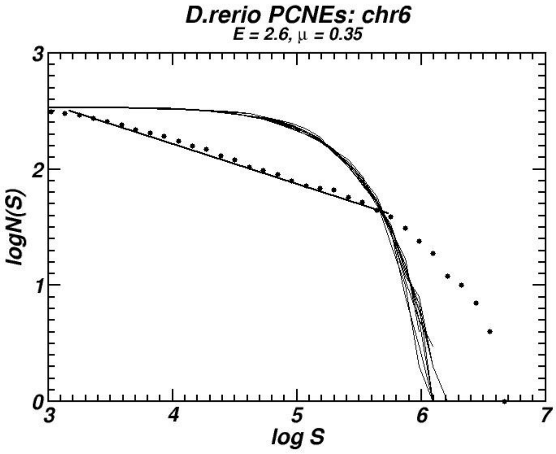

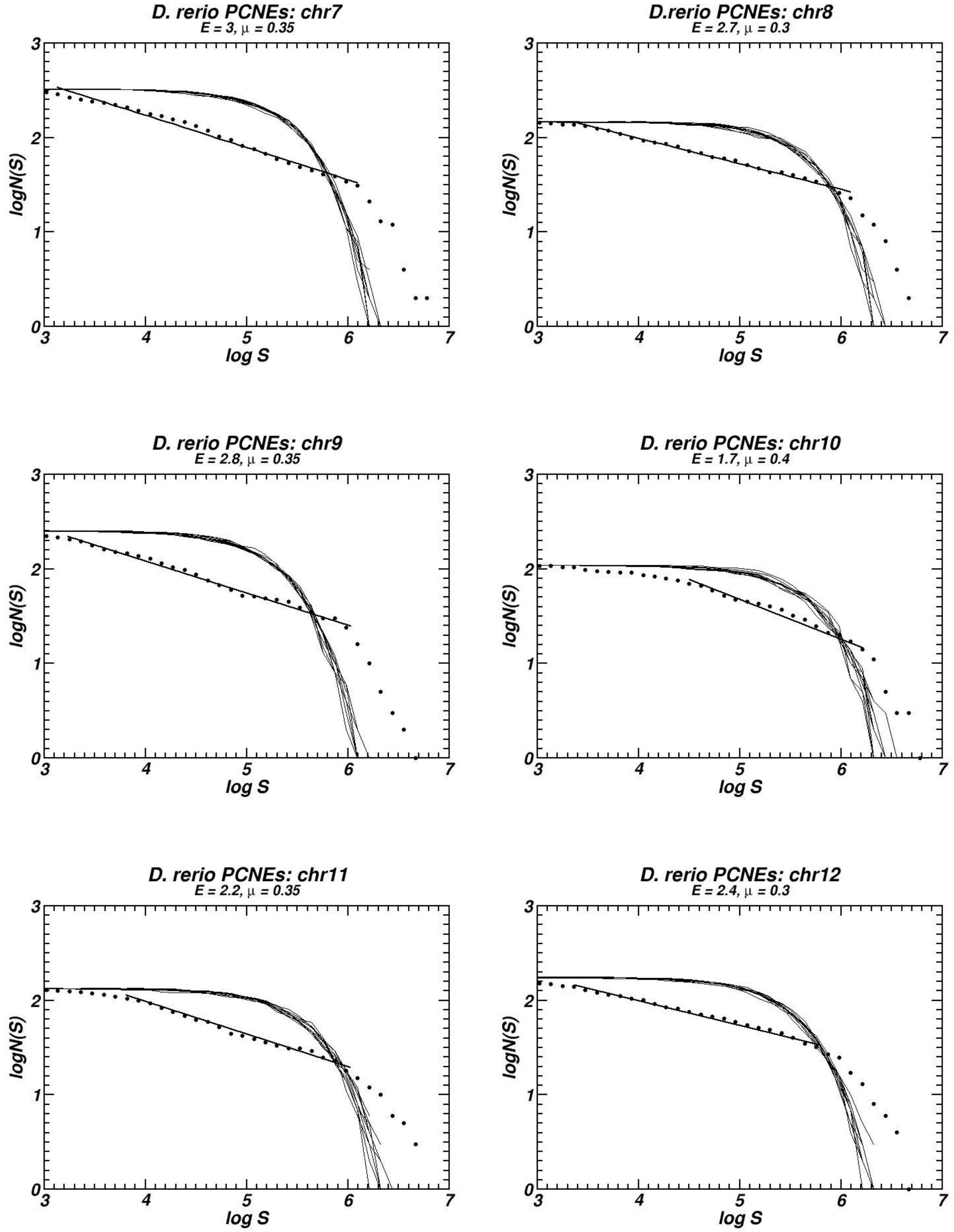
D. rerio PCNEs: chr7
E = 3, μ = 0.35
D.rerio PCNEs: chr8
E = 2.7, μ = 0.3
D. rerio PCNEs: chr9
E = 2.8, μ = 0.35
D. rerio PCNEs: chr10
E = 1.7, μ = 0.4
D. rerio PCNEs: chr11
E = 2.2, μ = 0.35
D. rerio PCNEs: chr12
E = 2.4, μ = 0.3
logN(S)
log S
0
1
2
3
4
5
6
7

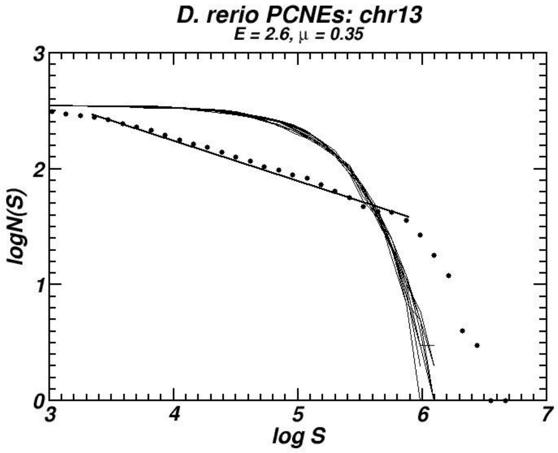
D. rerio PCNEs: chr13
E = 2.6, μ = 0.35
logN(S)
log S
0
1
2
3
3
4
5
6
7

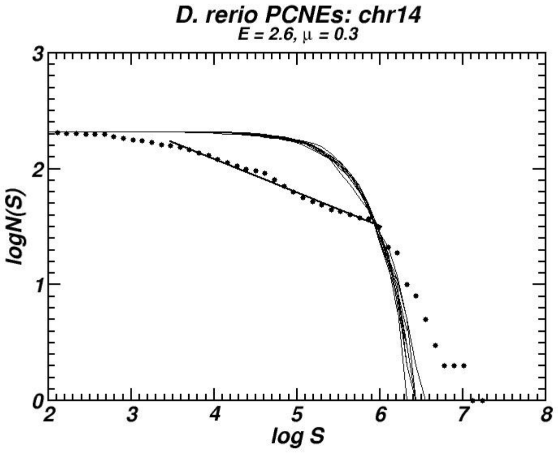
D. rerio PCNEs: chr14
E = 2.6, μ = 0.3
logN(S)
log S
0
1
2
3
2
3
4
5
6
7
8

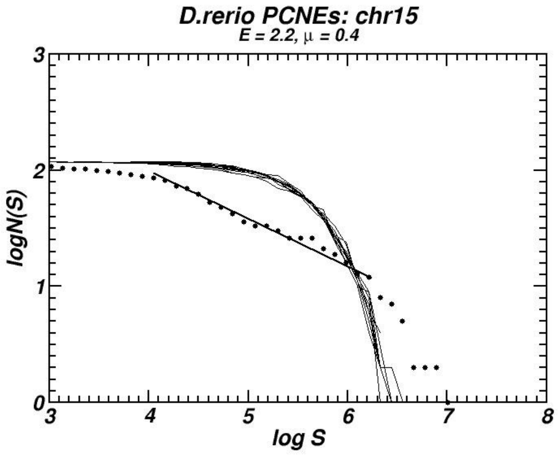
D.rerio PCNEs: chr15
E = 2.2, μ = 0.4
logN(S)
log S
0
1
2
3
3
4
5
6
7
8

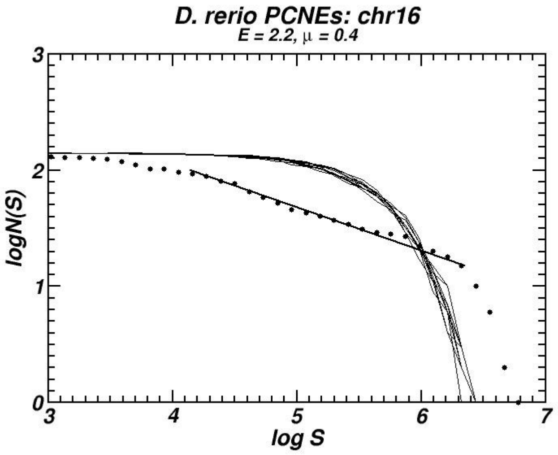
D. rerio PCNEs: chr16
E = 2.2, μ = 0.4
logN(S)
log S
0
1
2
3
3
4
5
6
7

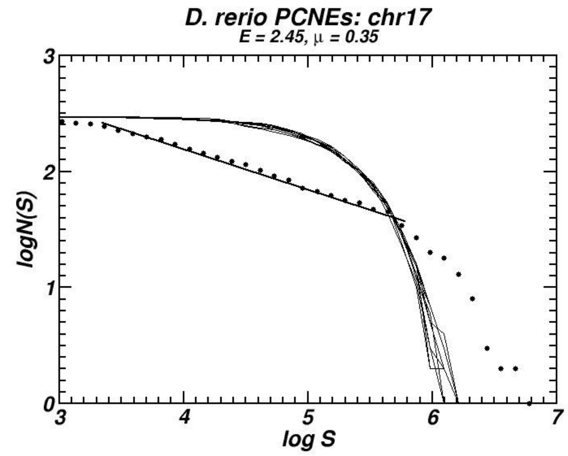
D. rerio PCNEs: chr17
E = 2.45, μ = 0.35
logN(S)
log S
0
1
2
3
3
4
5
6
7

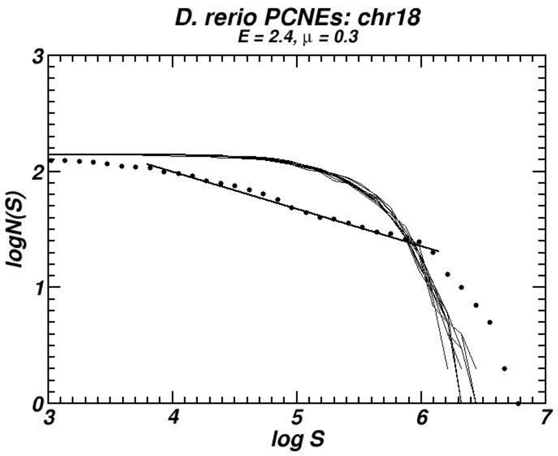
D. rerio PCNEs: chr18
E = 2.4, μ = 0.3
logN(S)
log S
0
1
2
3
3
4
5
6
7

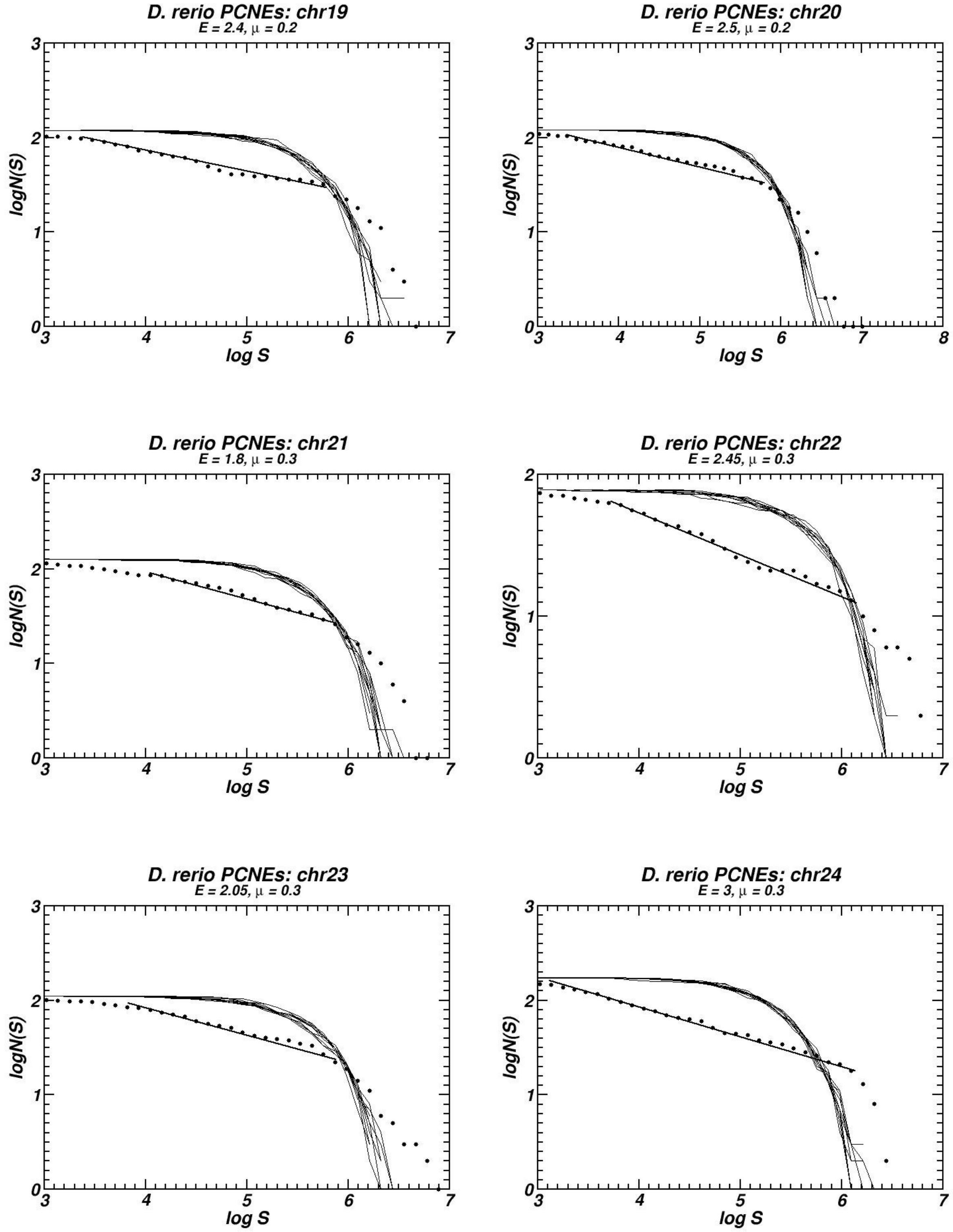
D. rerio PCNEs: chr19
E = 2.4, μ = 0.2
logN(S)
log S
D. rerio PCNEs: chr20
E = 2.5, μ = 0.2
logN(S)
log S
D. rerio PCNEs: chr21
E = 1.8, μ = 0.3
logN(S)
log S
D. rerio PCNEs: chr22
E = 2.45, μ = 0.3
logN(S)
log S
D. rerio PCNEs: chr23
E = 2.05, μ = 0.3
logN(S)
log S
D. rerio PCNEs: chr24
E = 3, μ = 0.3
logN(S)
log S

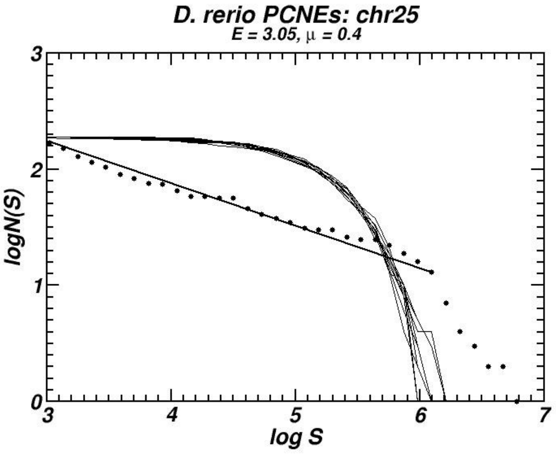

D. rerio PCNEs: chr25
E = 3.05, μ = 0.4
logN(S)
log S
3
2
1
0
3
4
5
6
7

Human-Rodents CNEs (Dataset x)

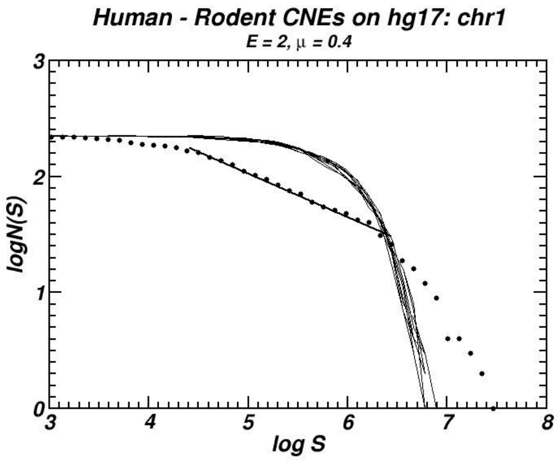

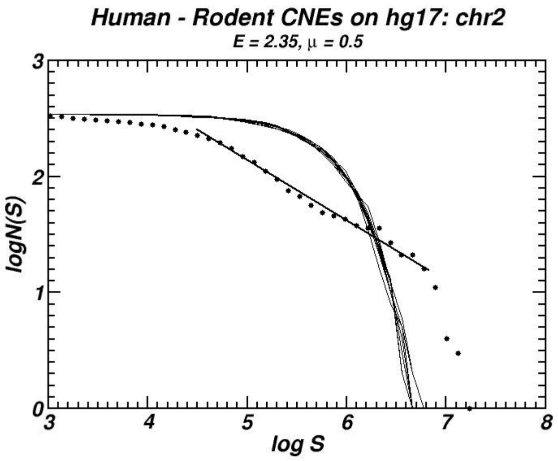

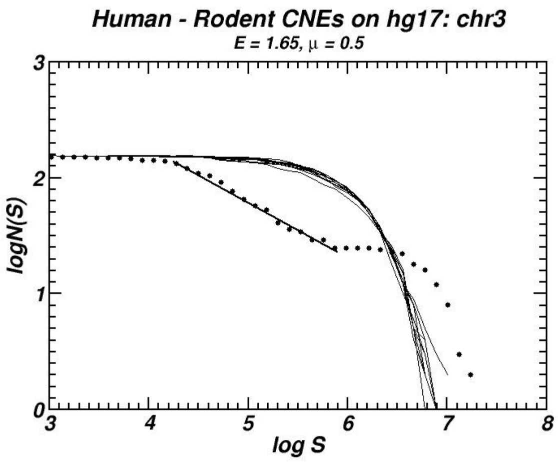

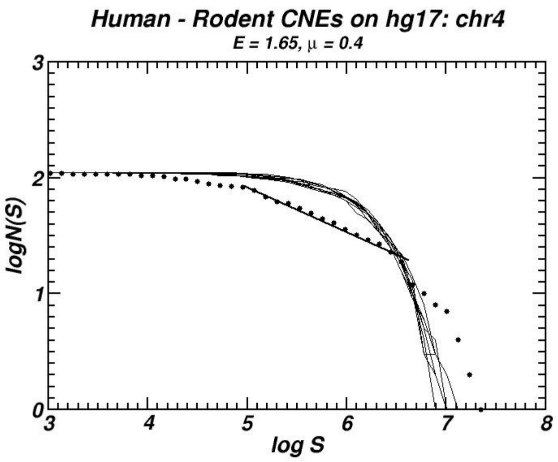

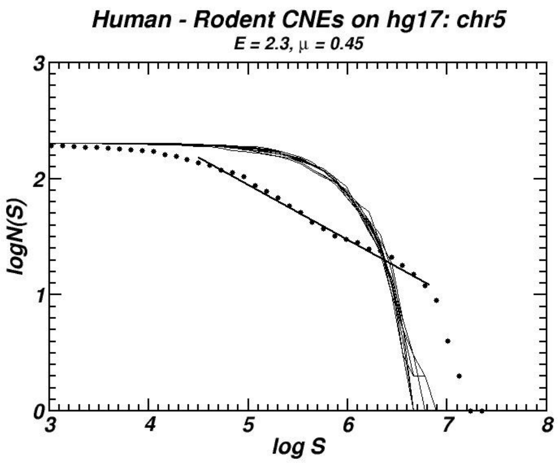

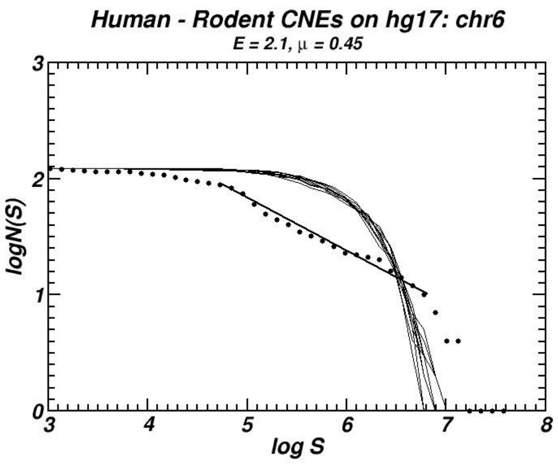

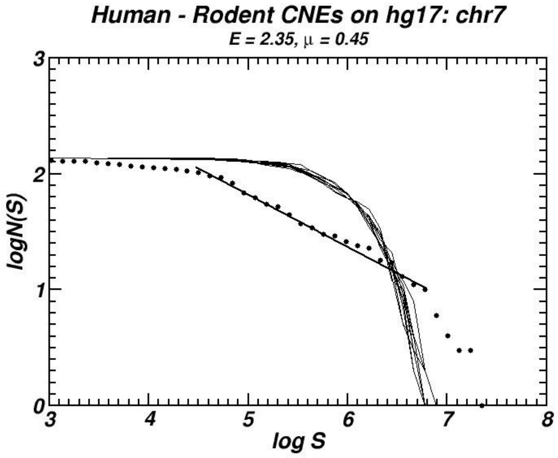

Human - Rodent CNEs on hg17: chr7
E = 2.35, μ = 0.45
logN(S)
log S

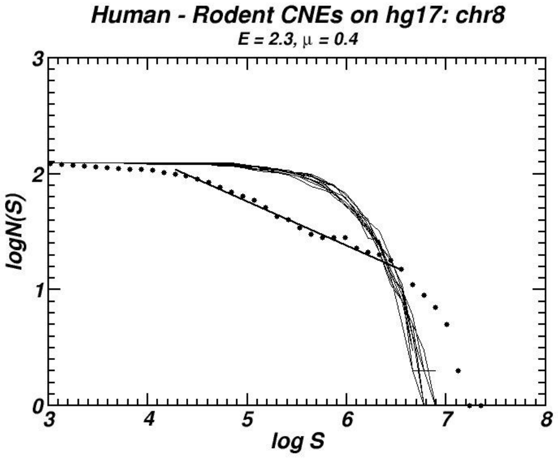

Human - Rodent CNEs on hg17: chr8
E = 2.3, μ = 0.4
logN(S)
log S

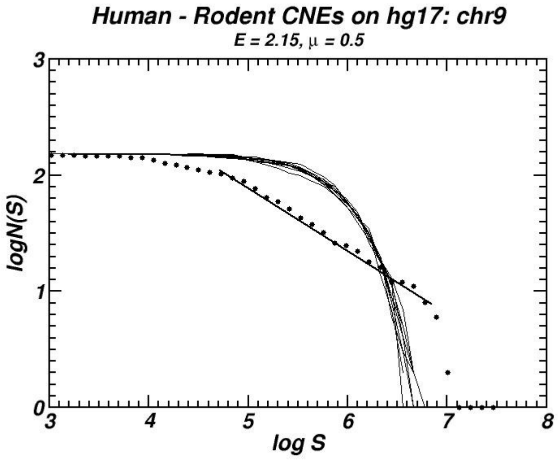

Human - Rodent CNEs on hg17: chr9
E = 2.15, μ = 0.5
logN(S)
log S

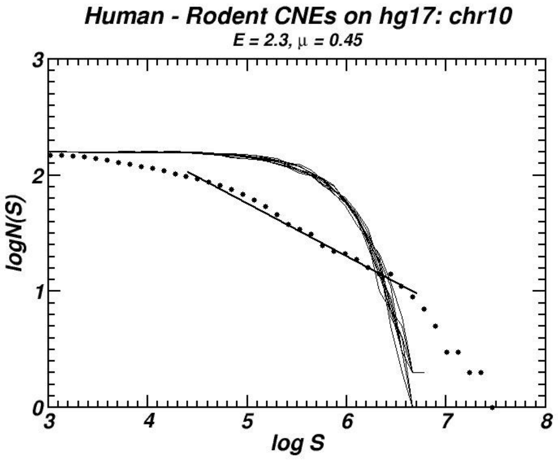

Human - Rodent CNEs on hg17: chr10
E = 2.3, μ = 0.45
logN(S)
log S

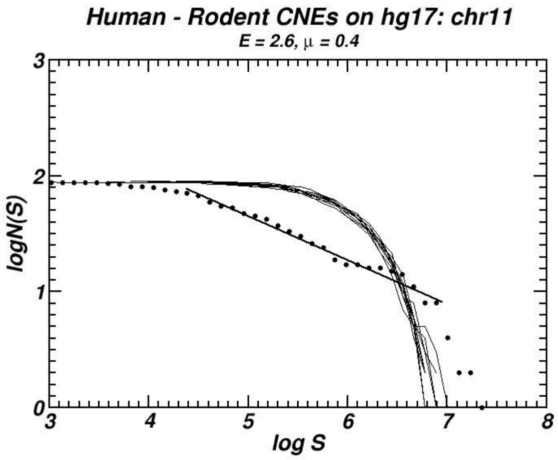

Human - Rodent CNEs on hg17: chr11
E = 2.6, μ = 0.4
logN(S)
log S

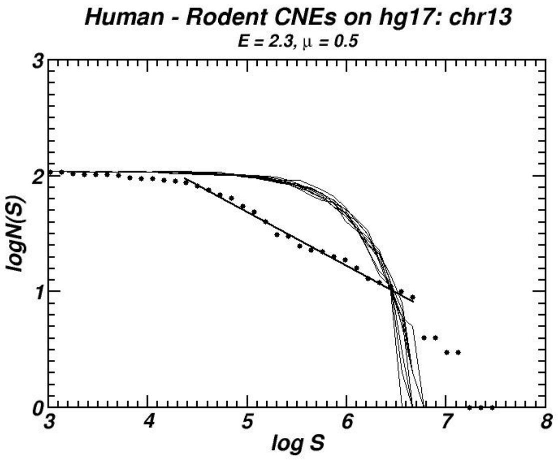

Human - Rodent CNEs on hg17: chr13
E = 2.3, μ = 0.5
logN(S)
log S

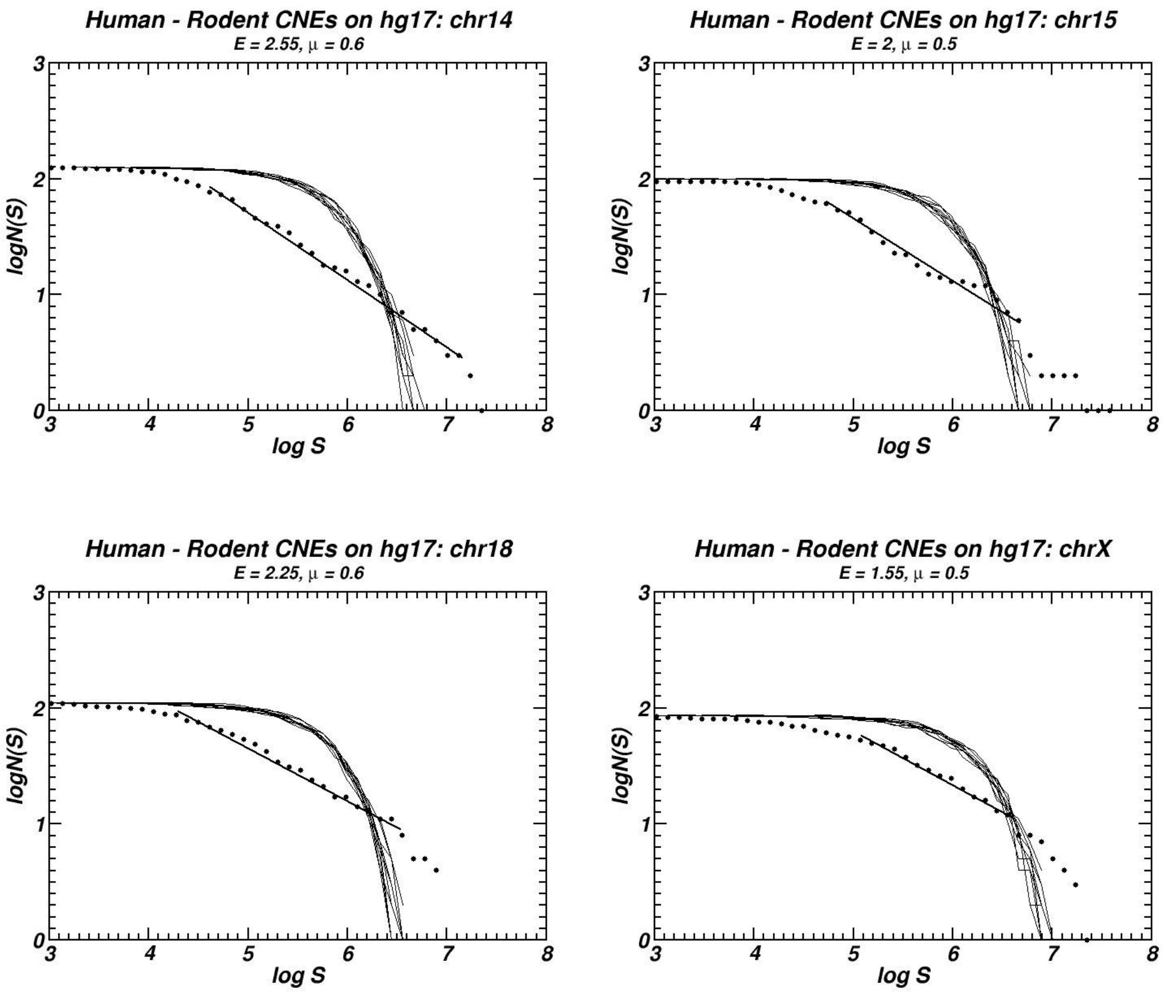

Human - Rodent CNEs on hg17: chr14
E = 2.55, μ = 0.6
logN(S)
log S
Human - Rodent CNEs on hg17: chr15
E = 2, μ = 0.5
logN(S)
log S
Human - Rodent CNEs on hg17: chr18
E = 2.25, μ = 0.6
logN(S)
log S
Human - Rodent CNEs on hg17: chrX
E = 1.55, μ = 0.5
logN(S)
log S

EU-FR CNEs (Dataset ia)

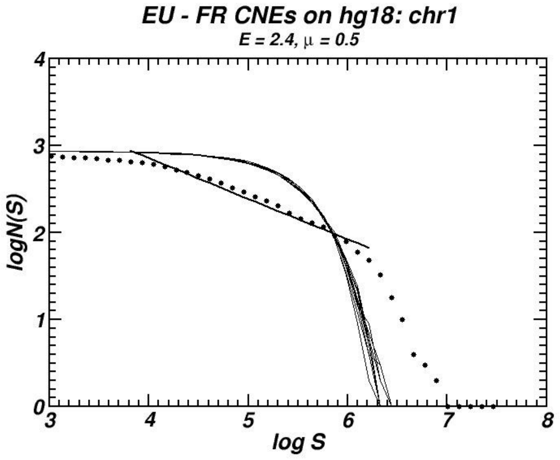

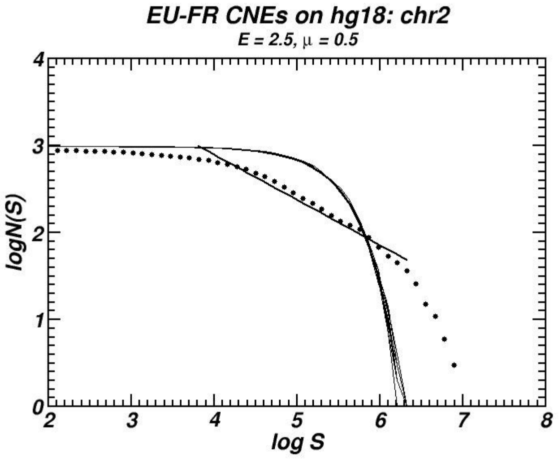

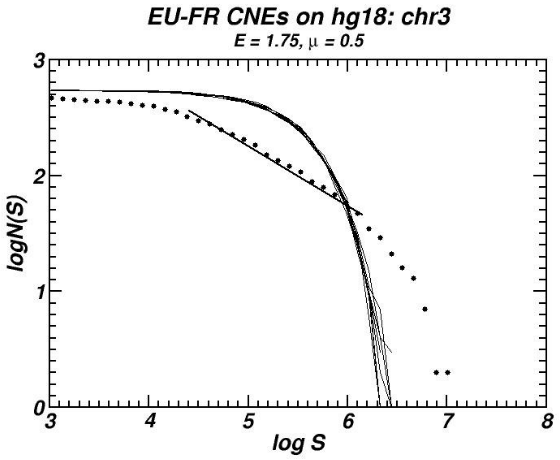

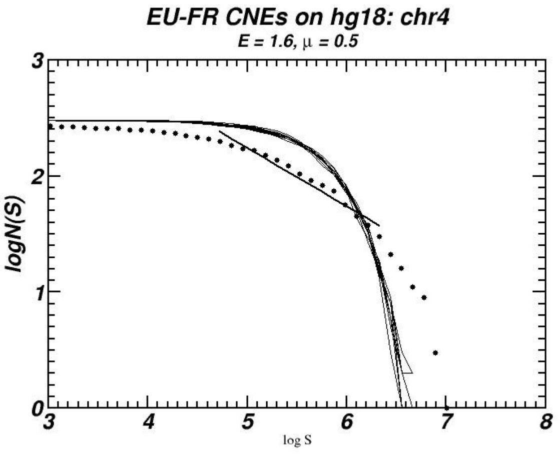

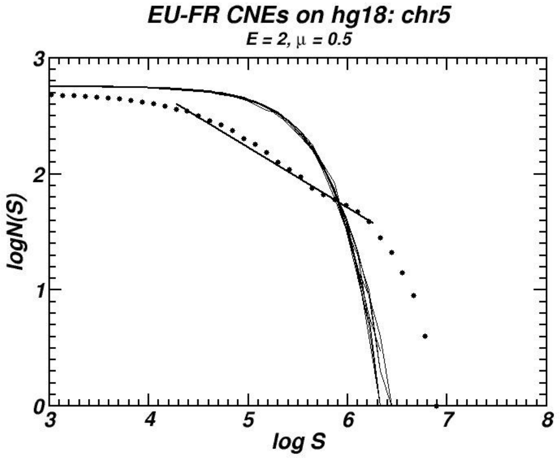

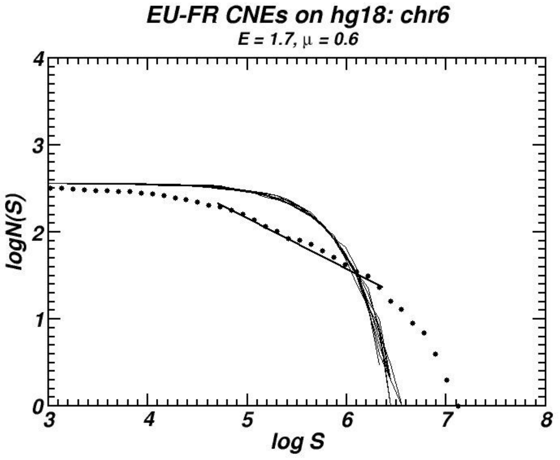

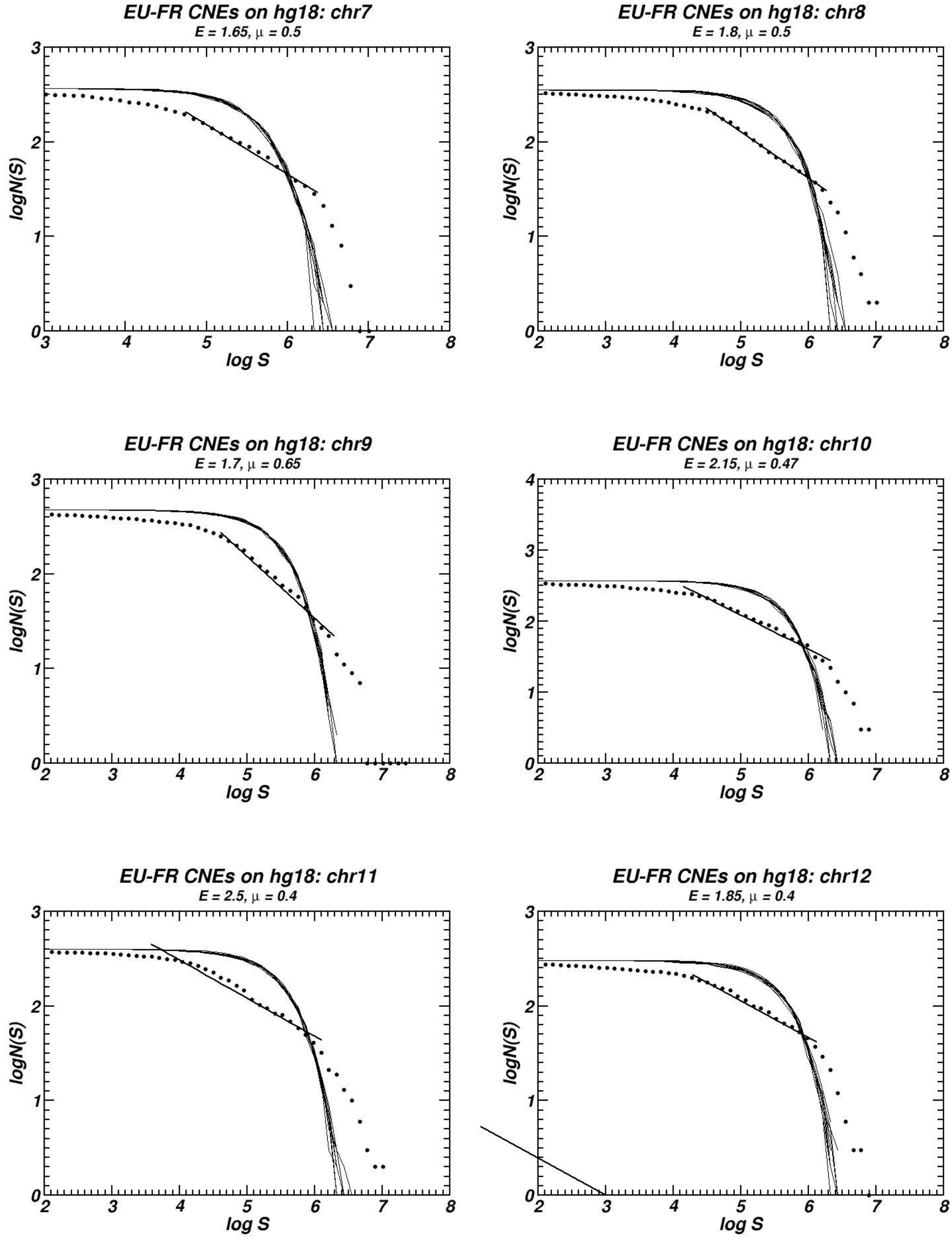

EU-FR CNEs on hg18: chr7
E = 1.65, μ = 0.5
EU-FR CNEs on hg18: chr8
E = 1.8, μ = 0.5
EU-FR CNEs on hg18: chr9
E = 1.7, μ = 0.65
EU-FR CNEs on hg18: chr10
E = 2.15, μ = 0.47
EU-FR CNEs on hg18: chr11
E = 2.5, μ = 0.4
EU-FR CNEs on hg18: chr12
E = 1.85, μ = 0.4
logN(S)
log S

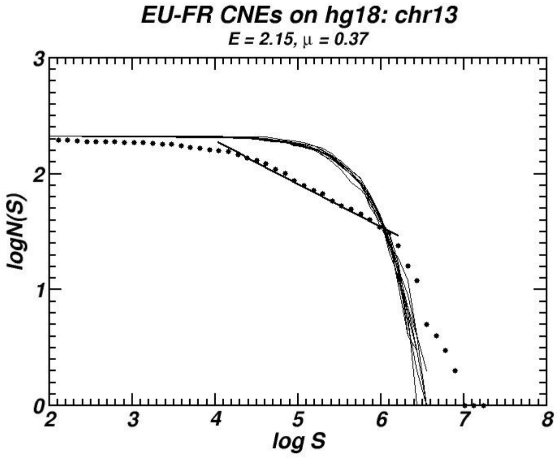
EU-FR CNEs on hg18: chr13
E = 2.15, μ = 0.37
logN(S)
log S

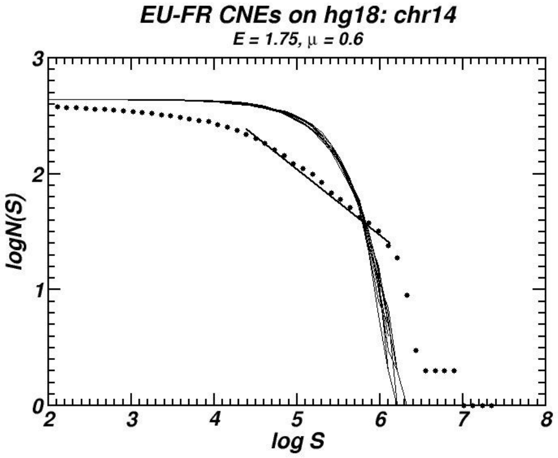
EU-FR CNEs on hg18: chr14
E = 1.75, μ = 0.6
logN(S)
log S

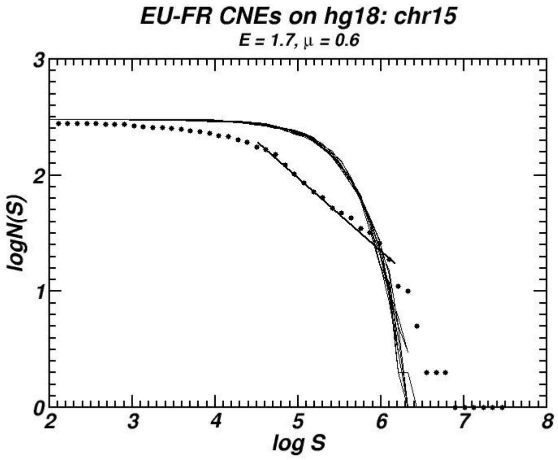
EU-FR CNEs on hg18: chr15
E = 1.7, μ = 0.6
logN(S)
log S

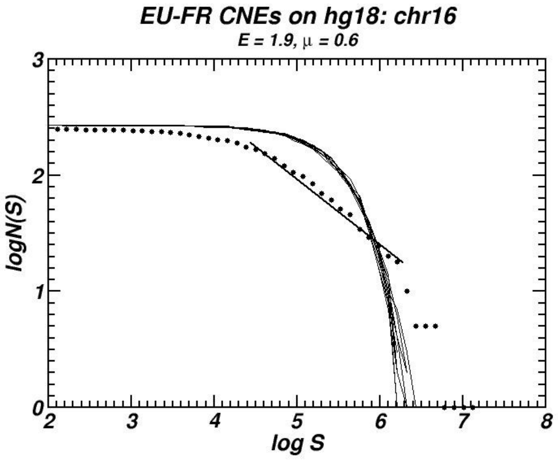
EU-FR CNEs on hg18: chr16
E = 1.9, μ = 0.6
logN(S)
log S

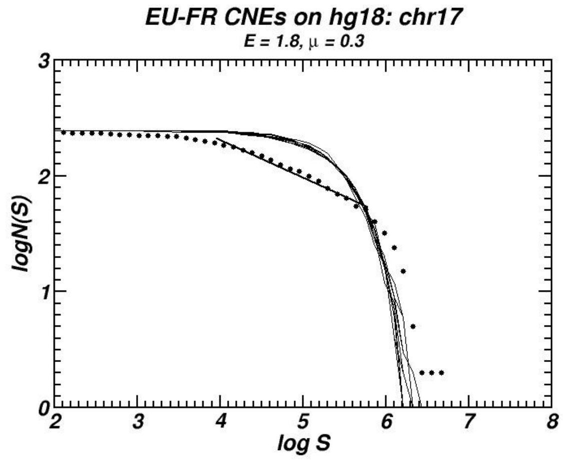
EU-FR CNEs on hg18: chr17
E = 1.8, μ = 0.3
logN(S)
log S

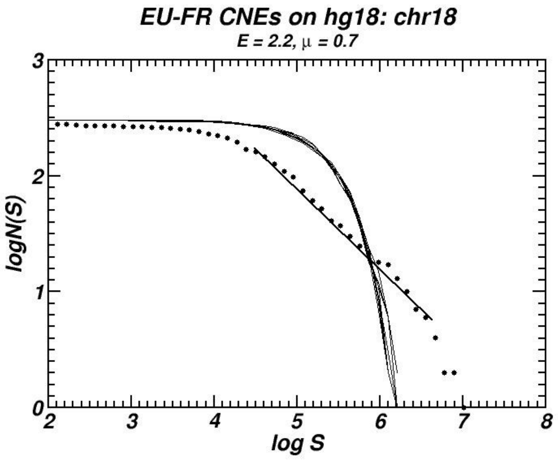
EU-FR CNEs on hg18: chr18
E = 2.2, μ = 0.7
logN(S)
log S

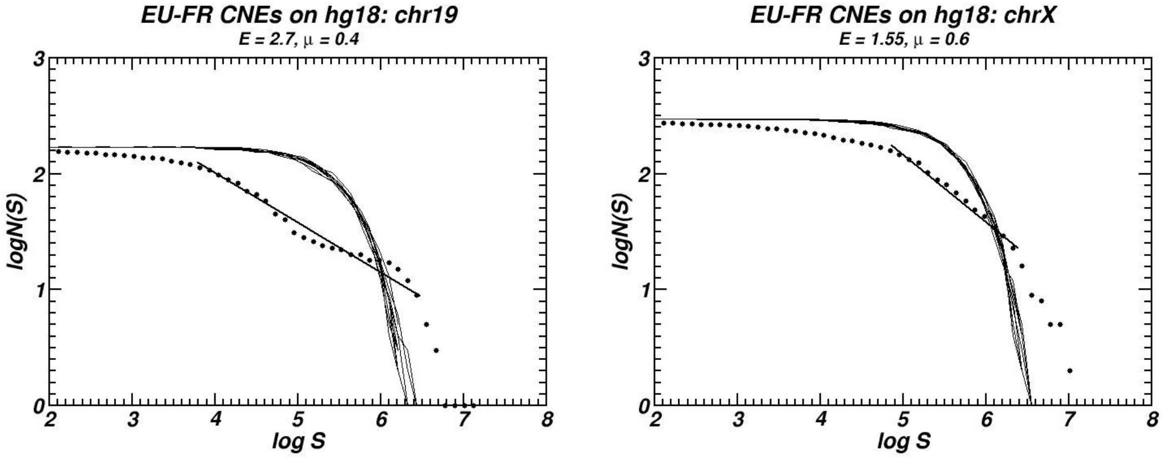
EU-FR CNEs on hg18: chr19
E = 2.7, μ = 0.4
logN(S)
log S
EU-FR CNEs on hg18: chrX
E = 1.55, μ = 0.6
logN(S)
log S

FR CNEs (Dataset ib)

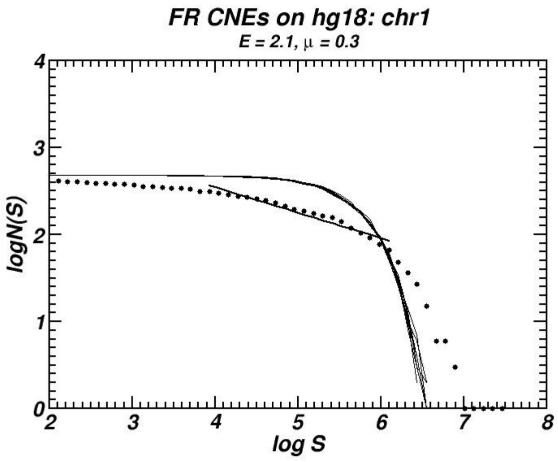

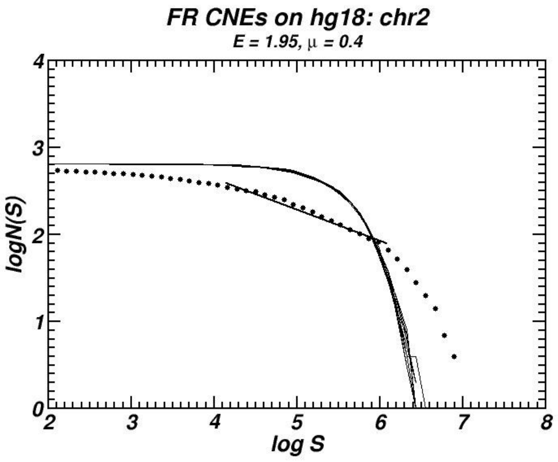

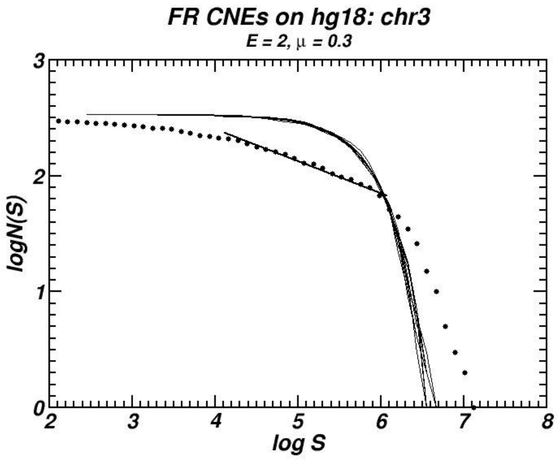

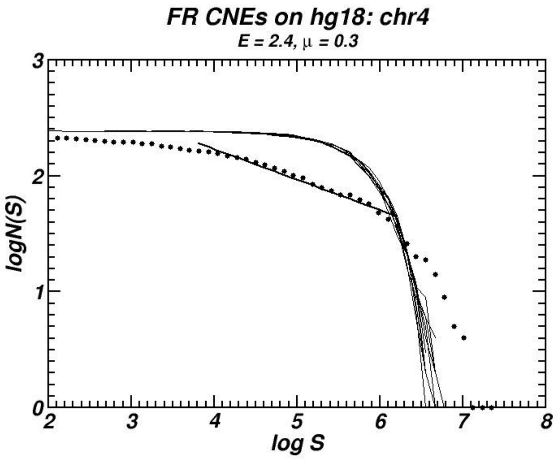

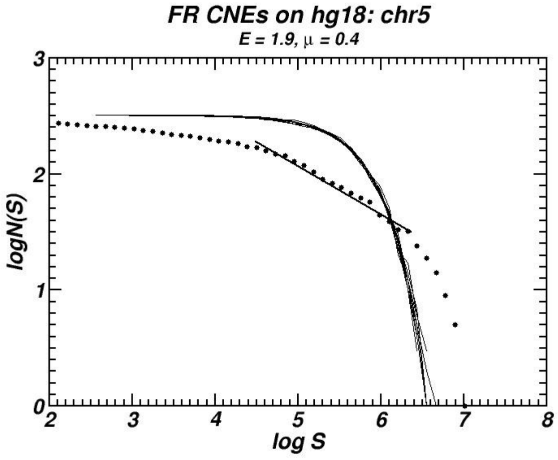

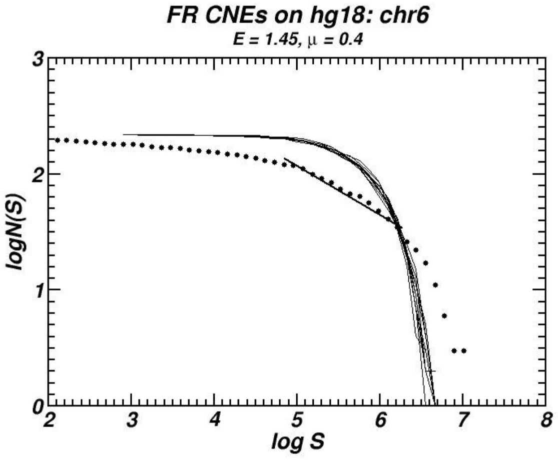

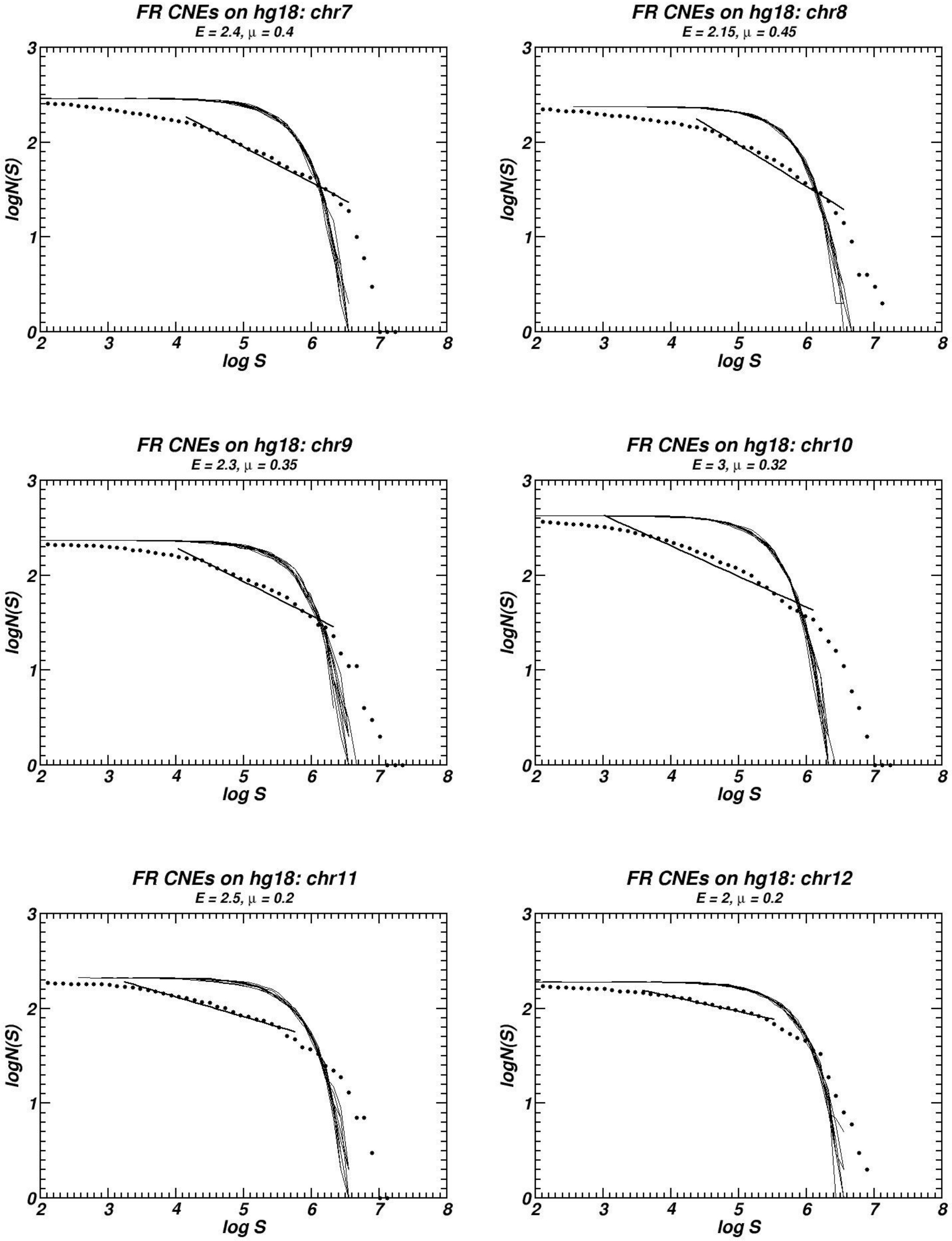
FR CNEs on hg18: chr7
E = 2.4, μ = 0.4
logN(S)
log S
FR CNEs on hg18: chr8
E = 2.15, μ = 0.45
logN(S)
log S
FR CNEs on hg18: chr9
E = 2.3, μ = 0.35
logN(S)
log S
FR CNEs on hg18: chr10
E = 3, μ = 0.32
logN(S)
log S
FR CNEs on hg18: chr11
E = 2.5, μ = 0.2
logN(S)
log S
FR CNEs on hg18: chr12
E = 2, μ = 0.2
logN(S)
log S

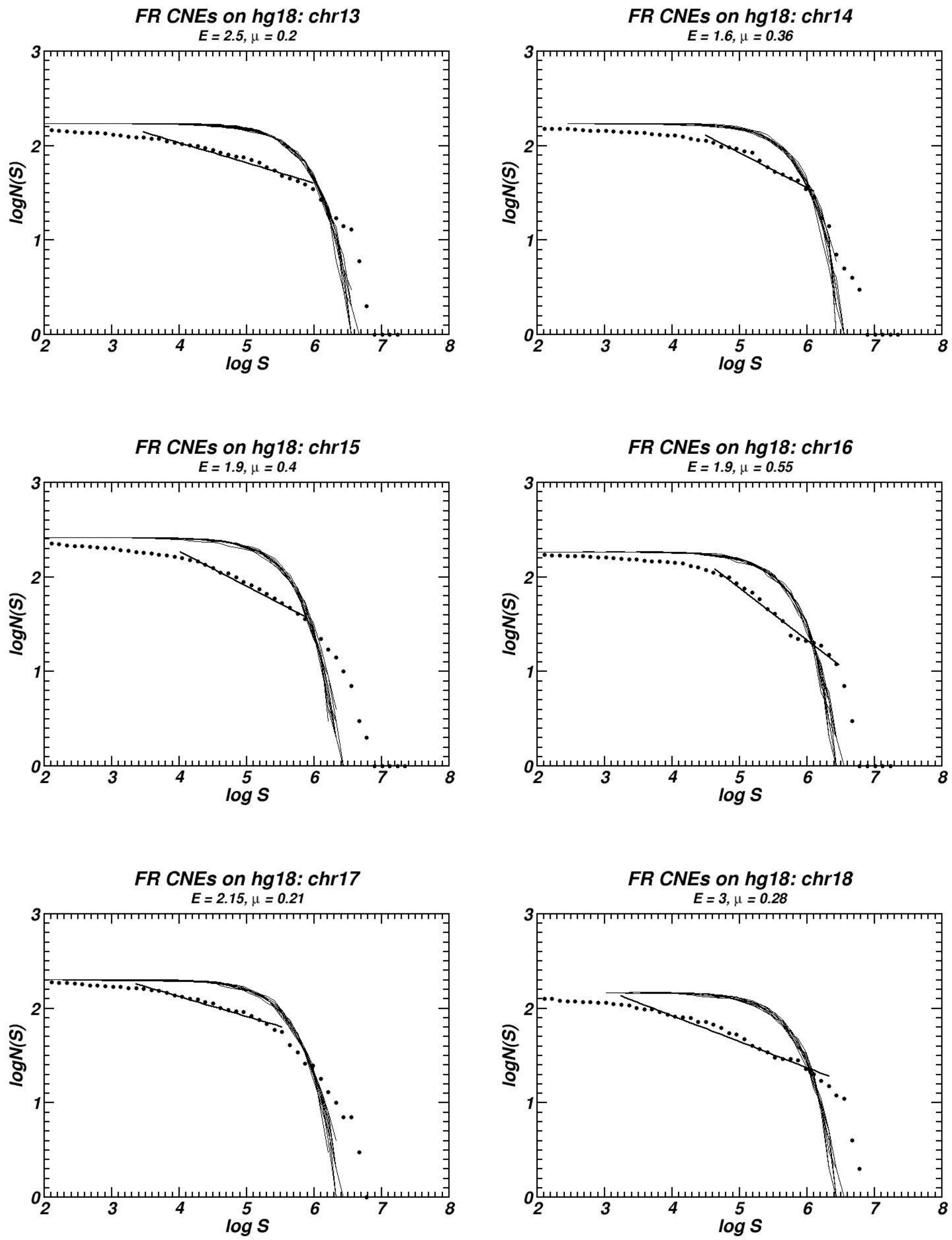

FR CNEs on hg18: chr13
E = 2.5, μ = 0.2
FR CNEs on hg18: chr14
E = 1.6, μ = 0.36
FR CNEs on hg18: chr15
E = 1.9, μ = 0.4
FR CNEs on hg18: chr16
E = 1.9, μ = 0.55
FR CNEs on hg18: chr17
E = 2.15, μ = 0.21
FR CNEs on hg18: chr18
E = 3, μ = 0.28
logN(S)
log S

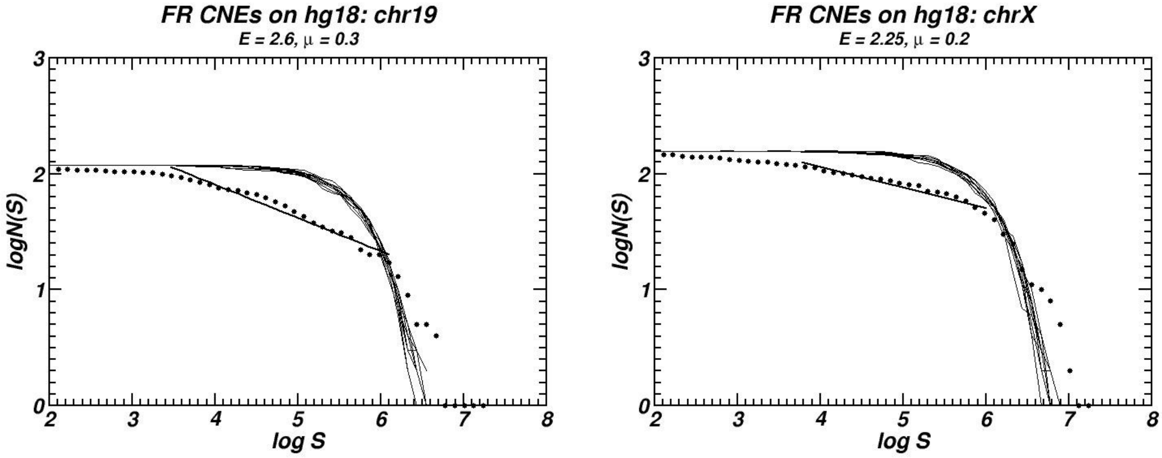
FR CNEs on hg18: chr19
E = 2.6, μ = 0.3
logN(S)
log S
FR CNEs on hg18: chrX
E = 2.25, μ = 0.2
logN(S)
log S

XT-FR CNEs (Dataset ic)

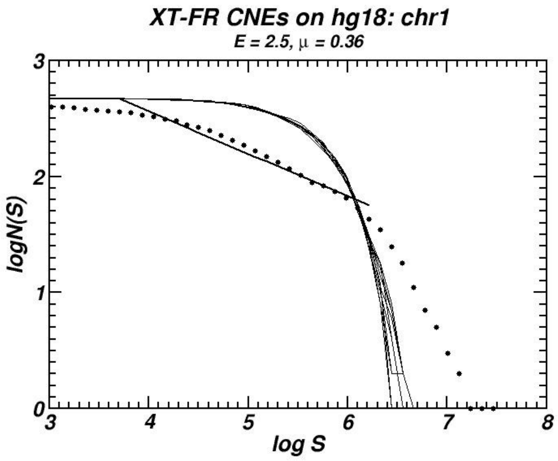

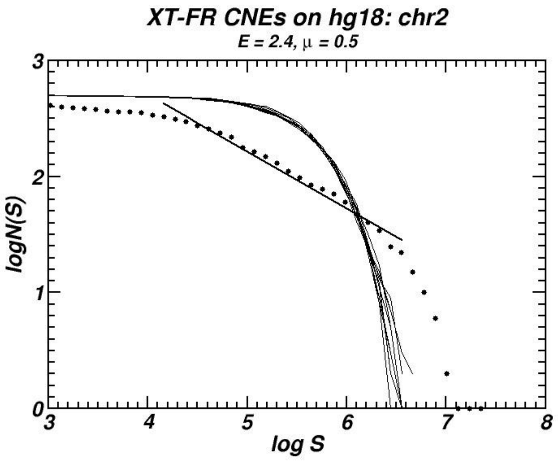

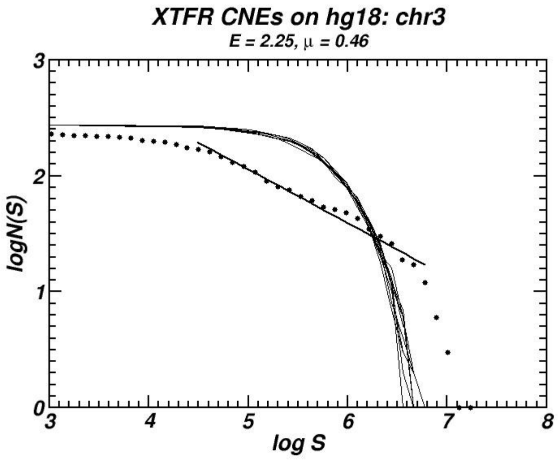

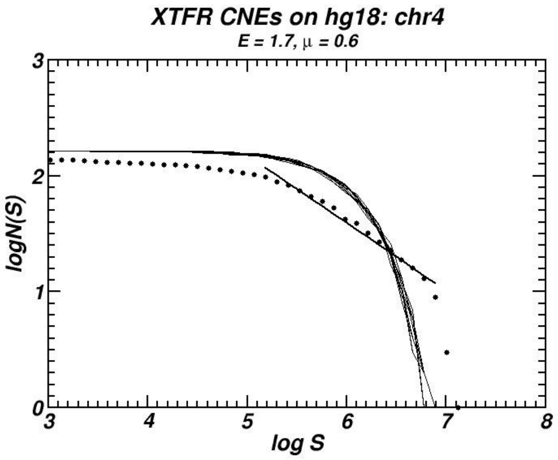

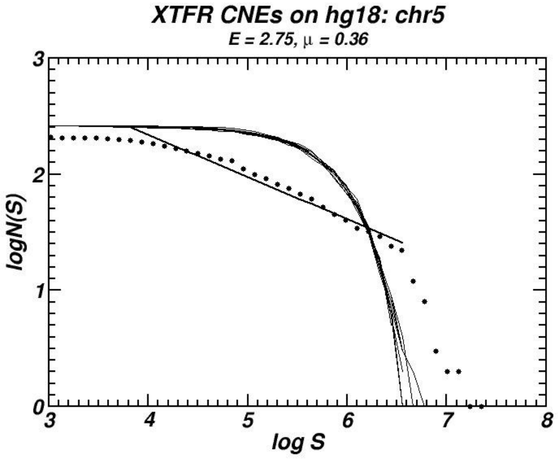

GG-XT-FR CNEs (Dataset id)

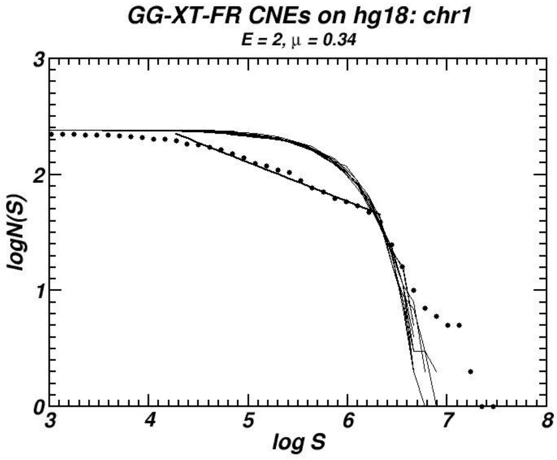

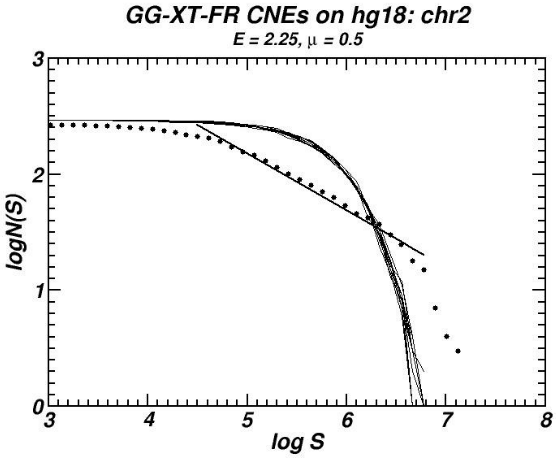

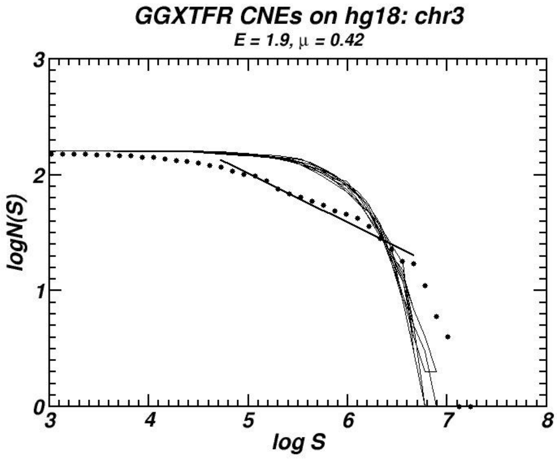

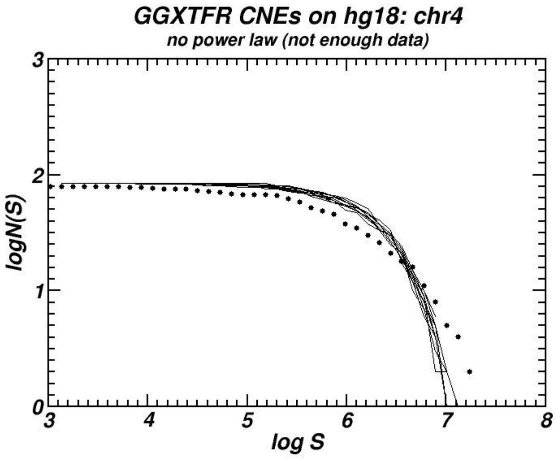

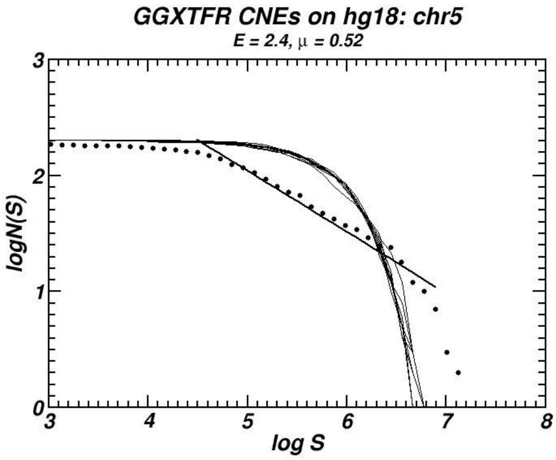

EU-GG-XT-FR CNEs (Dataset ie)

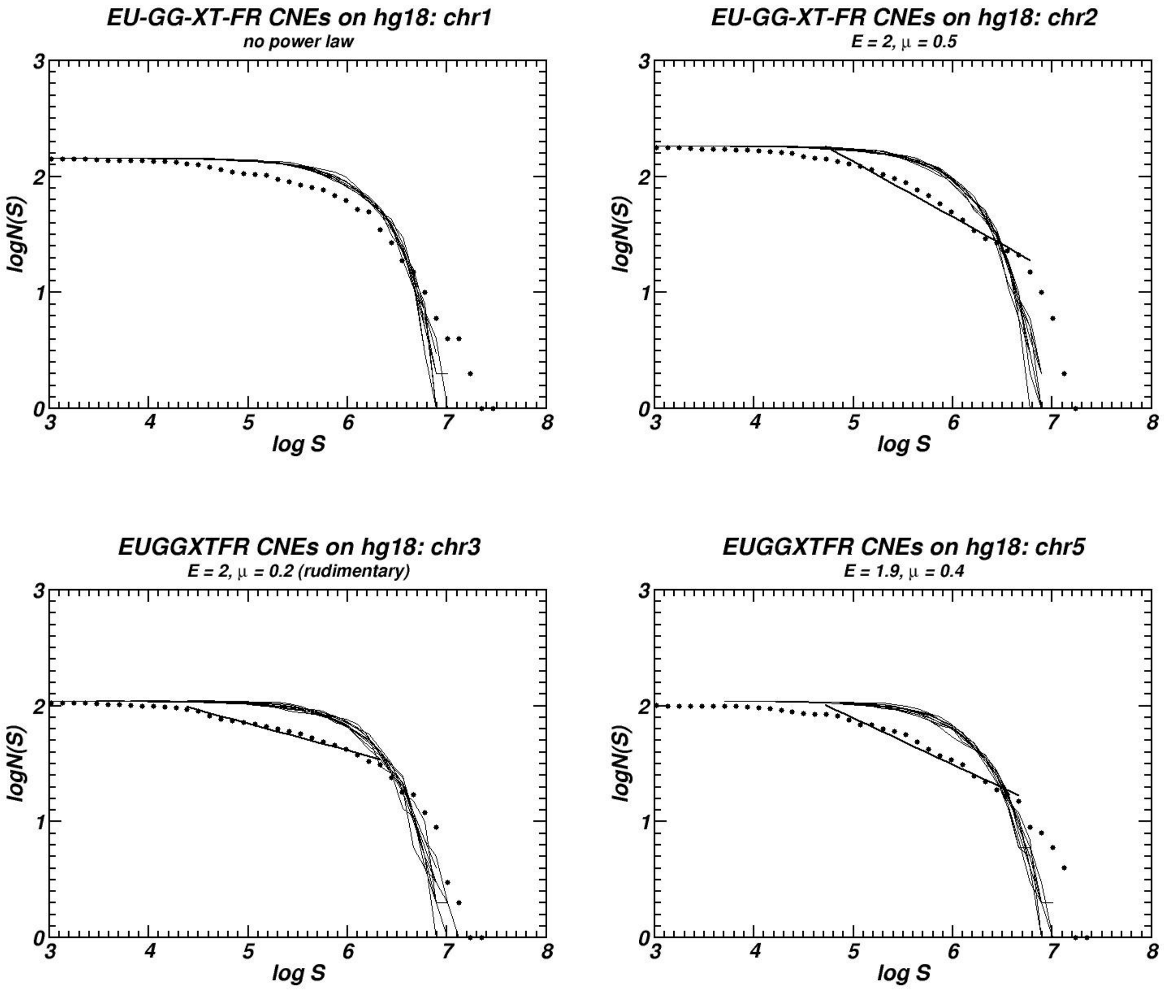

To obtain the figures from the merged and random datasets (adopted from Amniotic and Mammalian datasets as described in the text) shown below, we use the built-in function in R called sample (without replacement). Only the first five chromosomes are depicted :

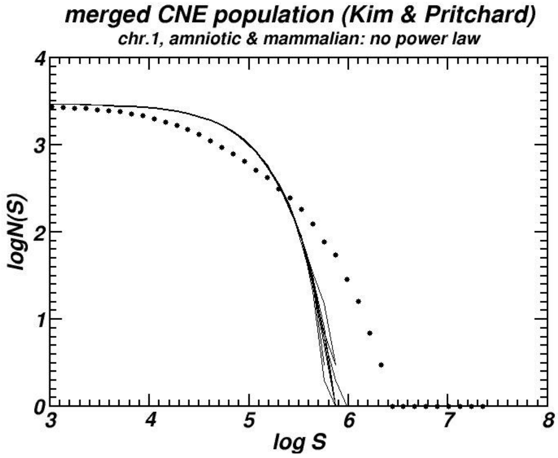

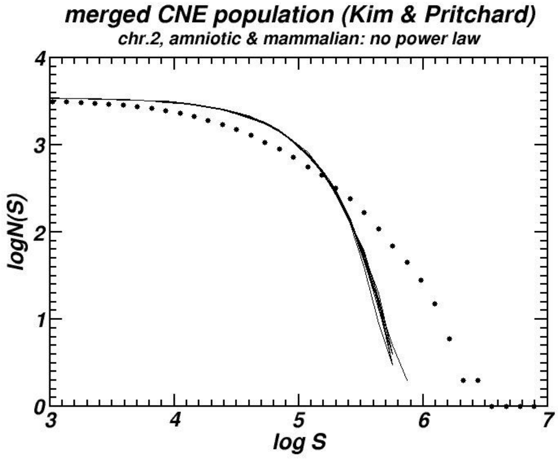

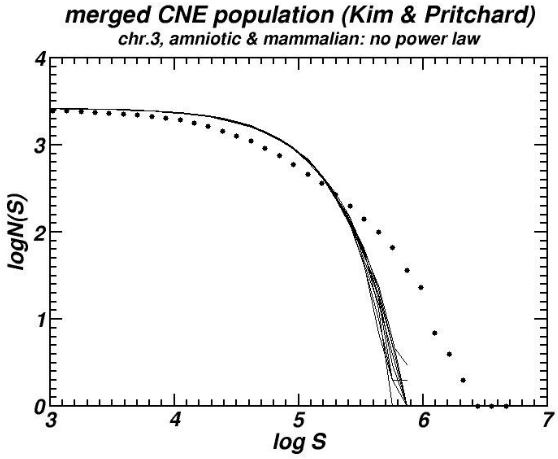

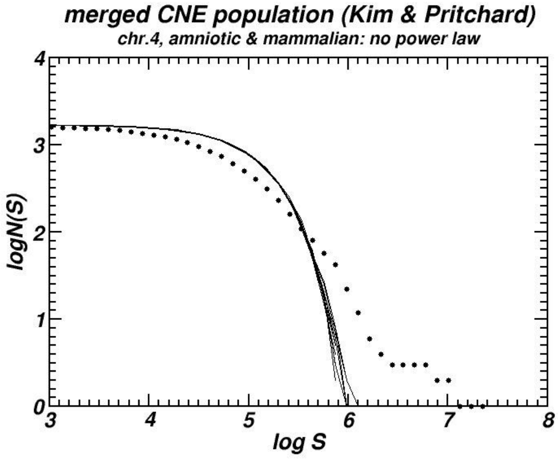

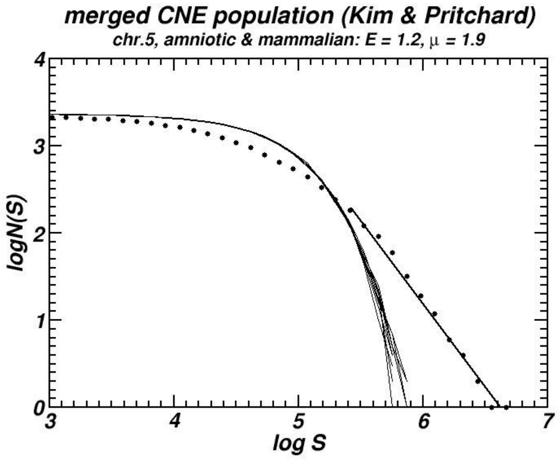

randomly selected mam. CNEs (Kim & Pritchard)
chr.1, population left eq. to amniotic: no power law

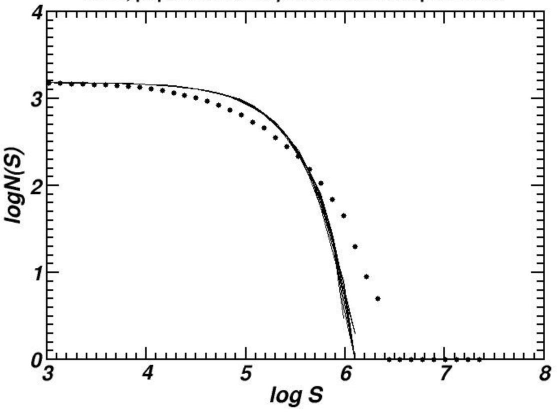

randomly selected mam. CNEs (Kim & Pritchard)
chr.2, population left eq. to amniotic: no power law

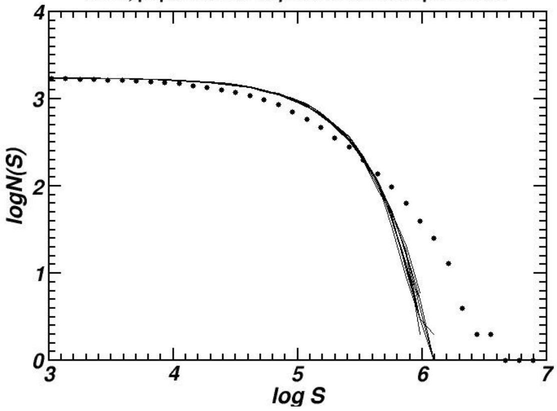

randomly selected mam. CNEs (Kim & Pritchard)
chr.3, population left eq. to amniotic: no power law

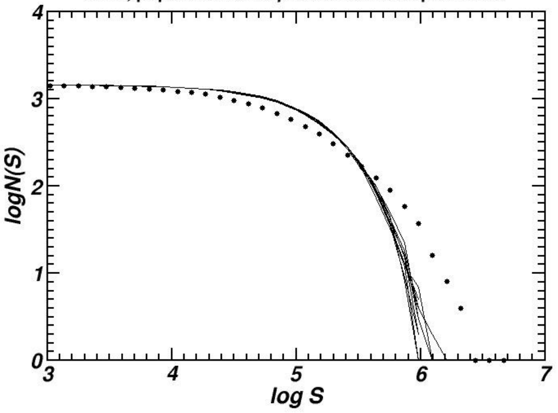

randomly selected mam. CNEs (Kim & Pritchard)
chr.4, population left eq. to amniotic: no power law

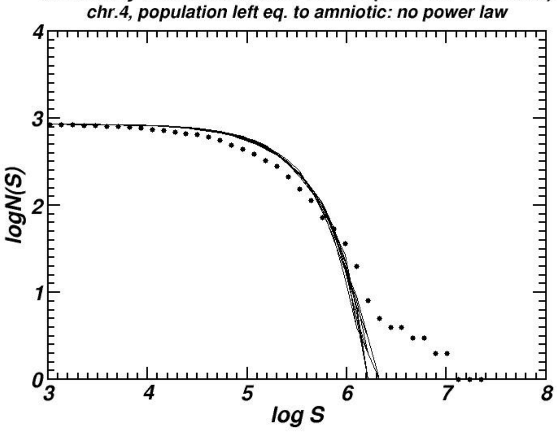

random mammalian CNEs on hg17: chr5
no power law

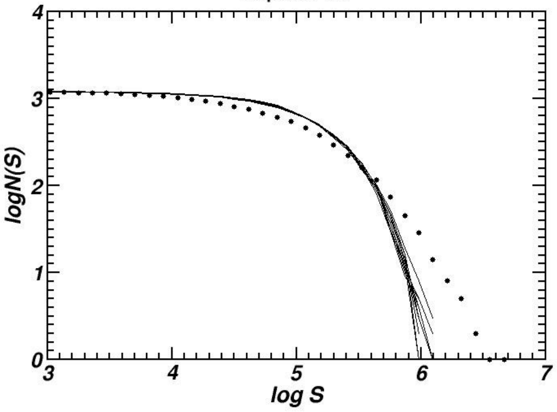

Appendix

Additional Simulations using the genomic duplications – CNE loss model

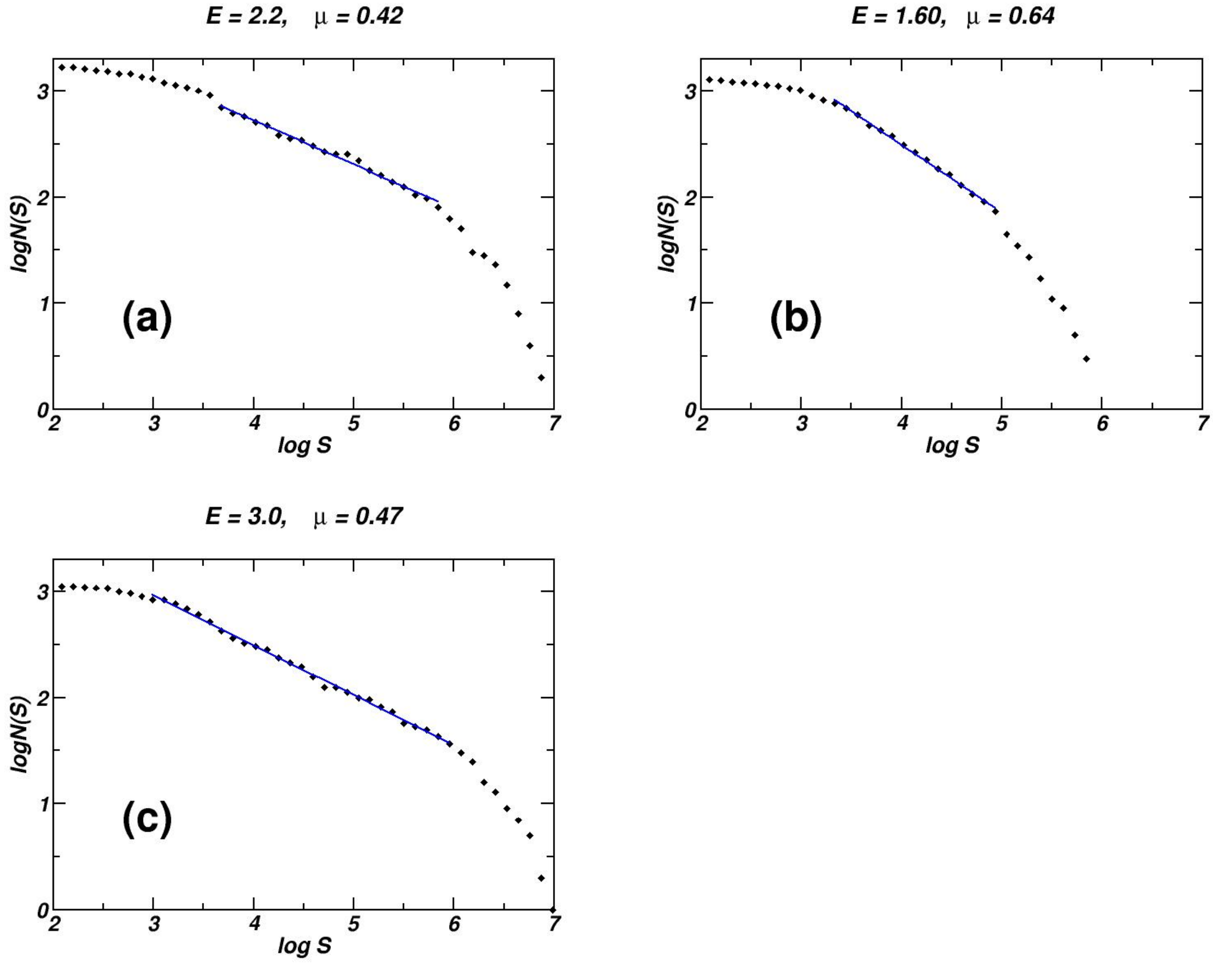

Simulations' details

Examples of simulations additional to the ones given in Figure 3 are shown. Initially, 1000 markers (representing CNEs) are randomly inserted in a sequence 2Mnt long. Then, in **(a)**, 173 segmental duplication events occurred, with lengths sampled from a uniform distribution with maximum the 5% of the actual length of the simulated sequence. After each such event a number of CNEs equal to 90% of the number of the duplicated ones are eliminated. The length reached by the simulated chromosome was ~195 Mnt (steps ***i*** and ***ii*** of the model). In **(b)** the segmental duplications were 87 (~50% less) and the final sequence length ~22 Mnt. In **(c)** the number of segmental duplications is as in **(a)** and the finally reached length ~119Mnt. Here, 256 additional events of non-duplicated CNEs eliminations are also allowed (step ***iii*** of the model). These eliminations represent cases of loss of function of ancestral CNEs with a subsequent progressive decomposition. For genomic and for model-generated size distributions, circles and rhombs are used respectively, throughout.
