# Supplementary material for: Conserved Noncoding Elements Follow Power-Law-Like Distributions in Several Genomes as a Result of Genome Dynamics
Source: PLoS One. 2014 May 2;9(5):e95437. doi: 10.1371/journal.pone.0095437 (PMC4008492; doi:10.1371/journal.pone.0095437)
Supplement: Plot S2 — “Plots” of EU100+ CNEs on 10 kb and 100 kb gene-masked genome (hg18). (PDF) [file pone.0095437.s004.pdf]

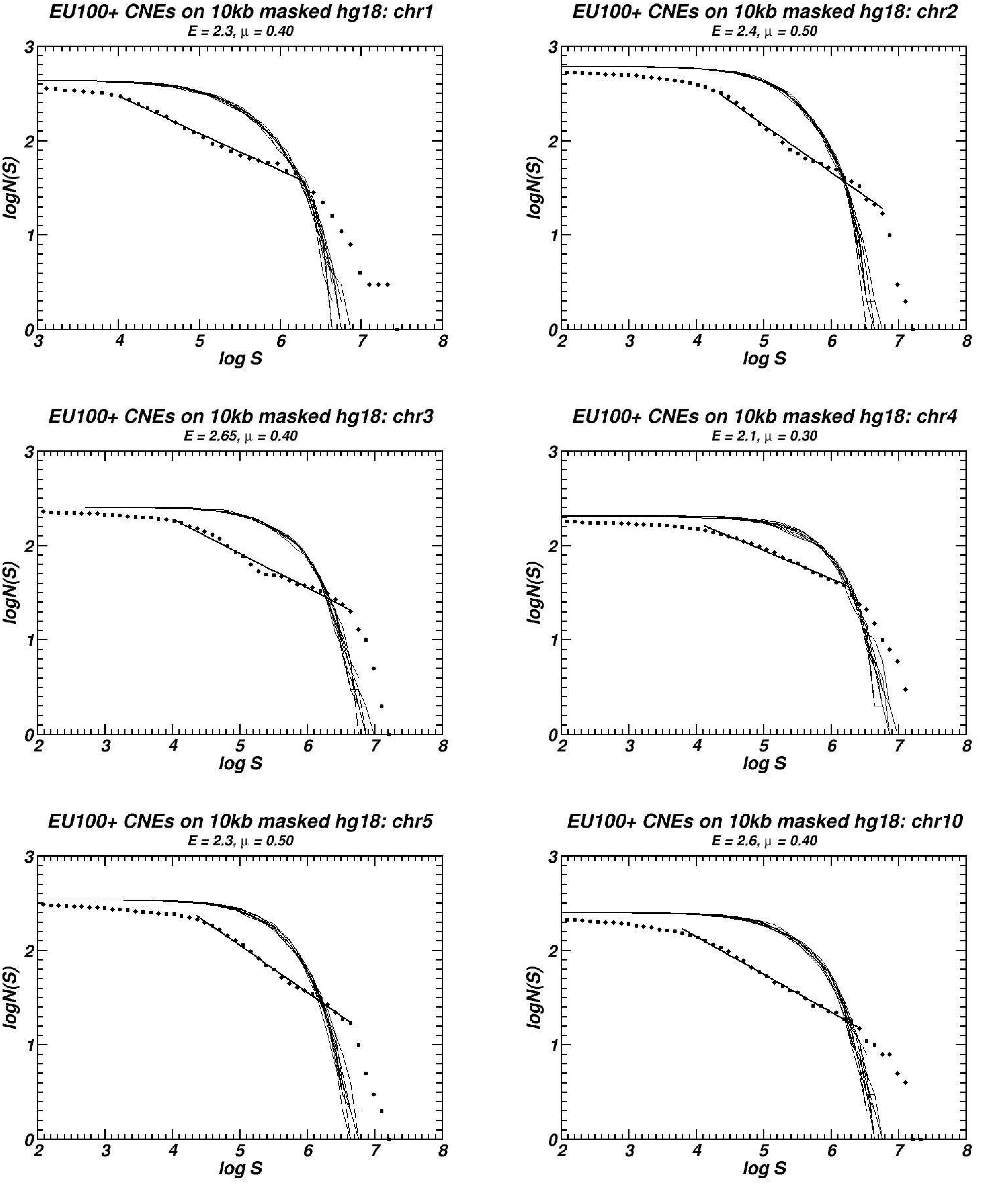

EU100+ CNEs on 10kb masked hg18: chr1
E = 2.3, μ = 0.40
EU100+ CNEs on 10kb masked hg18: chr2
E = 2.4, μ = 0.50
EU100+ CNEs on 10kb masked hg18: chr3
E = 2.65, μ = 0.40
EU100+ CNEs on 10kb masked hg18: chr4
E = 2.1, μ = 0.30
EU100+ CNEs on 10kb masked hg18: chr5
E = 2.3, μ = 0.50
EU100+ CNEs on 10kb masked hg18: chr10
E = 2.6, μ = 0.40
logN(S)
log S

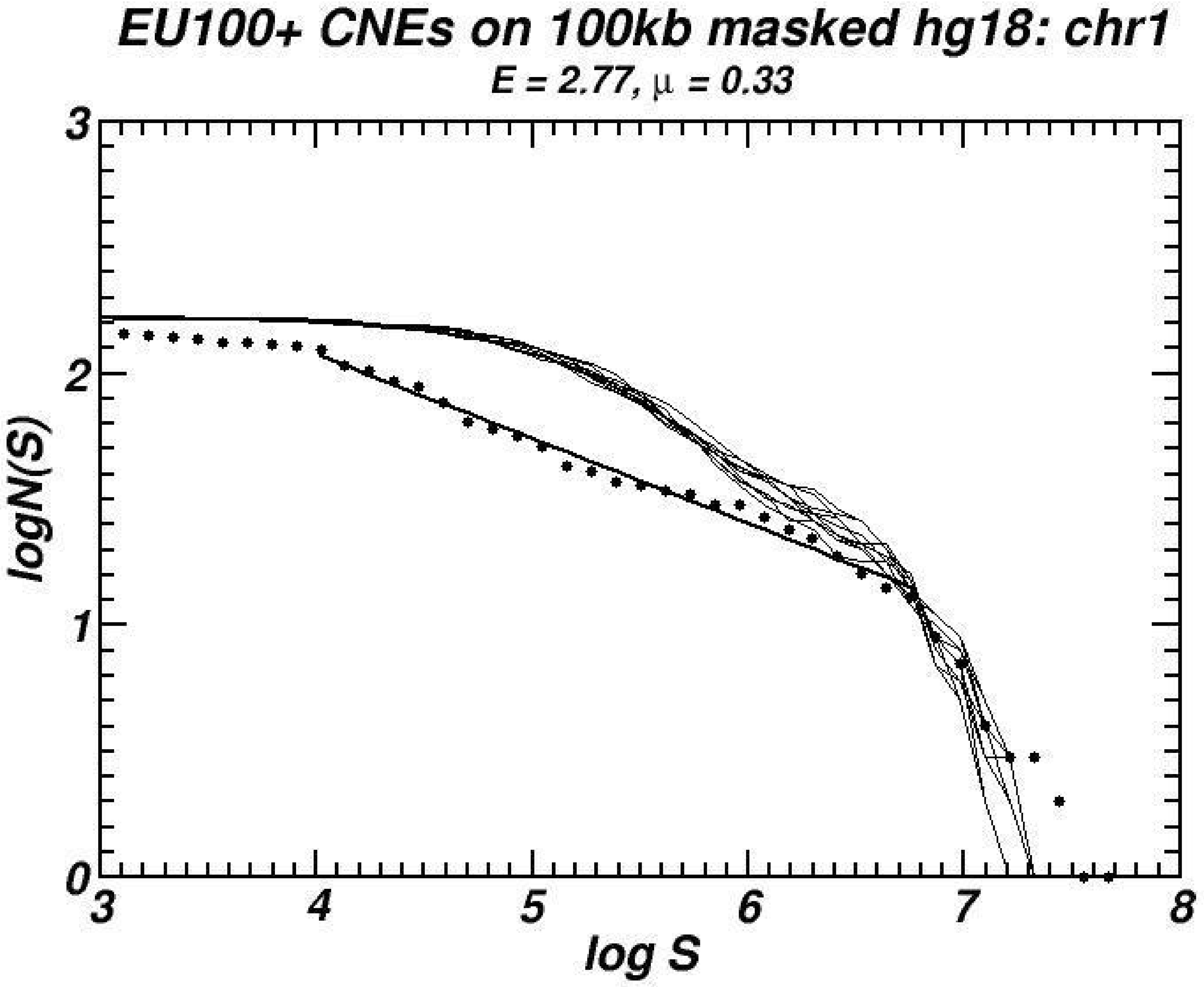
EU100+ CNEs on 100kb masked hg18: chr1
E = 2.77, μ = 0.33
logN(S)
log S

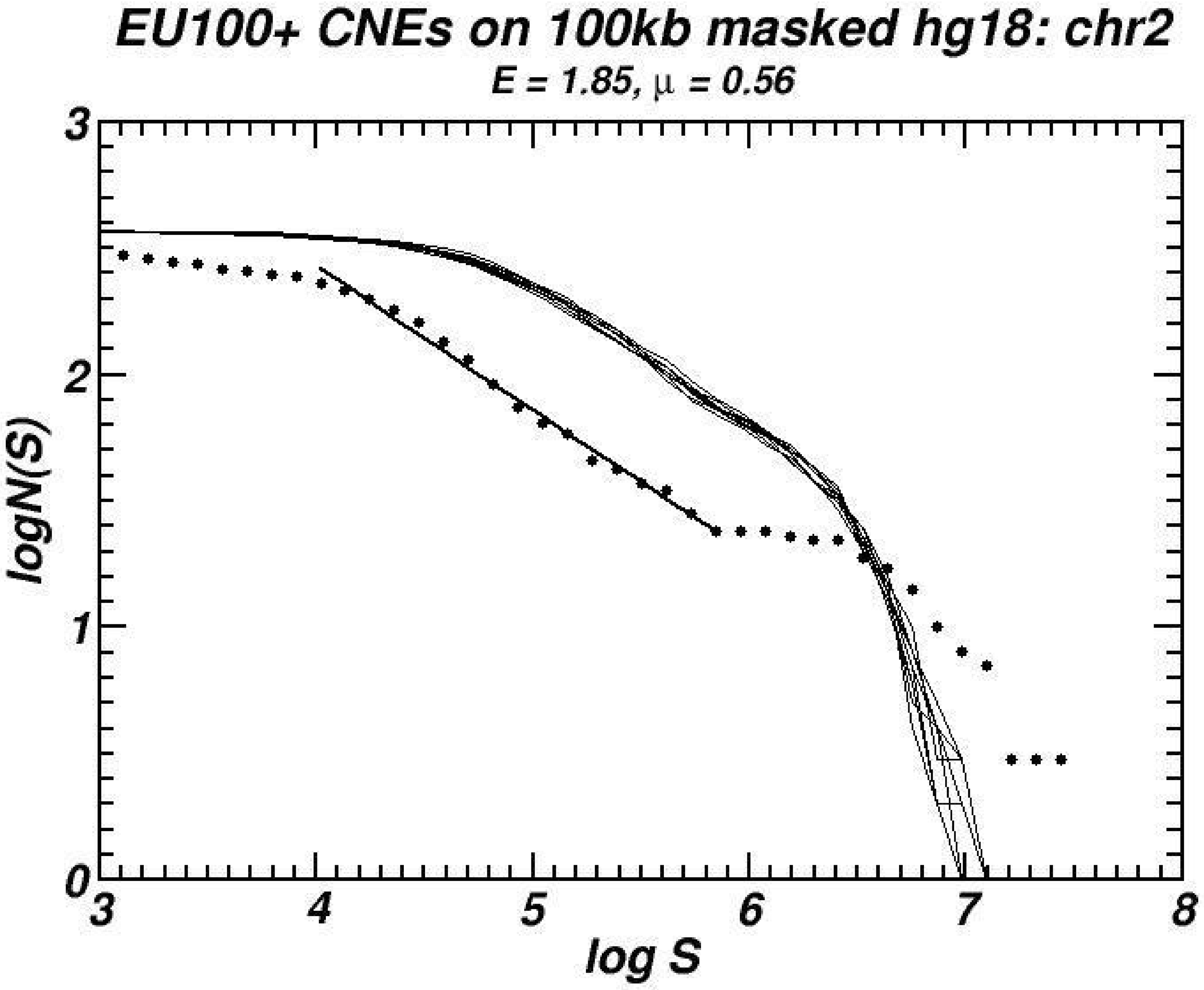
EU100+ CNEs on 100kb masked hg18: chr2
E = 1.85, μ = 0.56
logN(S)
log S

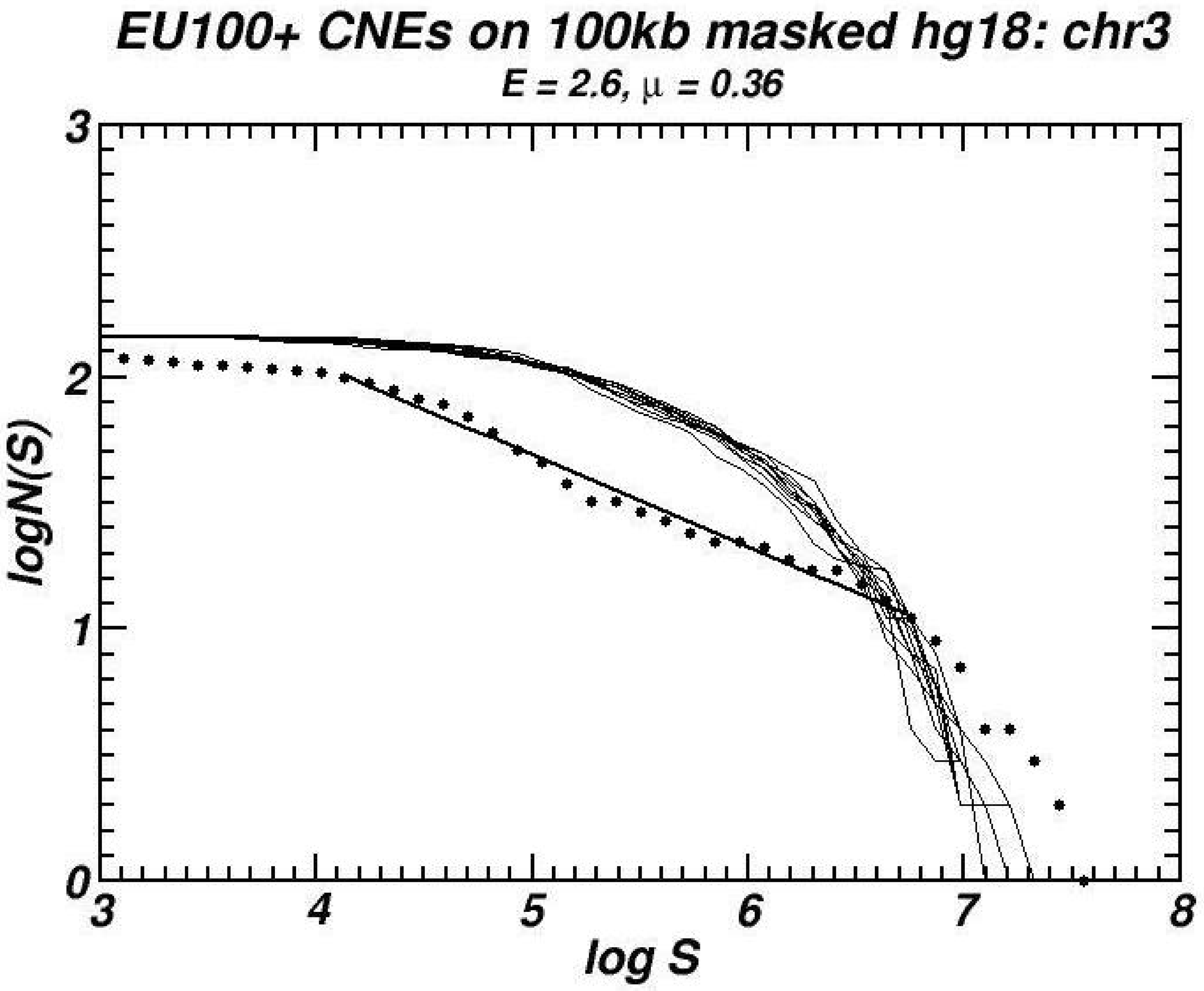
EU100+ CNEs on 100kb masked hg18: chr3
E = 2.6, μ = 0.36
logN(S)
log S

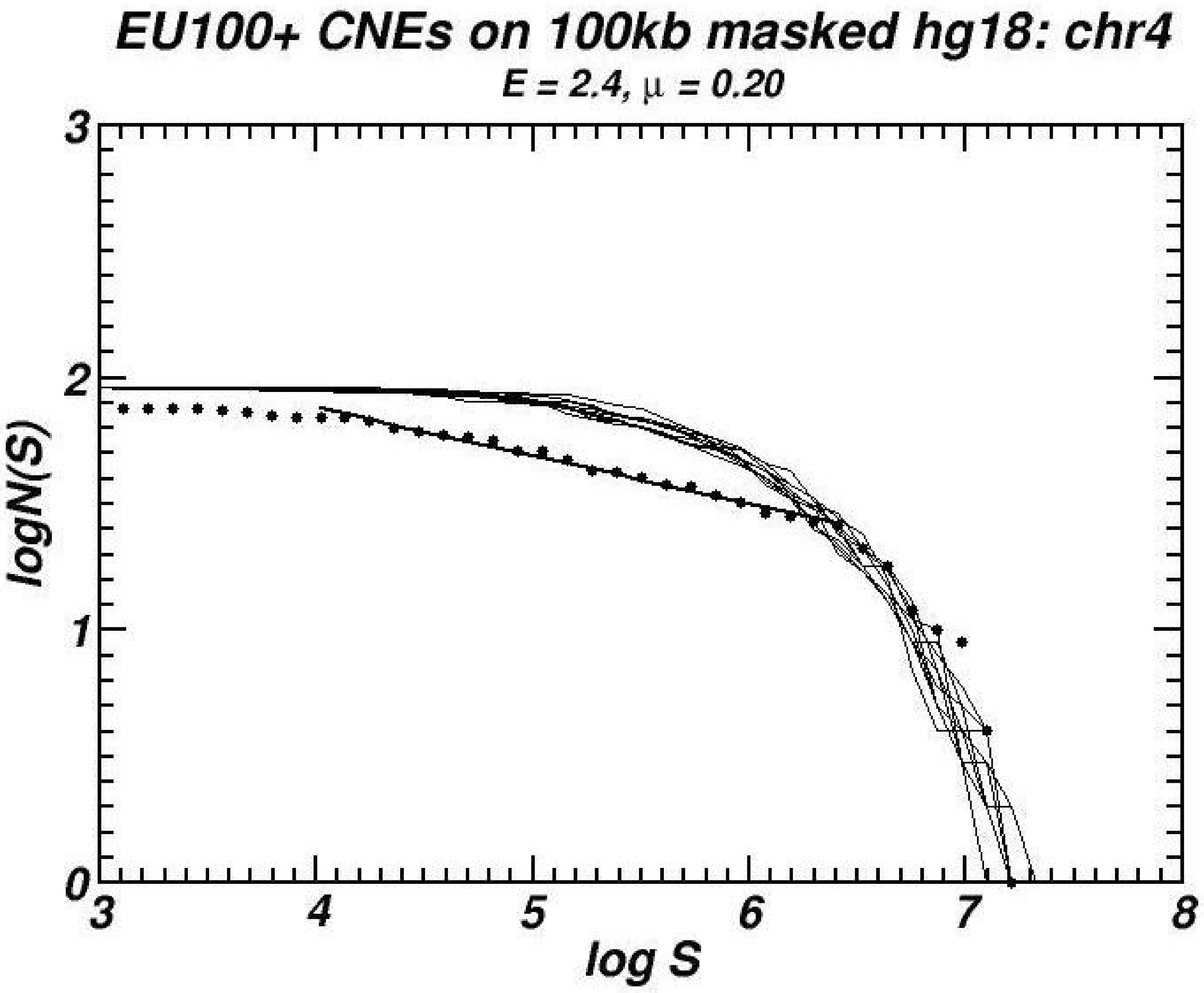
EU100+ CNEs on 100kb masked hg18: chr4
E = 2.4, μ = 0.20
logN(S)
log S

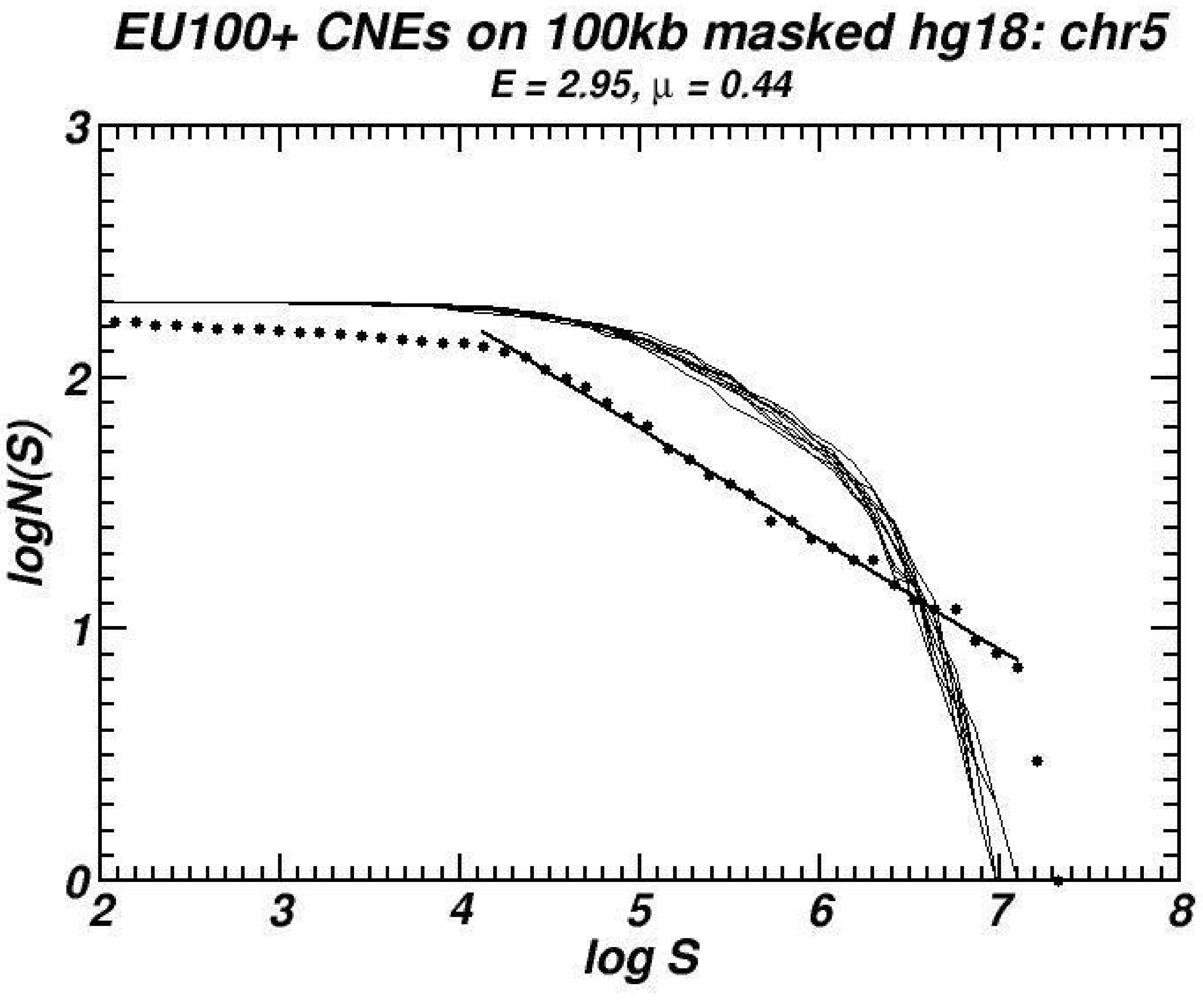
EU100+ CNEs on 100kb masked hg18: chr5
E = 2.95, μ = 0.44
logN(S)
log S

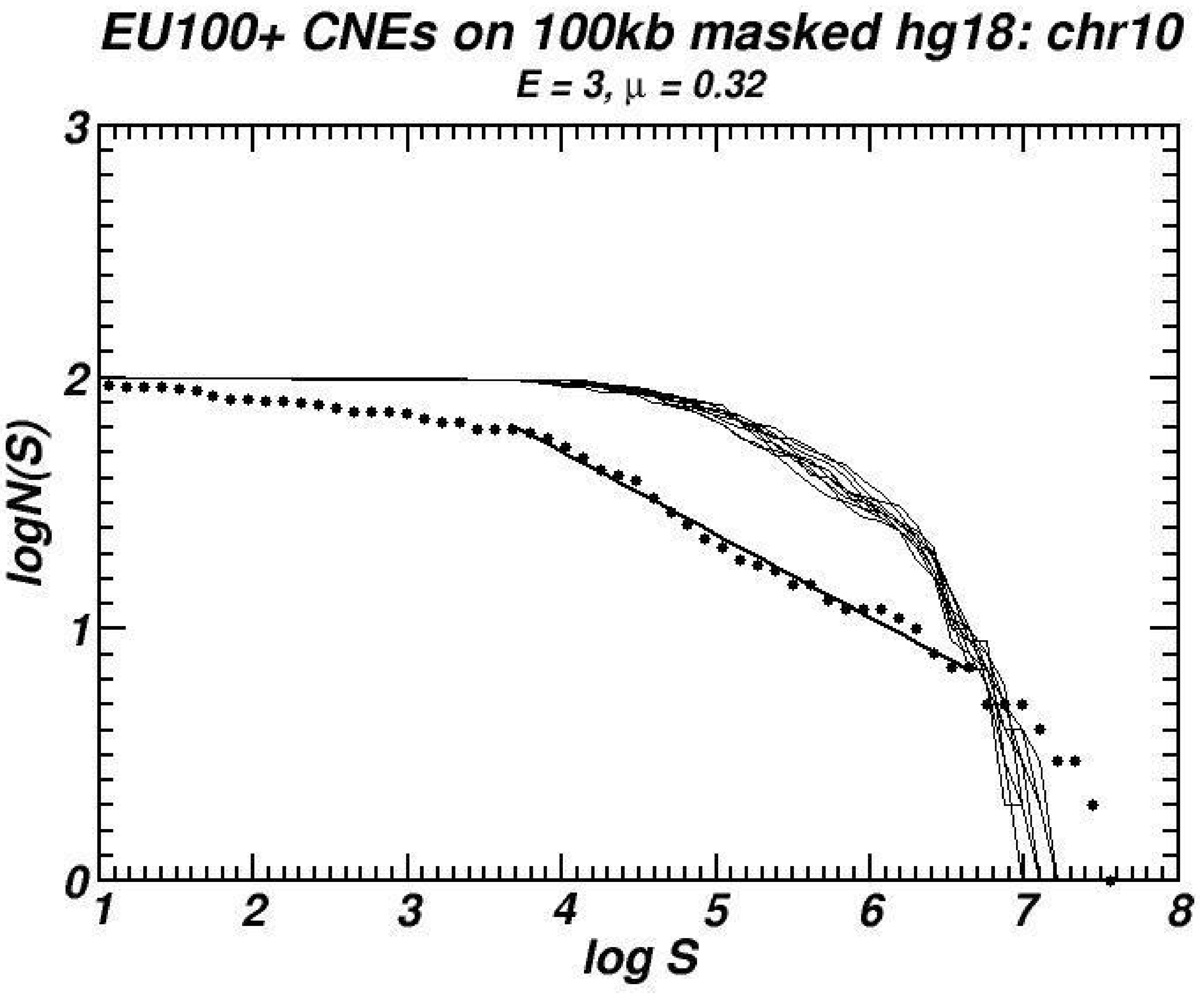
EU100+ CNEs on 100kb masked hg18: chr10
E = 3, μ = 0.32
logN(S)
log S
